# Targeted Sequence Capture Provides Insight into Genome Structure and Genetics of Male Sterility in a Gynodioecious Diploid Strawberry, *Fragaria vesca* ssp. *bracteata* (Rosaceae)

**DOI:** 10.1534/g3.113.006288

**Published:** 2013-08-01

**Authors:** Jacob A. Tennessen, Rajanikanth Govindarajulu, Aaron Liston, Tia-Lynn Ashman

**Affiliations:** * Department of Zoology, Oregon State University, Corvallis, Oregon 97331; ‡ Department of Botany and Plant Pathology, Oregon State University, Corvallis, Oregon 97331; † Department of Biological Sciences, University of Pittsburgh, Pittsburgh, Pennsylvania 15260

**Keywords:** pollen, sex chromosome, sex determination, trait mapping, translocations

## Abstract

Gynodioecy is a sexual system wherein females coexist with hermaphrodites. It is of interest not only because male-sterile plants are advantageous in plant breeding but also because it can be a crucial step in the evolutionary transition to entirely separate sexes (dioecy) from a hermaphroditic ancestor. The gynodioecious diploid wild strawberry, *Fragaria vesca* ssp. *bracteata* (Rosaceae), is a member of a clade with both dioecious and cultivated species, making it an ideal model in which to study the genetics of male sterility. To create a genetic map of *F. v*. ssp. *bracteata*, we identified informative polymorphisms from genomic sequencing (3−5x coverage) of two outbred plants from the same population. Using targeted enrichment, we sequenced 200 bp surrounding each of 6575 polymorphisms in 48 F1 offspring, yielding genotypes at 98% of targeted sites with mean coverage >100x, plus more than 600-kb high-coverage nontargeted sequence. With the resulting linkage map of 7802 stringently filtered markers (5417 targeted), we assessed recombination rates and genomic incongruities. Consistent with past work in strawberries, male sterility is dominant, segregates 1:1, and maps to a single location in the female. Further mapping an additional 55 offspring places male sterility in a gene-dense, 338-kb region of chromosome 4. The region is not syntenic with the sex-determining regions in the closely related octoploids, *F. chiloensis* and *F. virginiana*, suggesting either independent origins or translocation. The 57 genes in this region do not include protein families known to control male sterility and thus suggest alternate mechanisms for the suppression of male function.

The evolution of distinct male and female sexes (dioecy) is believed to often begin with the emergence of gynodioecy, the coexistence of females and hermaphrodites, in an ancestrally hermaphroditic population (Charlesworth and Charlesworth 1978; Spigler and Ashman 2012). Thus, the origins of separate sexes and sex chromosomes can be examined with studies of the genetic basis for male sterility and the selective pressures favoring it in naturally gynodioecious species (reviewed in Spigler and Ashman 2012; Charlesworth 2013). The adaptive benefit of male sterility in an otherwise-hermaphroditic individual is puzzling, as first noted by Darwin (1877), and several hypotheses have since been proposed to explain it (reviewed in McCauley and Bailey 2009; Dufay and Billard 2012). Classical hypotheses rest on an adaptive advantage for females, either from greater seed production as a result of their ability to redirect energy from male function or avoid antagonists (Ashman 2002), or from greater seed quality via avoidance of self-fertilization and thus inbreeding depression (Darwin 1877; Lewis 1941; Charnov *et al.* 1976; Charlesworth and Charlesworth 1978).

In either case, the magnitude of the benefit required for invasion and maintenance of females depends on the underlying genetics of male sterility (reviewed in Dufay and Billard 2012). In addition, gynodioecy can be “stochastic,” where its dynamics are governed largely by meta-population forces of migration and drift (Pannell 1997) as well as the genetic conflict between cytoplasmic male sterility (CMS) mutations that are beneficial to organelles but are not transmitted in the male gametes, and nuclear mutations that restore fertility (Rf) but may come with costs (reviewed in Bailey and Delph 2007; McCauley and Bailey 2009; Touzet 2012; Caruso *et al.* 2012). These hypotheses are not mutually exclusive, and more than one selective force could contribute to the sex ratio in a population (see Delph and Carroll 2001). Uncovering the genetic basis of male sterility can help to identify the likelihood of transitions to subdioecy (males, females, and hermaphrodites) and dioecy (reviewed in Spigler and Ashman 2012) and reveal new dynamics in the evolution of sex chromosomes (Charlesworth 2013), especially when studies are conducted among closely related species.

The genetic basis for male sterility has been identified in several agricultural and model plant taxa (*e.g.*, Ma 2005; Borg *et al.* 2009; Wilson and Zhang 2009; Lu *et al.* 2013), although most of these are rare or induced mutations in otherwise hermaphroditic species. Only a few studies have examined naturally occurring male sterility in wild species (*e.g.*, Dudle *et al.* 2001; Goldberg *et al.* 2010; Garraud *et al.* 2011). Most heavily studied cases are those with cytonuclear sex determination, in which the CMS loci typically encode toxic proteins, often chimeric ATP synthases, and the Rf loci are typically pentatricopeptide repeat (PPR) genes that function to silence the CMS mRNA (Hanson and Bentolila 2004; Wang *et al.* 2006). Some Rf loci belong to other protein families but still function by blocking the CMS locus (*e.g.*, Matsuhira *et al.* 2012). Only one copy of an Rf allele is generally necessary for CMS silencing (but see Schnable and Wise 1998; Kohls *et al.* 2011), so fertility restoration is typically dominant and thus male sterility at Rf loci is typically recessive (Chase 2007). Although there are Rf loci that act recessively in pollen (Wen *et al.* 2003; Fujii and Toriyama 2009), they act dominantly at the whole-plant level, that is, plants that are heterozygous at these loci are male fertile. Many nuclear male-sterility loci that do not interact with a CMS locus (*i.e.*, are non-Rf loci), are also known in plants, and these have been identified as encompassing diverse gene families such as acyl-CoA synthetase (Wang and Li 2009), plant homeodomain-containing protein (Alves-Ferreira *et al.* 2007), MYB transcription factor (Durbarry *et al.* 2005), brassinosteroid signaling genes (Ye *et al.* 2010), and heat shock proteins (Yang *et al.* 2009). At most of these loci, male sterility is recessive, although there are known cases of dominant male sterility (Cao *et al.* 2009) as well as more complex multiallelic systems (Lu *et al.* 2013). It remains unclear, however, which of these gene families, if any, underlie naturally occurring examples of gynodioecy.

The gynodioecious diploid (2n = 2×=14 chromosomes) wild strawberry *Fragaria vesca* ssp. *bracteata* (Rosaceae), native to western North America (Staudt 1989), is an ideal model for studying the genetic basis and evolution of male sterility (Li *et al.* 2012). It is classified as a subspecies of the otherwise hermaphroditic *F. vesca* ssp. *vesca*. However, the chloroplast genomes of Pacific Coast populations of *F. v*. ssp. *bracteata* share a closer phylogenetic relationship with several polyploid species than with other *F. vesca*, suggesting that it is the cytoplasmic donor to this octoploid clade (also known as clade ‘A’; Njuguna *et al.* 2013). The octoploid clade includes species displaying hermaphroditism (*F. iturupensis*), subdioecy (*F. virginiana*), and dioecy (*F. chiloensis*). These latter two octoploid species are the progenitor species of the cultivated strawberry *Fragaria* × *ananassa*. The agriculturally important Rosaceae family also includes other examples of male sterility (*e.g.*, plum, peach and blackberry; Dorsey 1919; Connors 1926; Scott and Weinberger 1938; Crane and Lawrence 1931; Jordano 1993). Previous classical genetic work in octoploid strawberry (*F. virginiana* and *F. chiloensis*) and in *F. v*. ssp. *bracteata* concluded that female function is determined by a disomically inherited single nuclear locus (Valleau 1923; Ahmadi and Bringhurst 1989) in all three species with male sterility dominant, that is, females are heterozygous and hermaphrodites are homozygous at this locus.

Recent genetic mapping in the octoploid species and their natural hybrid confirms that male sterility is dominant to male fertility but also that it shows linkage to female fertility (Goldberg *et al.* 2010; Spigler *et al.* 2011; Govindarajulu *et al.* 2013). Existing observations support conflicting hypotheses about the evolutionary origin of male sterility. On one hand, the close relationship among several species displaying the rare trait of dominant male sterility suggests a single origin of the trait, with possible translocation (Goldberg *et al.* 2010). On the other hand, the observation that male sterility maps to different genomic regions in the polyploid *Fragaria* species (Goldberg *et al.* 2010; Spigler *et al.* 2011; Govindarajulu *et al.* 2013), as well as the existence of hermaphroditic *F. iturupensis* within an otherwise-sexually dimorphic clade (Njuguna *et al.* 2013), suggest that male sterility may have evolved several times independently. Moreover, equivocal evidence for a fitness advantage sufficient to maintain females (*i.e.*, high selfing rates in hermaphrodites but no seed quantity advantage and low- and variable-quality advantage in early life stages; Li *et al.* 2012; Dalton *et al.* 2013) under the published mode of sex determination (*i.e.*, dominant nuclear male sterility; Ahmadi and Bringhurst 1989) raises the possibility for alternate means of sex determination. Thus, a detailed genetic mapping approach is required to assess the inheritance of sex determination in *F. v*. ssp. *bracteata*.

Mapping sex-determination loci has been challenging traditionally because of the suppression of recombination on sex chromosomes and the low power of limited-marker, pregenomic studies; therefore, few high-resolution maps have been produced (Charlesworth and Mank 2010; Charlesworth 2013). However, there are several key reasons why high-resolution mapping *F. v*. ssp. *bracteata* is now especially feasible. First, suppression of recombination is not expected before the appearance of distinct linked male-function and female-function loci (Charlesworth 2013). Second, the small *F. v*. ssp. *vesca* genome has been sequenced and annotated (Shulaev *et al.* 2011), providing an *a priori* expectation of marker order as has been used in other reference-guided linkage maps (*e.g.*, Felcher *et al.* 2012), as well as illuminating translocations and recombination rates that can be important hallmarks of evolving sex determining regions (Charlesworth 2013). Third, new high-throughput approaches such as RAD (restriction-site associated DNA) mapping (Baird *et al.* 2008), highly parallel single-nucleotide polymorphism (SNP) genotyping (Fan *et al.* 2003), and targeted capture (Cronn *et al.* 2012) allow thousands of markers to be readily assessed in multiple individuals. Although sex loci have been mapped with RAD (Anderson *et al.* 2012) and highly parallel SNP genotyping (Bradley *et al.* 2011), targeted capture has not been used previously. However, targeted sequence capture presents several advantages for linkage mapping including efficient data generation, the ability to target SNPs known to be informative in the parents, and low individual variation in locus recovery. It also provides a rapid means for comparative analyses of genome structure between related species.

The goals of the study presented here were as follows. First, to genetically map the male-sterility locus in *F. v*. ssp. *bracteata* to a narrow genomic region to test for synteny with other *Fragaria* species, to determine the mode of inheritance, and to identify possible candidate genes. Second, to demonstrate the power of a new technology—targeted capture using RNA baits—for studying genomic variation in plants (Cronn *et al.* 2012). Third, to compare a high-density *F. v*. ssp. *bracteata* linkage map with the *F. v*. ssp. *vesca* reference genome (Shulaev *et al.* 2011) to assess genomic evolutionary phenomena such as recombination rate, which can be unusually high or low near sex-determining regions (Hasselmann and Beye 2006; Charlesworth 2013) but has not been well studied in incipient sex loci, as well as translocations, which can obscure patterns of synteny and contribute to the striking genomic lability in the location of sex-determining loci (Charlesworth and Mank 2010).

## Methods

### Species description and map cross population

*F. vesca* ssp. *bracteata* (Rosaceae) is a herbaceous perennial plant occurring from the Rocky Mountains to the Pacific Northwest and extending from British Columbia to southern Mexico (Staudt 1989). Both parental plants for this study were collected from a single population on Marys Peak, Oregon, (N44°29′18.4″, W123°32′14.7″) and were maintained in the greenhouse at the University of Pittsburgh.

To map male sterility, we created an F1 mapping population by pollinating a female (MRD30) with pollen from a hermaphrodite (MRD60) in 2011. Seeds (*N* = 127) from this cross were planted in Sunshine germination mix: Fafard #4: sand, and exposed to 14-hr day and 19°/15° day/night temperatures with a relative humidity of 70% in a growth chamber. Seedlings (N = 112) were transplanted into 200-mL pots filled with a 2:1 mixture of Fafard #4 and sand and grown at 21°/15° day/night temperatures and 12-hr daylight. To induce flowering 4 mo after planting, plants were fertilized with bloom booster 10:30:20 N:P:K fertilizer (Scotts Miracle-Gro Company, Marysville, OH) and exposed to a cold/dark treatment with 10-hr day and day/night temperatures of 18°/10° for 3 wk, 18°/4° for 7 wk, and 10°/4° for 2 wk. This treatment was repeated up to two times. All plants were watered and protected from pests as needed.

### Scoring male function

Male function was scored on each flowering plant on at least two flowers formed at two separate times. Male function was determined as the presence (fertile) or absence (sterile) of pollen production. Anthers visibly dehiscing pollen were scored as fertile whereas those not dehiscing (*i.e.*, visibly shedding pollen) were confirmed as sterile by observing crushed anther sacs under the microscope. Viability of pollen grains of male-fertile individuals was confirmed via vital stains (*e.g.*, Alexander’s stain; Kearns and Inouye 1993). Six progeny died before flowering and 11 have not yet flowered, so we were able to score male function on a total of 95 progeny.

### DNA extraction and library preparation

Total genomic DNA was extracted from ca. 100- to 150-mg sample of fresh leaves from parents (MRD30 and MRD60) and 48 progeny using a modified CTAB procedure (Doyle and Doyle 1987). Extracted DNA samples were quantified using the Qubit dsDNA HS assay (Life Technologies/Invitrogen, Carlsbad, CA). Library construction, solution hybridization, and nucleotide sequencing followed MycroArray MyBait and Illumina protocols, with minor modifications. For library construction, 1−2.5 µg of DNA was sheared using a Diagenode BioRuptor Sonicator for 13 min. After sonication, a small aliquot was assayed by gel electrophoresis, and additional sonication was conducted if the sample did not have a median shear size of approximately 200 bp. Subsequent enzymatic steps used NEBNext (New England Biolabs, Ipswich, MA) reagents. For low-coverage genome sequencing of the paternal parent (MRD60), a custom internal index adapter (Cronn *et al.* 2008) was used, whereas the target capture libraries (parents and progeny) used external index adapters (Bio Scientific, Austin, TX). The low coverage genome library of the maternal parent (MRD30) was sequenced in one lane of the Illumina GAIIx with 80 bp single end sequencing, whereas MRD60 was sequenced as part of a 13-plex on the Illumina HiSeq 2000 with 101 bp paired end sequencing. For the target capture libraries, agarose gel size-selected fragments (ca. 200-bp inserts) were amplified for nine cycles (minimized to avoid polymerase chain reaction duplicates) using Bio Scientific primers. Following Qubit (Invitrogen/Life Technologies, Eugene, OR) fluorometric quantitation, 30−60 ng of each library was pooled in an 8-plex or 12-plex. Each multiplex pool (400−480 ng) was used in a single solution hybridization (=target enrichment) reaction, and incubated at 65° for 36−40 hr with the RNA baits. After incubation, the target-hybridized biotinylated RNA baits were captured with streptavidin-coated magnetic beads. The captured DNA targets were then eluted in water and amplified by polymerase chain reaction for 9−12 cycles. After purification and quantitation, the target enriched multiplex samples were sequenced as an 8-plex or a 48-plex (Supporting Information, Table S1) with 101-bp single end sequencing on the Illumina HiSeq 2000 at Oregon State University. Sequencing data in FASTQ format was uploaded to SRA (Accession SRP022950).

### Genotyping by targeted sequence capture

We filtered Illumina reads by converting bases with Phred quality scores less than 20 to missing (N), and excluding reads with fewer than 75 high-quality (≥20) bases. The low-coverage genomes of the parental plants were mapped using Burrows-Wheeler Aligner, or BWA (Li and Durbin 2009) to the *F. v*. ssp. *vesca* reference genome (v. 1.1, based on accession Hawaii 4; Shulaev *et al.* 2011), masked with RepeatMasker; Smit *et al.* 1996−2010). This reference genome (hereafter designated FvH4) consists of seven pseudochromosomes assembled from an interspecies linkage map (FvH4_1-FvH4_7) ranging from 21 to 39 Mb in size, as well as a 12-Mb concatenation of unanchored scaffolds (FvH4_0), totaling 207 Mb (Sargent *et al.* 2011; Shulaev *et al.* 2011). We converted parental genotypes to vcf format for analysis (Danecek *et al.* 2011). We identified polymorphic sites in the two parents (SNPs and 1- to 2-bp indels, both coding and noncoding) and designed custom MYbaits (MYcroarray, Ann Arbor, MI) biotinylated RNA baits targeting 6575 of these sites (6376 SNPs and 199 indels; File S1). Each polymorphism is targeted by three 100-bp oligonucleotides, one flanking each side of the polymorphism and one centered on the polymorphism, such that the 100-bp surrounding the polymorphism is targeted twice. We chose polymorphisms that were likely informative (heterozygous in one parent only based on ≥5x coverage in our genomic data), >100 bp away from other polymorphisms, and in regions of intermediate GC content (30–70%) to maximize genotyping reliability. We also minimized marker clustering by excluding sites that were <5 kb from another included polymorphism in one direction and <10 kb from another included polymorphism in the opposite direction.

We randomly chose 48 of the F1 offspring for linkage mapping. Examination of a previous reference genome version (v. 1.0) revealed portions of two scaffolds (scf0513160 positions 1−1,989,403, hereafter scA, 2.0Mb; and scf0513158 positions 2,258,778−4,674,928, hereafter scB, 2.4Mb) that had been left out of FvH4 (v. 1.1). Although we had not designed targeted capture probes for these scaffolds, we mapped our reads to a supplemented reference genome that included these scaffolds, again using BWA (Li and Durbin 2009), and converted genotypes to vcf format for analysis. Because the sum of all baits was less than 1% of the genome, we could sequence targeted regions at very high coverage for very accurate genotyping. Thus, we first filtered for a per-individual depth of at least 20, then we retained genotypes as valid only if the Phred-scaled likelihood was 0 and Phred-scaled likelihood for all other potential genotypes was >40 (likelihood of other genotype < 10^−4^); otherwise, genotypes were considered missing. To maximize the number of high-quality, true polymorphisms in our linkage map, we only retained putative polymorphisms for linkage mapping if they met several strict criteria, including showing missing genotypes in no more than eight offspring, not showing >85% of offspring with the same genotype, and having informative parental genotypes consistent with segregation in the offspring.

### Linkage mapping

We used the R package OneMap (Margarido *et al.* 2007) to generate linkage maps for informative markers in each parent (hereafter referred to as FvbPat for paternal and FvbMat for maternal, or Fvb for the set of linkage groups). Because our markers had known locations on the reference genome (Shulaev *et al.* 2011), we had an *a priori* hypothesis for their relative positions, as with other reference-guided linkage maps (*e.g.*, Felcher *et al.* 2012). Thus, we could justifiably use relatively liberal criteria for linkage map construction, and any features of the linkage map that deviated from the expected pattern, such as the number of linkage groups or marker order within groups, we examined individually for confirmation. Initially we only used markers with no missing genotypes to create the framework linkage maps; markers with missing data were added manually later if possible. We initially used a LOD (logarithm of odds) of 3 to assign markers to linkage groups, and if this resulted in fewer than the expected seven linkage groups we raised the LOD to 4. We used a LOD of 2 to order markers within linkage groups. Markers that could not be placed unambiguously at a LOD of 2 were assigned to the most likely position on the basis of their LOD score. Linkage map results were carefully curated manually to correct for results attributable to genotyping error and to resolve ambiguities. For example, the observation of paired recombination events on either side of a single marker is likely to be a genotyping error rather than a real double recombination event and was treated as such. This approach is conservative because ignoring any true double recombination event on either side of a polymorphism can only decrease our estimates of centimorgan distances slightly and would not qualitatively affect our results. We estimated genotyping error rate using these anomalous genotypes that did not match the expected genotype given the genotype of the same individual at sites upstream and downstream on the linkage map (Figure S1). Using polymorphisms heterozygous in both parents and the locations of markers in FvH4, we aligned the linkage groups between the parents.

We compared the FvbPat and FvbMat linkage maps with FvH4 (visualized with Circos, Krzywinski *et al.* 2009). We assigned each linkage group a number (1−7) corresponding to the FvH4 pseudochromosome with which it shared the most markers. Interchromosomal translocations were identified as sets of contiguous markers with FvH4 location that did not match the linkage (Fvb) location. Intrachromosomal translocations were identified as sets (≥4) of contiguous markers with the same chromosome number but with an Fvb location that was incompatible with their FvH4 location. Inversions were identified as increases in linkage map distance in the opposite direction from reference genome distance, with at least two observed map locations supporting the directional change, such that a recombination event that reverses that previous recombination event was considered to be a possible double recombinant and not an inversion. To examine variation in recombination rate across the genome, we divided Fvb linkage groups as closely as possible into sections of 1 Mb, a size chosen because our population size would not allow accurate estimates of recombination rate in sections less than 100 kb, and recombination rate might vary substantially across sections of 10Mb (Schnable *et al.* 1998; Li *et al.* 2007). We did not attempt to estimate recombination rate across the borders of translocations and inversions, so we assessed contiguous segments defined by these genomic rearrangements, and segments larger than 1 Mb were broken into equal sections less than 1 Mb each. We defined the recombination rate as the ratio of Fvb linkage map distance (cM) to reference genome sequence distance (Mb), following other studies that compare linkage maps and genome sequences between closely related taxa (*e.g.*, Meznar *et al.* 2010), and thus this represents a rate that assumes congruence in physical distance between Fvb and FvH4. Variation in this rate across the linkage map thus could reflect disparities in genome architecture between the subspecies, *i.e.*, unobserved indels and rearrangements.

### Mapping male sterility

Our *a priori* expectation based on published accounts (Ahmadi and Bringhurst 1989) was that male sterility is determined by a single, perfectly penetrant Mendelian locus, and therefore our sample size would be sufficient (Lander and Botstein 1989) to initially identify genomic regions of major effect consistent with this prediction that could be confirmed and fine-mapped using additional individuals. We coded male sterility as a Mendelian locus (*e.g.*, females AB and males AA), and mapped it along with the targeted capture genotypes using OneMap to determine its position on the linkage map. For confirmation and fine-mapping, we identified nine informative polymorphisms in this region using the low-coverage genome sequences of the parents, and we genotyped these sites using Sanger sequencing in both the phenotyped targeted capture offspring (n = 40) as well as additional offspring from this cross (n = 55) with known sexual phenotypes (primers in Table S2). Genes in the region matching male sterility were identified using the Strawberry Genome Hybrid Gene Model Database, Version 2 (http://strawberrygenome.org).

## Results

### Genotyping by targeted sequence capture

**Figure 1:**
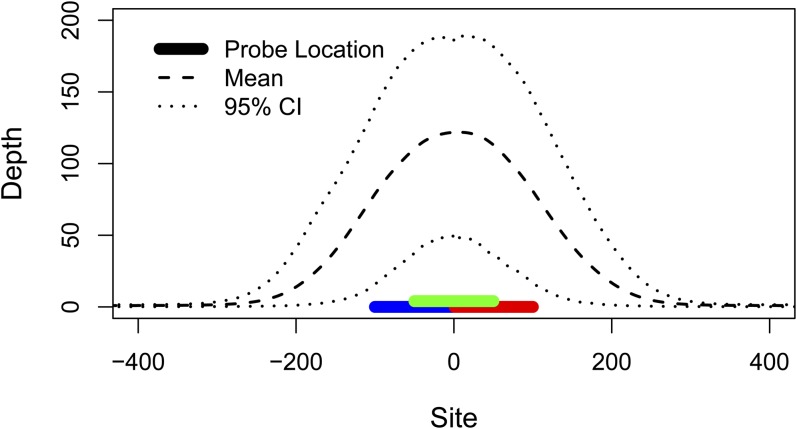
Average targeted capture sequencing depth in all 50 individuals across all 6575 targeted polymorphisms (Table S1, File S1, File S2). Colored bars represent the three probes designed for the targeted site. Dashed and dotted lines depict mean and 95% interval of sequencing depth. Mean sequencing depth of targeted sites was 120x, and high-coverage sequence was obtained even beyond the ends of the probes (mean depth 100 bp from probes = 15x).

**Figure 2:**
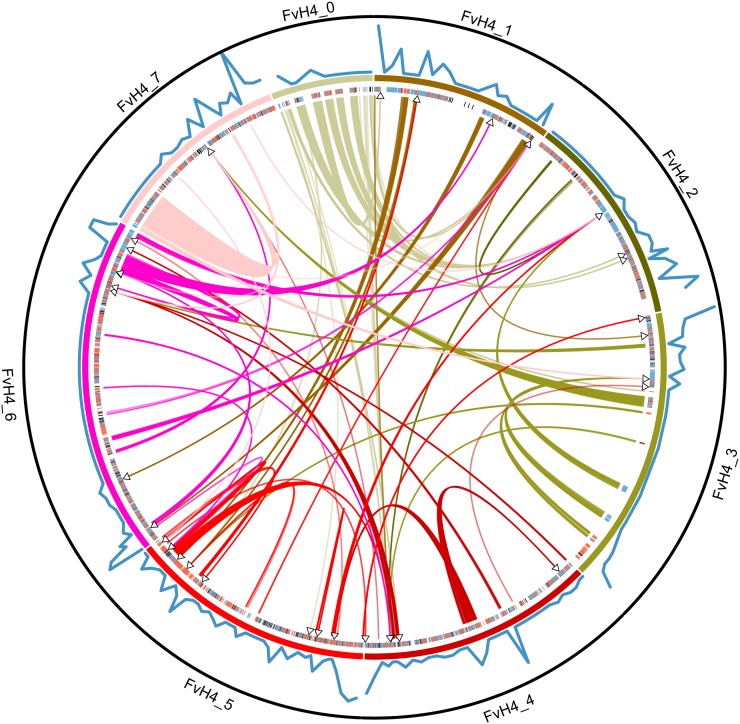
Circular map of *F. vesca* ssp. *vesca* reference genome (FvH4; pseudochromosomes 0−7 are displayed in a circle) with *F. vesca* ssp. *bracteata* genomic data (Fvb) indicated (created with Circos; Krzywinski *et al.* 2009). Targeted capture markers polymorphic in the mother (blue), father (red), or both (black) are indicated as tick marks just inside the chromosomes. Translocations identified in our Fvb linkage map are indicated by curved links between different regions of FvH4, colored to match their location on FvH4, with arrowheads indicating their estimated position in Fvb. Recombination rate is indicated by a blue line along the outside of the chromosomes. The maximum value observed for recombination rate (20.4 cM/Mb) is indicated by the black circle encompassing the figure. Scaffolds scA and scB not shown.

Mean coverage (read depth) of the parental genomes was 3.2x for the paternal parent (MRD60) and 5.4x for the maternal parent (MRD30) across 149 Mb. We observed 184,531 polymorphisms within the parents. In the targeted capture data, mean depth per individual at the 6575 targeted sites was 120x (median depth per individual = 102x; minimum individual depth = 35x; Figure 1, Figure S1). A mean depth of at least 20x was observed at 6414 (98%) of these sites, and 5417 (82%) of these were polymorphic and met our further quality criteria for inclusion in the linkage mapping. An additional 1.92 Mb passed our depth and quality filters, exceeding the number of targeted base pairs by nearly 50% due to coverage in the “splash zones” flanking the targets (Figure 1). Thus, in addition to the targeted polymorphisms, we added 2385 polymorphisms for a total of 7802 high-quality markers for linkage mapping (Figure 2, File S2, File S3). We observed low per-individual rates of both missing data (mean = 2.1%; median = 0.6%) and genotyping errors (mean = 0.2%; median = 0.05%) across the dataset (Figure S1).

### Linkage mapping

We produced seven paternal linkage groups at LOD 3 for MRD60 (FvbPat_1-FvbPat_7), corresponding to the haploid base number of chromosomes in *Fragaria* (Figure S2). All linkage groups remained intact at LOD 4, except FvbPat_1 and FvbPat_4 were each split into two groups (FvbPat_1: 36 and 243 markers; FvbPat_4: 437 and 47 markers). At LOD 4, the MRD30 markers yielded seven linkage groups (FvbMat_1−FvbMat_7), matching FvbPat_1−FvbPat_7. Because we expected seven linkage groups in both parents, we retained the linkage groups generated at a LOD of 3 for MRD60 and at a LOD of 4 for MRD30. Despite not being specifically targeted, we observed informative polymorphisms on scA and scB mapping both to FvbPat_4 (4 polymorphisms) and FvbMat_4 (4 polymorphisms) (Table 1, Figure S2, D and K). We could order 2917 polymorphisms (37%) unambiguously within linkage groups at a LOD of 2; other polymorphisms were placed in the most likely position based on LOD score and confirmed by eye.

**Table 1:** Linkage groups observed

**Linkage Group***^a^*	**Main FvH4 Pseudochromosome***^b^*	**Markers***^c^*	**Size**, **cM***^d^*	**Size**, **Mb***^e^*	**Map Locations Observed***^f^*	**Other FvH4 Pseudochromosomes***^g^*
FvbPat1	1	279	60.4	22.5	17	0 (53), 6 (32), 7 (1)
FvbPat2	2	649	52.1	31.7	20	0 (109), 3 (1), 5 (19), 6 (13), 7 (1) 506
FvbPat3	3	223	50.0	28.1	19	1 (2), 4 (1), 5 (3)
FvbPat4	4	484	54.2	32.5	17	scA (1), scB (3), 0 (5), 2 (3), 3 (3), 5 (16), 6 (2)
FvbPat5	5	667	60.4	29.4	27	0 (2), 1 (35), 3 (9), 4 (19), 6 (6)
FvbPat6	6	1049	77.1	39.0	26	1 (60), 3 (12), 4 (33), 7 (2)
FvbPat7	7	590	52.1	24.3	23	3 (32), 6 (4)
FvbMat1	1	507	47.9	22.5	22	0 (58), 5 (22), 6 (46)
FvbMat2	2	776	54.2	31.7	24	0 (111), 3 (29), 5 (1), 6 (26), 7 (1)
FvbMat3	3	358	62.5	28.1	27	5 (4), 7 (20)
FvbMat4	4	649	58.3	32.5	18	scA (1), scB (3), 0 (16), 1 (2), 2 (2), 3 (2), 5 (39), 6 (5)
FvbMat5	5	508	62.5	29.4	25	0 (1), 4 (27), 7 (2)
FvbMat6	6	940	62.5	39.0	27	1 (56), 3 (13), 4 (29), 5 (1), 7 (4)
FvbMat7	7	600	62.5	24.3	26	3 (54), 4 (1), 6 (7)

a Linkage group in *F v*. ssp. *bracteata*. Groups beginning with “FvbPat” are paternal, and those with “FvbMat” are maternal.

b Pseudochromosome in FvH4 that shares the most markers with each linkage group.

c Number of polymorphic markers in the linkage group.

d Size of linkage group in centimorgans.

e Size of linkage group in megabases.

f Number of map locations at which at least one marker was directly observed.

g Other FvH4 pseudochromosomes with markers on this linkage group. Number of markers listed in parentheses.

We inferred 392 recombination events between both parents based on 318 mapped locations represented by at least one marker, indicating a genome-wide mean recombination rate of 1.0 cM/Mb (Table 1). Relative to FvH4, we observed 22 inversions, 11 intrachromosome translocations, and 40 interchromosome translocations (shown as curved lines across the middle of the circle in Figure 2, Table S3). We were also able to assign 14 scaffolds from the unmapped FvH4_0 to locations on the Fvb linkage map (Table S3). Of these 87 incongruities between FvH4 and Fvb, 91% were supported by more than one polymorphic marker (median = 18 polymorphisms), suggesting they are unlikely to be mapping errors in Fvb, although some could represent assembly errors in FvH4. Linkage maps developed independently from both parents were very similar, such that inversions and translocations suggested by one map were compatible with the other map, and recombination rates across the genome were correlated between parents (r_s_ = 0.48; *P* < 10^−13^; Figure S3). Most (60%) genomic regions showed no recombination in either parent, and those showing recombination often showed it in both parents. Recombination rate varied across the genome, ranging from an observed rate of zero across 23Mb in the central portion of FvbPat_6/FvbMat_6, to more than 20 cM/Mb near the end of FvbPat_7/FvbMat_7. Recombination rate was positively correlated with gene density in the reference genome (r_s_ = 0.52; *P* < 10^−15^).

### Male sterility mapping

**Figure 3:**
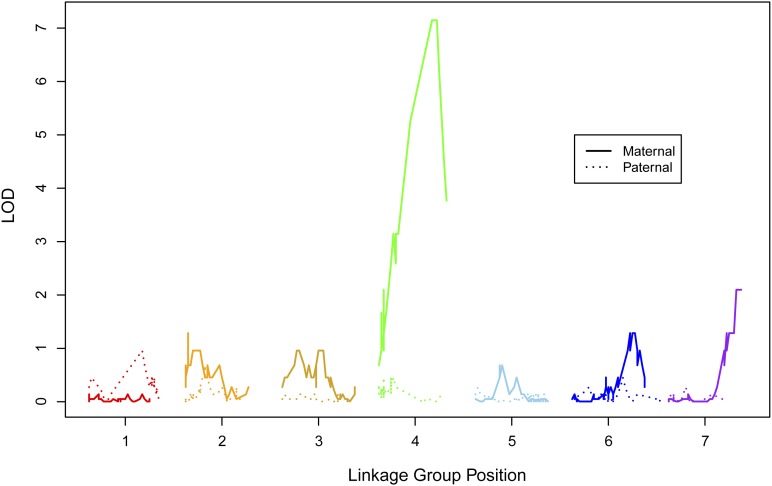
Probability of the sex locus mapping across the genome. A LOD score greater than 3 occurs in one genomic location: near the end of the maternal linkage group 4 (FvbMat_4) (LOD = 7.1 for markers at 22−24 cM, corresponding to markers mapping from 26.6−26.8Mb on FvH4_4). Markers on scB not shown.

**Figure 4:**
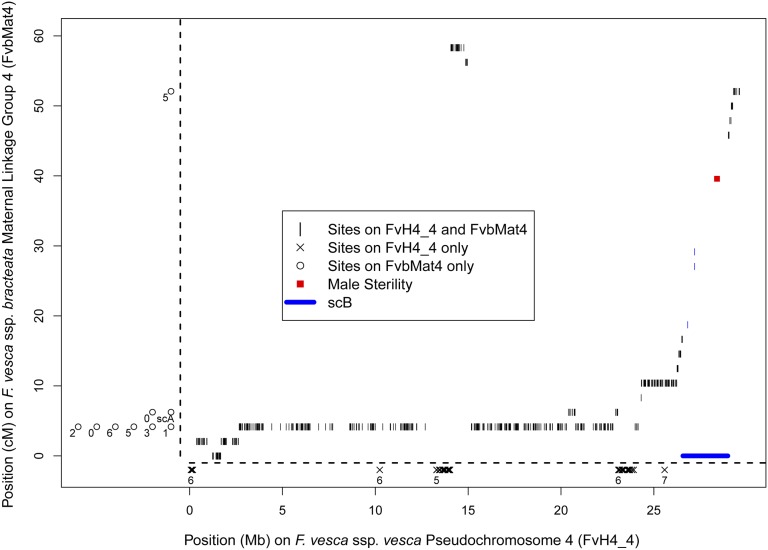
Maternal linkage map FvbMat_4 aligned to pseudochromosome 4 of the *F. vesca* ssp. *vesca* reference genome (FvH4_4). Each short vertical line represents an informative marker in our linkage map. The male-sterility locus resides within the red square that maps to the scaffold scB (thick blue line) which has here been inserted into its inferred location in FvH4_4. There are few informative targeted capture markers on scB (blue vertical lines) because it was not included in probe design. Markers that aligned somewhere in the reference genome other than FvH4_4 are indicated as circles along left side of the figure, numbered according to FvH4 pseudochromosome. Markers that aligned to FvH4_4 but mapped to a different linkage group in Fvb are indicated as x marks along the bottom of the figure, numbered according to FvbMat linkage group. Markers that map to one part of FvbMat_4 but align to a different part of FvH4_4 are within-chromosome translocations, such as the markers at 56−58 cM on FvbMat_4 occurring between 14-15Mb on FvH4_4.

In all 95 progeny that flowered, 49 were hermaphrodite and 46 were female; thus, male function segregated in a 1:1 ratio (*χ****^2^*** = 0.09; *P* = 0.75). Of the 48 offspring used in targeted capture linkage mapping, 40 flowered including 22 hermaphrodites and 18 females. Thus, male function also segregated in a 1:1 ratio in this subset (*χ****^2^*** = 0.4; *P* = 0.52). Using these 40 targeted capture plants, we unambiguously mapped male sterility to a region near the end of FvbMat_4 (Figure 3, Figure 4). The two closest markers on FvH4 were FvH4_4_26535351 upstream, at which eight individuals mismatched sex (LOD = 3.1), and FvH4_4_26597498 downstream, at which three different individuals mismatched sex (LOD = 7.1). The scaffold scB also mapped in between these same two markers. Marker scB_629161 was the closest match upstream with only three mismatches to sex (LOD = 7.1), and thus we inferred the male-sterility locus to occur between scB_629161 and FvH4_4_26597498. No targeted capture marker showed a perfect match to sex.

**Figure 5:**
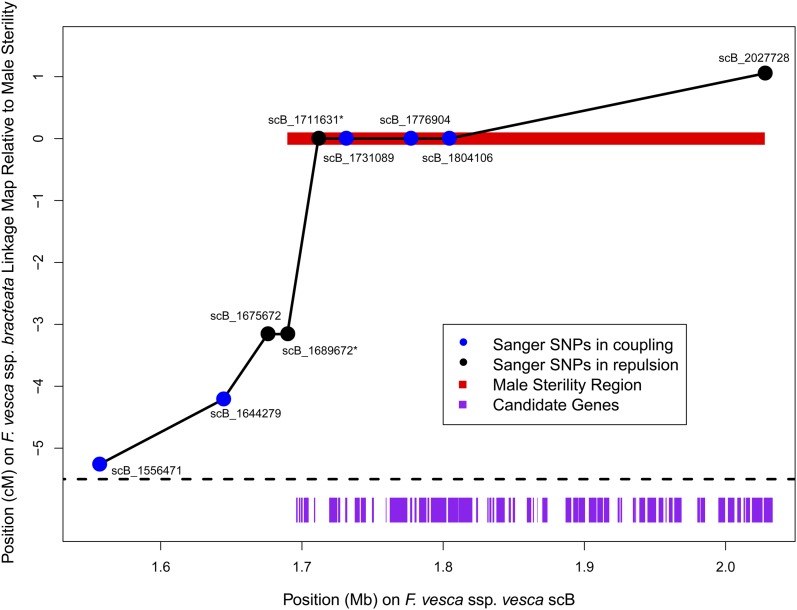
Fine-mapping of the candidate region with Sanger sequencing. Each informative SNP (circle) is plotted according to its position on scB (x-axis) and its mapped position based on observed recombination among 95 offspring (y-axis). SNPs are colored according to whether they are in coupling (*i.e.*, cis; blue) or in repulsion (*i.e.*, trans; black) with the inferred male-sterility locus. Most SNPs were genotyped in the full sample of offspring (n = 93−95), but two SNPs (marked with asterisks) were only genotyped in a subset of individuals including all potential recombinants as inferred from the other SNPs (n = 13). The 338-kb region consistent with a perfect match to sex is shown in red, and genes in this region are indicated with purple rectangles (Table 2).

We inferred 14 recombination events on scB in the 48 targeted capture plants, suggesting a recombination rate across scB of 11.8 cM/Mb, and allowing us to conduct additional fine-mapping. To do so we identified nine informative polymorphisms (Figure 5) on scB based on the whole genome sequencing of the parents and genotyped them in the parents and progeny via Sanger sequencing. We genotyped seven of these polymorphisms in all 95 progeny with zero to two missing genotypes per site, and we genotyped two polymorphisms in smaller subsets of progeny that included all potentially recombinant individuals based on the other polymorphisms. Genotypes at four of these polymorphisms showed a perfect match to sex, including three in coupling with male sterility (LOD = 28.6). The polymorphisms on either side of these perfect matches (scB_1689672 with three mismatches, and scB_2027728 with one mismatch) define a maximum candidate region of 338kb containing 57 genes based on FvH4 (Table 2). These genes represent a diverse array of functional roles, but several are known to be expressed in pollen or essential for pollen development, at least in *Arabidopsis* (Table 2).

**Table 2:** List of 57 genes in the 338kb genomic region on scB (sites 1689672−2027728) that shows a perfect match to sex

**Gene**	**Description**	**Start**	**End**
04131*^a^*	Cytochrome b561 (probable)	1695908	1696955
04132*^a^*	Cytochrome b561 (probable)	1697882	1698854
04133*^a^*	Protein pelota (probable)	1699354	1700487
04134*^a^*	Protein VERNALIZATION INSENSITIVE 3 (probable)	1701336	1704731
04135	Zinc finger protein 4 (probable)	1708545	1709366
04136*^a^*	Signal transduction histidine-protein kinase barA (probable)	1719309	1725022
04137*^a^*	Conserved oligomeric Golgi complex subunit 8 (COG complex subunit 8) (probable)	1725631	1727100
04138	F-box/kelch-repeat protein At3g06240 (probable)	1730662	1732093
04139*^a^*	Probable receptor protein kinase TMK1, Precursor (putative)	1737410	1740919
04140*^a^*	Probable receptor protein kinase TMK1, Precursor (putative)	1741738	1745245
04141*^a^*	U-box domain-containing protein 21 (probable)	1749596	1750933
04142	Zonadhesin, Precursor (probable)	1759358	1759831
04143*^a^*	THO complex subunit 2 (Tho2) (probable)	1762188	1774622
04144	Putative FBD-associated F-box protein At5g56690 (probable)	1776627	1778060
04145*^a^*	Probable clathrin assembly protein At4g32285	1779727	1781103
04146*^b^*	Putative receptor protein kinase ZmPK1, Precursor (probable)	1782940	1788099
04147	Dihydroorotase (DHOase) (probable)	1788929	1790128
04148*^a^*	Transmembrane anterior posterior transformation protein 1 homolog (probable)	1791303	1802303
04149	Cyclin-SDS (probable)	1803461	1810613
04150*^a^*	Probable leucine-rich repeat receptor-like protein kinase At1g35710, Precursor	1810803	1820658
04151*^a^*	PPPDE peptidase domain-containing protein 2 (probable)	1823385	1824658
04152*^a^*	Uncharacterized protein At4g28440 (similar to)	1831297	1832134
04153*^b^*	Putative F-box/LRR-repeat protein At5g41840 (probable)	1832760	1834112
04154	Glycine/sarcosine/betaine reductase complex component C subunit alpha (Protein PC alpha) (probable)	1834998	1836143
04155*^a^*	Ubiquitin carboxyl-terminal hydrolase isozyme L5 (UCH-L5) (probable)	1837700	1843573
04156	Protein CCC1 (probable)	1846416	1847932
04157	Protein CCC1 (probable)	1849284	1850869
04158	Heat stress transcription factor A-8 (AtHsfA8) (similar to)	1859247	1862358
04159	Homeobox protein rough sheath 1 (probable)	1863517	1864254
04160	Serine/arginine repetitive matrix protein 1 (probable)	1866464	1866799
04161*^a^*	Alpha,alpha-trehalose-phosphate synthase [UDP-forming] 6 (AtTPS6) (putative)	1870217	1873846
06233	Ribonuclease R (RNase R) (probable)	1886708	1890718
06234	Probable S-adenosylmethionine-dependent methyltransferase At5g38100	1892129	1895522
06235*^a^*	Probable RNA-dependent RNA polymerase 1 (OsRDR1) (similar to)	1895829	1900439
06236*^a^*	tRNA nucleotidyltransferase, mitochondrial, Precursor (probable)	1903160	1908555
06237*^a^*	Probable RNA-dependent RNA polymerase 1 (OsRDR1) (similar to)	1909159	1913119
06238*^a^*	Probable RNA-dependent RNA polymerase 1 (OsRDR1) (similar to)	1913797	1917596
06239	Probable RNA-dependent RNA polymerase 2 (OsRDR2)	1923582	1924947
06240	Nucleoside diphosphate-linked moiety X motif 17 (Nudix motif 17) (probable)	1925581	1926702
06241*^a^*	Pre-mRNA-splicing factor cwc22 (probable)	1934341	1935780
06242	Shikimate kinase (SK) (probable)	1936002	1936594
06243*^a^*	Probable RNA-dependent RNA polymerase 1 (OsRDR1) (similar to)	1938940	1942992
06244*^a^*	Aspartate-semialdehyde dehydrogenase (ASA dehydrogenase) (probable)	1944681	1950781
06245	Two-component response regulator ARR14 (similar to)	1952583	1955894
06246	tRNA modification GTPase mnmE (probable)	1957499	1958195
06247*^a^*	Adagio protein 3 (FBX2a) (putative)	1959870	1962462
06248*^a^**^,^**^b^*	Dynamin-related protein 1C (putative)	1963652	1968778
06249	F-box protein At3g59000 (probable)	1979978	1981804
06250	hypothetical protein	1982266	1985447
06251	Myosin-7 (MyHC-beta) (probable)	1994893	1999664
06252	Integrator complex subunit 11 (Int11) (probable)	2001649	2006256
06253*^a^*	E3 ubiquitin-protein ligase At4g11680 (probable)	2008307	2010848
06254	cGMP-gated cation channel alpha-1 (CNG-1) (probable)	2012734	2013688
06255	Farnesyltranstransferase, Precursor (similar to)	2014248	2014574
06256*^a^*	UDP-3-*O*-[3-hydroxymyristoyl] *N*-acetylglucosamine deacetylase, Precursor (putative)	2014656	2017275
06257*^a^*	Nuclear pore complex protein Nup155 (probable)	2018622	2026133
06258	Protein VAC14 homolog (probable)	2027060	2033300

a Genes with Plant Ontology categorization under “pollen” based on expression data in *Arabidopsis* (Schmid *et al.* 2005).

b Genes with putative functional roles in pollen development or recognition, highlighted in the *Discussion* section.

## Discussion

In the process of mapping male sterility in *F. v*. ssp. *bracteata*, we have generated a high-density linkage map which we can compare to the *F. vesca* reference genome. One striking result from this comparison is the large number of well-supported translocations and inversions (Figure 2, Table S3). We cannot determine from our data whether these are real evolutionary differences, errors in the genome assembly, or rearrangements that resulted from the hybrid nature of the cross on which the FvH4 pseudochromosome assembly is based (*F. vesca* × *F. bucharica*). Some rearrangements correspond to a complete single scaffold, consistent with assembly error, while others do not (Table S3), so it is likely that our observations include both assembly errors and real structural rearrangements. Observed recombination rate varied across the genome (Figure 2, Figure S2) and was correlated with gene density as is common in plants (Schnable *et al.* 1998; Li *et al.* 2007) and other taxa (Nam and Ellegren 2012). Because of potential chromosomal rearrangements between FvH4 and Fvb, the observed recombination rate is likely influenced by both the true rate of crossing over and differences in genome architecture between the subspecies. Most chromosomes contained a wide region (~10 Mb or more) showing no recombination (Figure S2), possibly the result of centromeres or similar recombination-suppressing chromatin structure, mirroring patterns seen in a previous *F. vesca* linkage map (Sargent *et al.* 2012). Although we endeavored to find markers spaced evenly across the genome, we were not able to generate many probes for the middle of FvH4_3, and those that we did use turned out to map to other Fvb linkage groups (Figure 2). Apparently the two parents happen to be homozygous for large haplotype blocks throughout this portion of the chromosome.

Our approach uses high-throughput genotyping of thousands of polymorphisms to map a Mendelian trait with a linkage cross, and thus leverages the combined strengths of both genomics and classical genetics. As with other recent applications of targeted sequence capture (Bi *et al.* 2012; Del Viso *et al.* 2012; Nadeau *et al.* 2012; Salmon *et al.* 2012; Tennessen *et al.* 2012), we have shown that MYcroarray MYbaits are highly effective in genotyping a large number of samples at thousands of sites. We have multiplexed 12 Illumina libraries in a single target enrichment (hybridization) reaction, and sequenced 48 target-enriched libraries in a single HiSeq lane, yielding high coverage sequence of nearly 1% of the genome for all individuals (Table S1). Few studies have used targeted capture for trait mapping (but see Del Viso *et al.* 2012), in contrast to other, more heavily used high-throughput technologies like RAD mapping and highly parallel SNP genotyping (*e.g.*, Bradley *et al.* 2011; Anderson *et al.* 2012). Our results indicate that a targeted capture approach should be successful in future studies of linkage and comparative analyses of other genome-wide patterns.

We have mapped male sterility in *F. v*. ssp. *bracteata* to a single genomic region which in *F. v*. ssp. *vesca* is approximately 338kb and harbors 57 genes (Figure 5, Table 2). In *Arabidopsis*, homologs of many of these genes are important in pollen development. For example, a dynamin-related GTPase similar to gene06248 is required for plasma membrane maintenance in maturing pollen (Kang *et al.* 2003; Backues *et al.* 2010), and an F-box/LRR-repeat protein similar to gene04153 is required for pollen mitosis II (Gusti *et al.* 2009). Many genes in this region are known to be expressed in *Arabidopsis* pollen, including PPPDE protein, RNA-dependent RNA polymerase 1, and Aspartate-semialdehyde dehydrogenase (Table 2; Schmid *et al.* 2005; Wang *et al.* 2008). Only a single gene in this region appears in a recent curated database of genes involved in plant male reproduction (Cui *et al.* 2013): gene04146, encoding a putative receptor protein kinase structurally similar to S-loci, and thus potentially involved in pollen recognition. Notably absent are gene families known to cause male sterility in other species, such as PPR proteins (Hanson and Bentolila 2004; Wang *et al.* 2006), peptidase M48 proteins (Matsuhira *et al.* 2012), or acyl-CoA synthetases (Wang and Li 2009).

Because male sterility is dominant, we do not expect the causal polymorphism to be a simple loss of function in a gene essential for pollen development. Rather, a single allele in heterozygous state must somehow actively suppress pollen maturation. Although there are several promising candidates, the causal gene cannot be conclusively determined with our current data. It is also possible that the causal gene or its genomic location is unique to *F. v*. ssp. *bracteata*, such that it is not identifiable from the genes in this region of the reference genome. Additional experimental evidence to pinpoint the causal gene could include further fine-mapping with larger sample sizes, genetic knockdown/knockout experiments, genetic expression data for anthers or pollen, and/or complete sequencing of the candidate region in *F. v*. ssp. *bracteata*.

Male sterility is dominant, as seen in other *Fragaria* species, but its chromosomal location is not syntenic with the sex-determining regions of octoploid *Fragaria* (Goldberg *et al.* 2010; Spigler *et al.* 2011; Govindarajulu *et al.* 2013). There are three possible explanations for these observations. The first explanation is that the gene has been translocated at least twice among *F. v*. ssp. *bracteata*, *F. virginiana*, and *F. chiloensis*. Although some observed “translocations” (Figure 2, Table S3) may be genome assembly artifacts, our results suggest a dynamic ongoing restructuring of *Fragaria* genomes and support the possibility that the male-sterility locus could have translocated. A double translocation of the same gene would be notable but consistent with a high rate of translocation for sex determining regions (Charlesworth and Mank 2010) and polyploid genomes (Lim *et al.* 2008; Gaeta and Pires 2010). The second explanation is that the relatively unusual trait of dominant male sterility has arisen multiple times independently in *Fragaria*. This interpretation would suggest that there are many possible biochemical mechanisms to disrupt pollen development (see Diggle *et al.* 2011, and references in introduction) in a dominant fashion. Perhaps some of these mechanisms are more likely to persist for long time periods, or under certain ecological contexts, and thus conducive to the eventual evolution of subdioecy and dioecy (Spigler and Ashman 2012), while others are more short-lived and are lost with a reversion to total hermaphroditism (Dufay and Billard 2012). Repeated independent evolution of dominant male sterility in *Fragaria* but not other taxa would suggest that some unique aspect of *Fragaria* genetics, evolutionary history or ecology favors these mutations. The third possibility is that male sterility evolved only once in *Fragaria*, such that the biochemical mechanism is the same in all three species, but the genetic control of this trait has shifted over time to other, unlinked genes that regulate the original gene encoding male sterility. This rapid turnover of the sex determination region may occur in taxa that do not have conserved sex chromosomes (van Doorn and Kirkpatrick 2007).

The recombination rate across scB (11.8 cM/Mb) is substantially greater than the genome-wide average (1.0 cM/Mb). The proportion of coding sites in scB (25.9%) is also greater than the genome-wide average (19.5%), although the recombination rate at scB is high even among gene-dense regions (mean = 6.0 cM/Mb for regions with >25% coding sites). We observed three recombination events in the relatively short (22 kb) genomic distance between scB_1689672 and scB_1711631, suggestive of a local recombination hotspot (144 cM/Mb; Figure 5). It is possible that the male-sterility locus was more likely to evolve in a high-recombination region because that is where relatively more genes are located. Alternatively, these results may indicate that there are large cryptic insertion(s) in scB in *F. v*. spp. *bracteata*, possibly containing the candidate gene, which would be consistent with dynamic chromosomal restructuring near sex-determining regions (Charlesworth and Mank 2010).

Male sterility in our *F. v*. spp. *bracteata* cross segregates in a Mendelian fashion and maps to the nuclear genome and thus does not conform to described features of CMS. Furthermore, several lines of evidence point away from the sterility locus being an Rf locus. First, male sterility in *F. v*. ssp. *bracteata* is dominant, which is not typical for Rf loci. The gene presumably performs some function which is lost in the recessive allele. If the function degrades CMS mRNAs, and a single copy of an allele is sufficient to perform the function, then heterozygotes should be fertile, as seen in other species (Hanson and Bentolila 2004; Wang *et al.* 2006). Therefore, it is more plausible that the biochemical role of the male-sterility locus is to directly suppress, not restore, pollen development. Second, although *F. v*. ssp. *bracteata* may differ somewhat from the reference genome, there is no evidence of PPR genes or other members of known Rf gene families in the genomic interval that shows a perfect match to sex. The evidence is more consistent with non-Rf nuclear determination of gynodioecy in this population of *F. v*. ssp. *bracteata* as described by Ahmadi and Bringhurst (1989) and suggests that ecological studies of fitness advantage need to consider whole life time components of quantity or quality or biotic interactions (Li *et al.* 2012; Dalton *et al.* 2013). Given the potential spatiotemporal heterogeneity of sex determination (Pannell 1997; Olson *et al.* 2006), however, our results do not preclude the possible role of CMS (or cyto-nuclear interactions) in other populations of *F. v*. ssp. *bracteata* or even in the recent evolutionary history of this population.

To complete our understanding of the evolution of sex determination in *Fragaria*, several additional steps will be necessary. Further fine-mapping and functional analyses are needed to identify the causal genes in *F. v*. ssp. *bracteata* and its congeners. In addition, studies with additional individuals and with *F. v*. ssp. *bracteata* from other populations will help evaluate the species-wide importance of the locus mapped in this study and determine if other independent loci are also involved in the control of male function. Our success with target capture in this study suggests that it will be feasible to generate high-density maps in other *Fragaria*, including polyploids, for comparative studies. Inferring the molecular evolutionary history of the causal genes for male sterility may reveal how and when natural selection has acted to shape sexual variation in this genus.

## Supplementary Material

Supporting Information
