# Supplementary material for: Targeted Sequence Capture Provides Insight into Genome Structure and Genetics of Male Sterility in a Gynodioecious Diploid Strawberry, Fragaria vesca ssp. bracteata (Rosaceae)
Source: G3 (Bethesda). 2013 Aug 1;3(8):1341–51. doi: 10.1534/g3.113.006288 (PMC3737174; doi:10.1534/g3.113.006288)
Supplement: Supporting Information [file supp_3_8_1341__index.html]

Targeted Sequence Capture Provides Insight into Genome Structure and Genetics of Male Sterility in a Gynodioecious Diploid Strawberry, Fragaria vesca ssp. bracteata (Rosaceae) — Supporting Information 

# Targeted Sequence Capture Provides Insight into Genome Structure and Genetics of Male Sterility in a Gynodioecious Diploid Strawberry, *Fragaria vesca* ssp. *bracteata* (Rosaceae)

## Supporting Information for Tennessen *et al.*, 2013

**Files in this Data Supplement:**

- Supporting Information - Figures S1-S3, Tables S1-S3, and Files S1-S3 (PDF, 2 MB)
- Figure S1 - Genotyping errors and missing data (PDF, 371 KB)
- Figure S2 - Linkage groups in *F. vesca* ssp. *bracteata* plotted against the *F. vesca* ssp. *vesca* reference genome (FvH4) (PDF, 1 MB)
- Figure S3 - Recombination rates are very similar in both parental maps (PDF, 283 KB)
- Table S1 - Targeted capture Illumina sequencing data for two parents and 48 offspring (PDF, 242 KB)
- Table S2 - Primer sequences and scaffold coordinates for nine informative polymorphic sites (PDF, 261 KB)
- Table S3 - Incongruities between FvH4 and Fvb (PDF, 288 KB)
- File S1 - FASTA file of the 6575 targeted regions (.zip, 445 KB)
- File S2 - Variant call format (vcf) file of all targeted capture genotypes used in linkage mapping (.zip, 2 MB)
- File S3 - Map position (Fvb, in cM) and reference genome position (FvH4, in bp) for all markers (.txt, 356 KB)
