# Supplementary material for: Targeted Sequence Capture Provides Insight into Genome Structure and Genetics of Male Sterility in a Gynodioecious Diploid Strawberry, Fragaria vesca ssp. bracteata (Rosaceae)
Source: G3 (Bethesda). 2013 Aug 1;3(8):1341–51. doi: 10.1534/g3.113.006288 (PMC3737174; doi:10.1534/g3.113.006288)
Supplement: Supporting Information [file supp_g3.113.006288_006288SI.pdf]

G3·Genes | Genomes | Genetics

Targeted sequence capture provides insight into genome structure and genetics of male sterility in a gynodioecious diploid strawberry, *Fragaria vesca* ssp. *bracteata* (Rosaceae)

Jacob A. Tennessen[*], Rajanikanth Govindarajulu[§], Aaron Liston[†] and Tia-Lynn Ashman[§,1]

*Department of Zoology and †Department of Botany and Plant Pathology, Oregon State University, Corvallis, OR 97331, §Department of Biological Sciences, University of Pittsburgh, Pittsburgh, PA 15260

[1]Corresponding author: University of Pittsburgh, Department of Biological Sciences, 215 Clapp Hall, 4249 Fifth Avenue, Pittsburgh, PA 15260. E-mail: tia1@pitt.edu

DOI: 10.1534/g3.113.006288

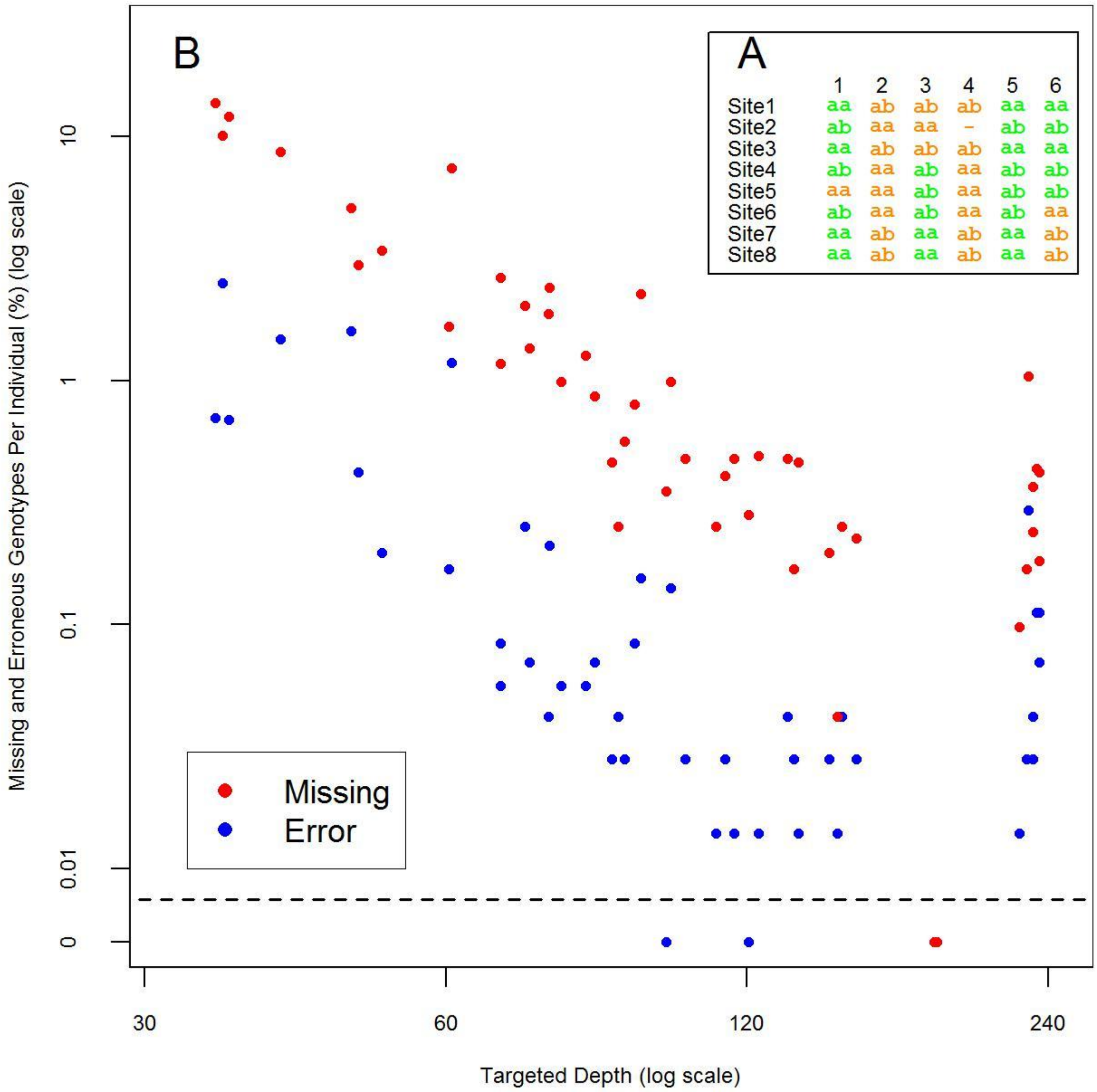

Figure S1 Genotyping errors and missing data. (A) Genotyping errors were identified as anomalies in the linkage map, as shown in this example genotype alignment. Heterozygotes are "ab", homozygotes are "aa", and missing data is "-". Rows are informative sites and columns are individual offspring. Individual 1 has an anomalous genotype at Site 5, in that it does not match what it expected based on the genotypes at Site 4 and Site 6. We would call such a genotype an error rather than a double recombination event. In contrast, the recombination event in Individual 6 that occurs between Site 5 and Site 6 appears to be real because it is supported by multiple sites. Individual 4 has missing data at Site 2 (did not pass quality filter based on Phred-scaled likelihood > 40 for alternate genotypes). (B)Scatterplot of missing data and genotyping errors per individual as a function of coverage (mean depth at targeted sites). We observed low per-individual rates of both missing data (mean = 2.1%; median = 0.6%) and genotyping errors (mean = 0.2%; median = 0.05%). Targeted depth was negatively correlated with both genotyping error (r_s = -0.60; $P < 10^{-05}$) and missing data (r_s = -0.85; $P < 10^{-14}$). For 8 offspring sequenced a separate lane, coverage was much higher (>200x) than for the remaining 40 offspring (Table S1).

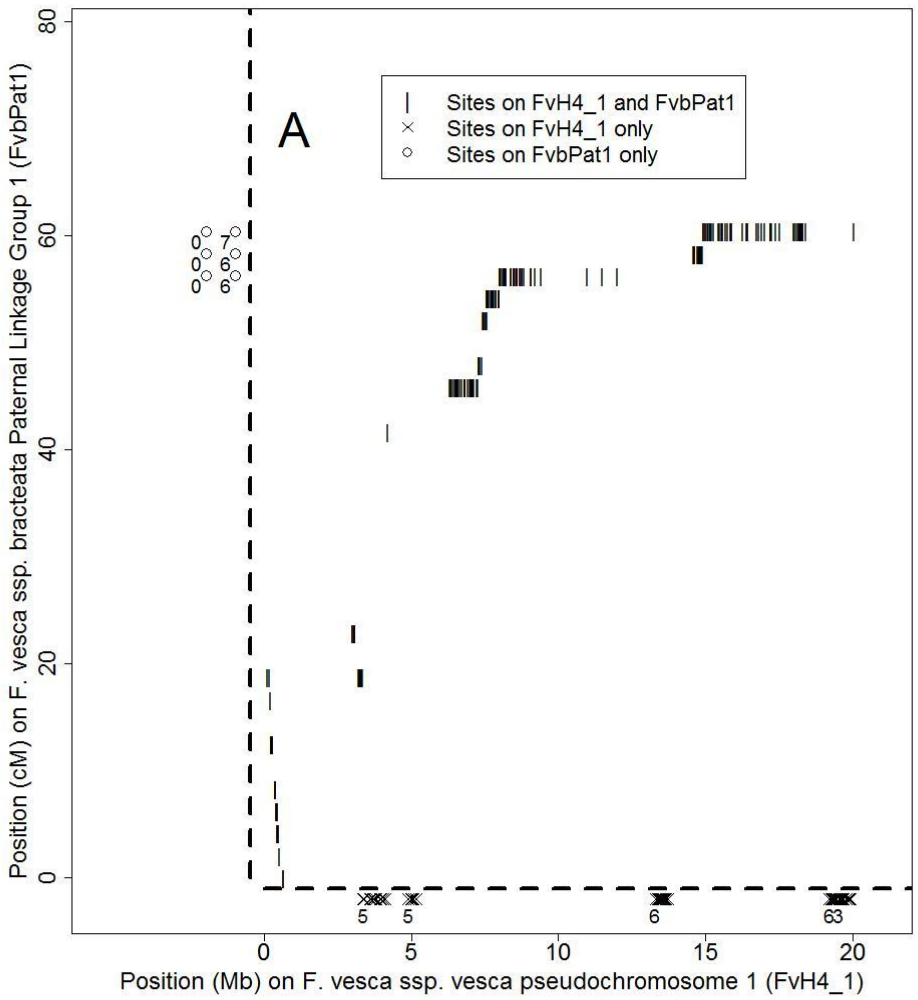
A
Sites on FvH4_1 and FvbPat1
Sites on FvH4_1 only
Sites on FvbPat1 only
Position (cM) on F. vesca ssp. bracteata Paternal Linkage Group 1 (FvbPat1)
Position (Mb) on F. vesca ssp. vesca pseudochromosome 1 (FvH4_1)
0
20
40
60
80
0
5
10
15
20

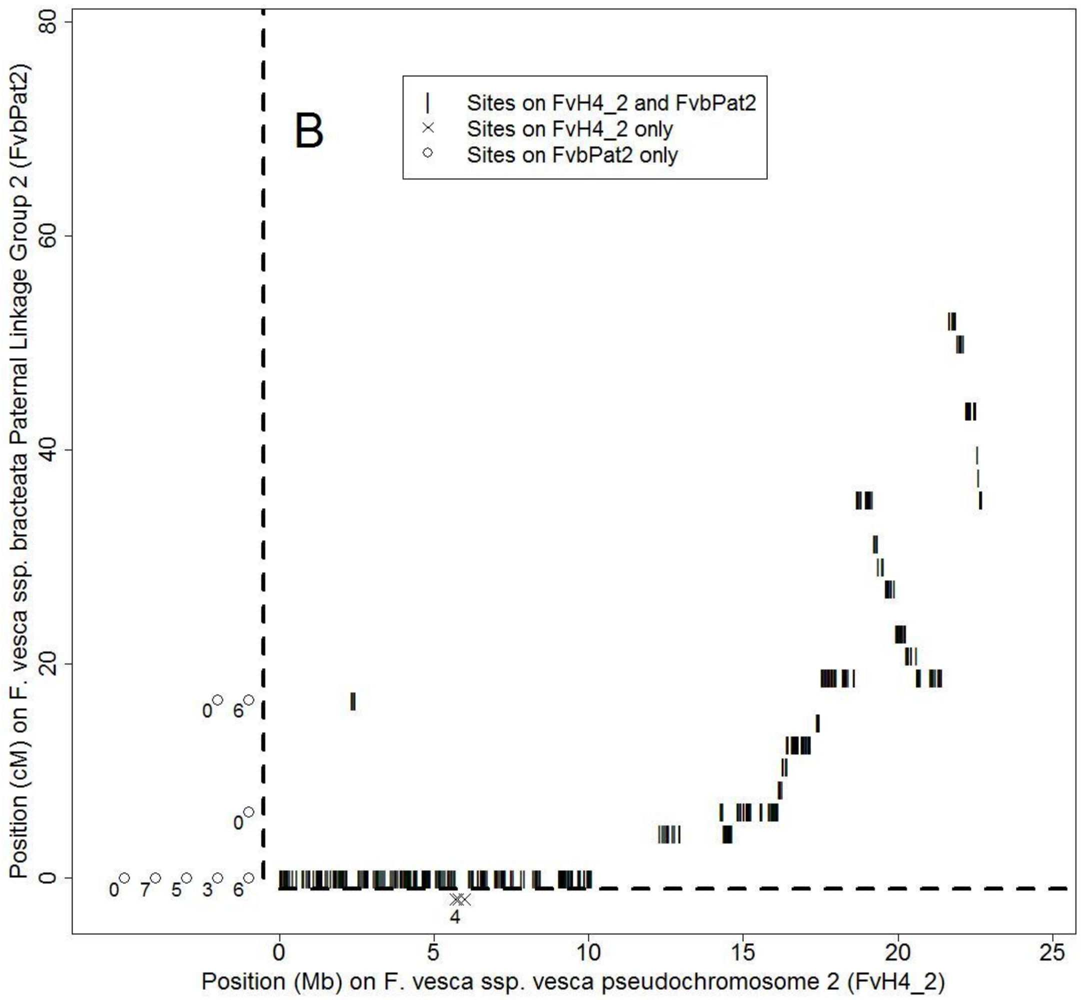
B
Sites on FvH4_2 and FvbPat2
Sites on FvH4_2 only
Sites on FvbPat2 only
Position (cM) on F. vesca ssp. bracteata Paternal Linkage Group 2 (FvbPat2)
Position (Mb) on F. vesca ssp. vesca pseudochromosome 2 (FvH4_2)
0
20
40
60
80
0
5
10
15
20
25
0 6
0
0 7 5 3 6
4

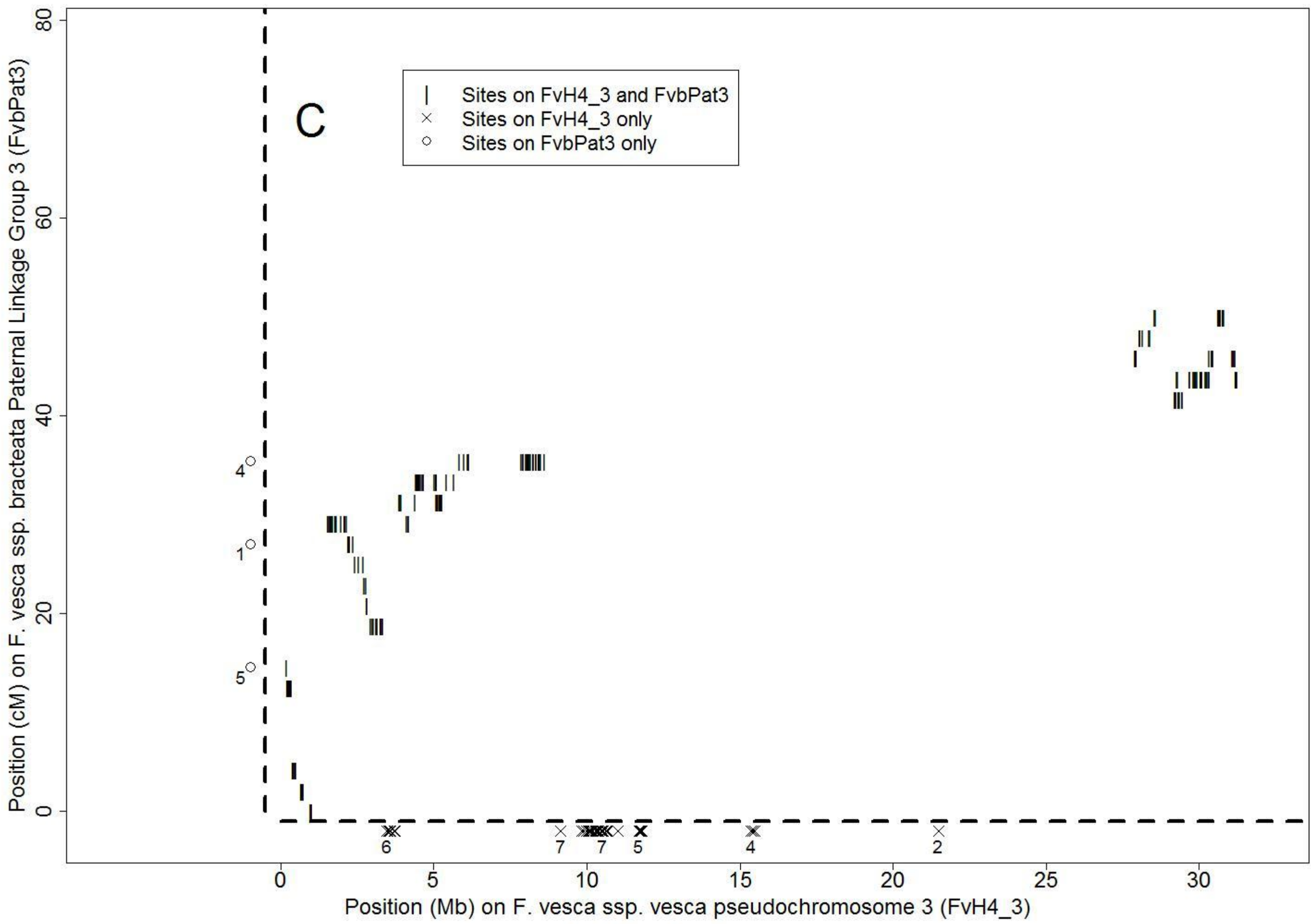
C
Sites on FvH4_3 and FvbPat3
Sites on FvH4_3 only
Sites on FvbPat3 only
Position (cM) on F. vesca ssp. bracteata Paternal Linkage Group 3 (FvbPat3)
Position (Mb) on F. vesca ssp. vesca pseudochromosome 3 (FvH4_3)
0
20
40
60
80
0
5
10
15
20
25
30
4
1
5
6
7
7
5
4
2

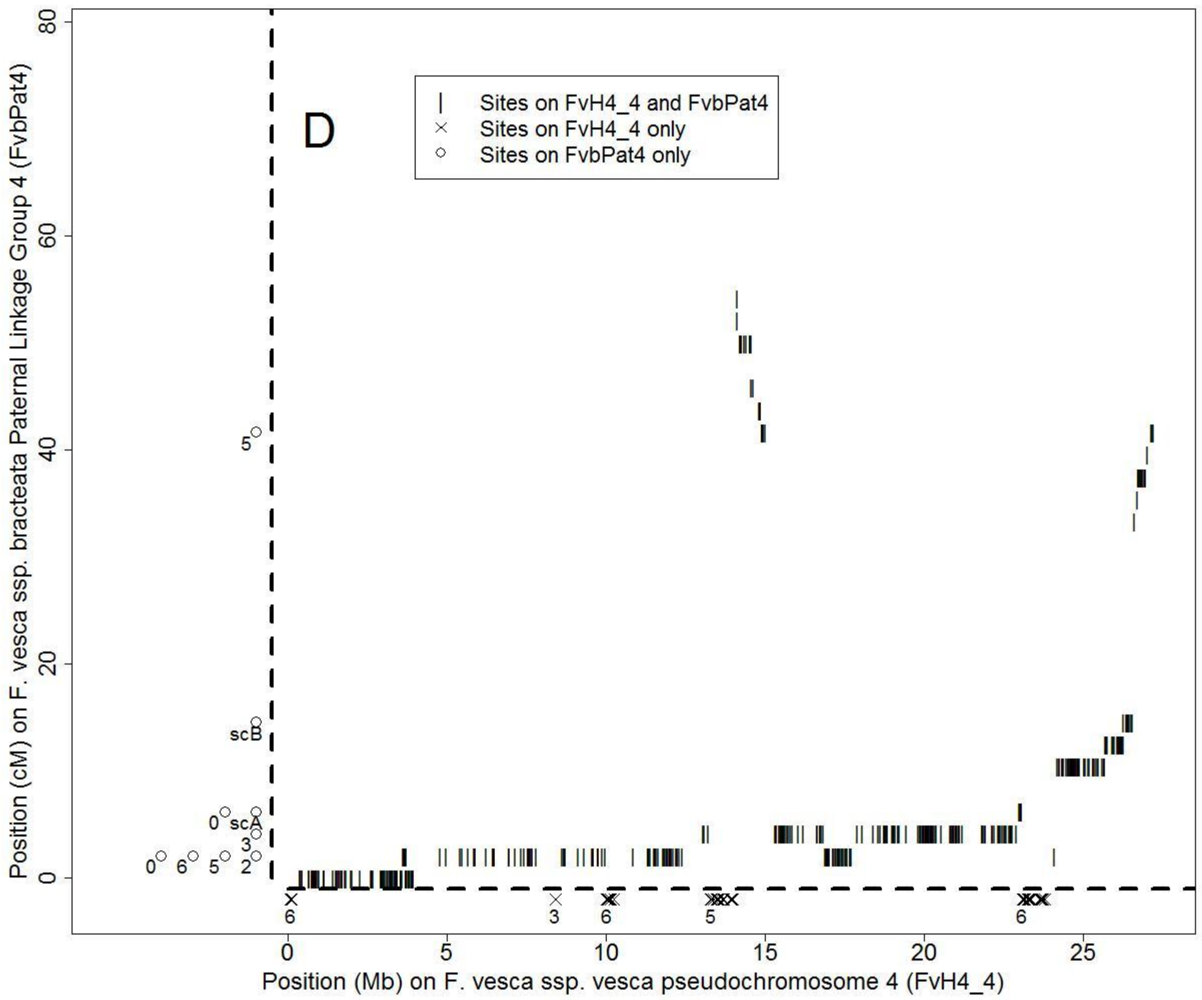

D
Sites on FvH4_4 and FvbPat4
Sites on FvH4_4 only
Sites on FvbPat4 only
Position (cM) on F. vesca ssp. bracteata Paternal Linkage Group 4 (FvbPat4)
Position (Mb) on F. vesca ssp. vesca pseudochromosome 4 (FvH4_4)
0
20
40
60
80
0
5
10
15
20
25
5
scB
0
scA
3
0
6
5
2
6
3
6
5
6

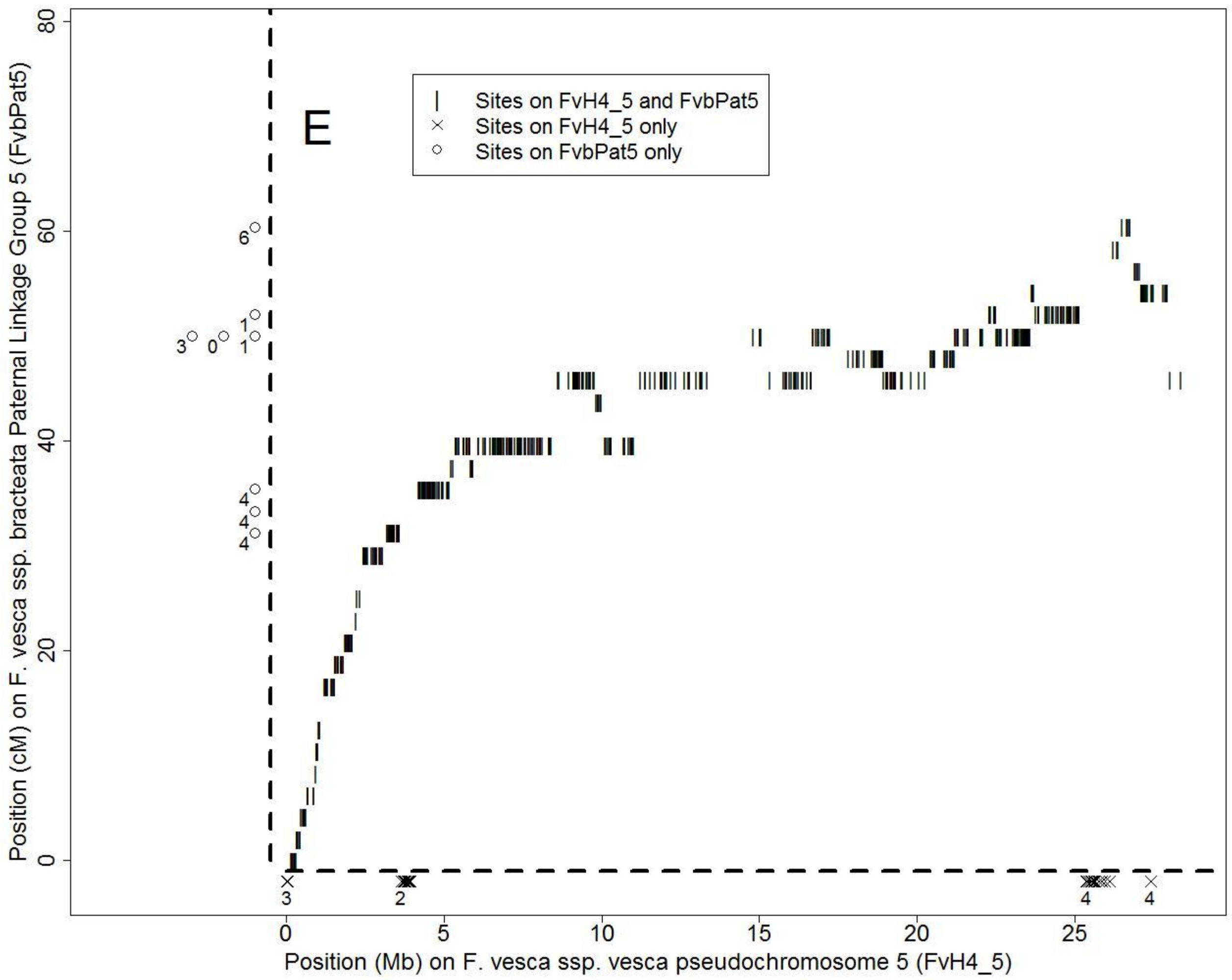
E
Sites on FvH4_5 and FvbPat5
Sites on FvH4_5 only
Sites on FvbPat5 only
Position (cM) on F. vesca ssp. bracteata Paternal Linkage Group 5 (FvbPat5)
Position (Mb) on F. vesca ssp. vesca pseudochromosome 5 (FvH4_5)
0
20
40
60
80
0
5
10
15
20
25
3
0
1
1
6
4
4
4
3
2
4
4

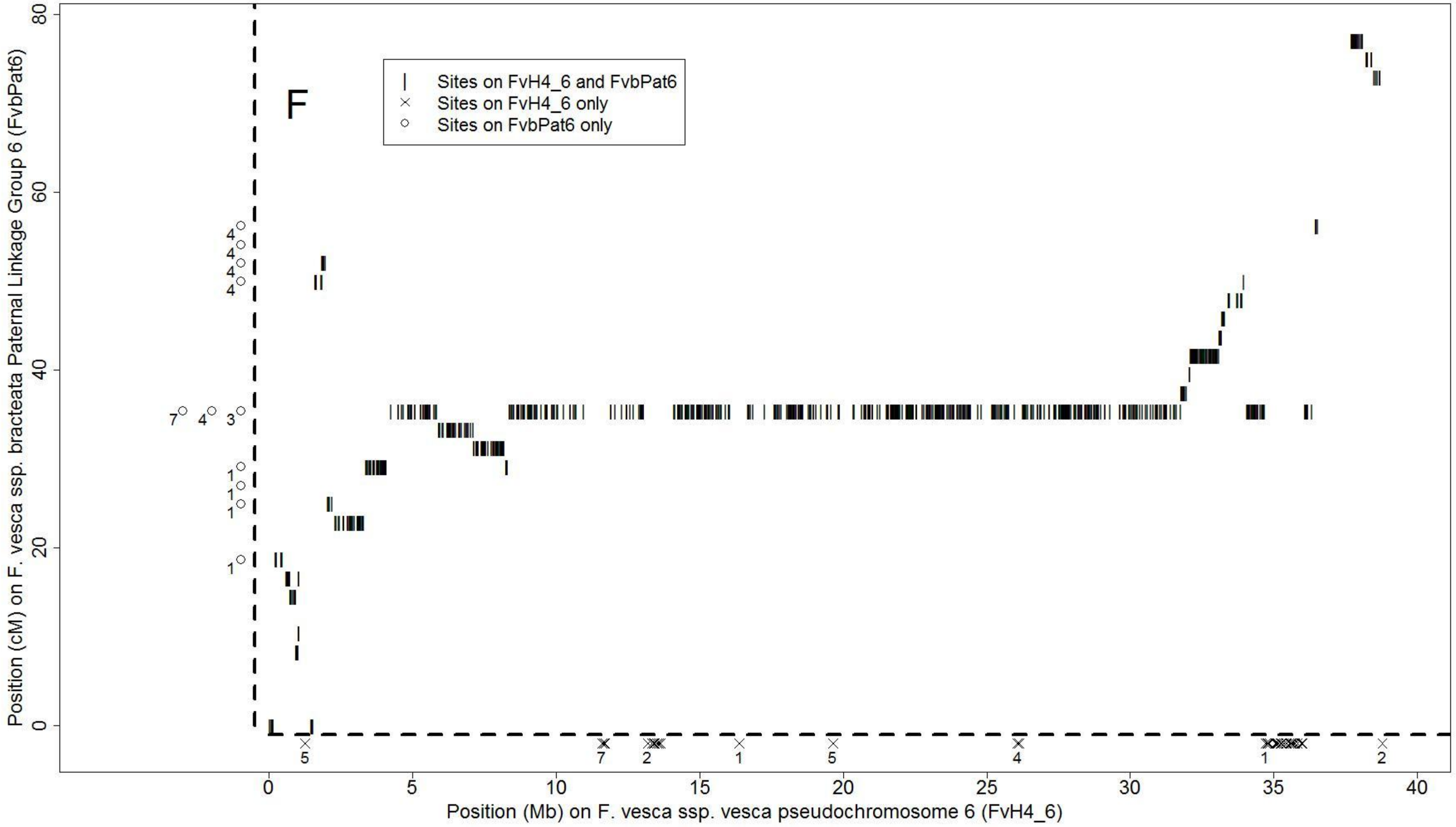
F
Sites on FvH4_6 and FvbPat6
Sites on FvH4_6 only
Sites on FvbPat6 only
Position (cM) on F. vesca ssp. bracteata Paternal Linkage Group 6 (FvbPat6)
Position (Mb) on F. vesca ssp. vesca pseudochromosome 6 (FvH4_6)

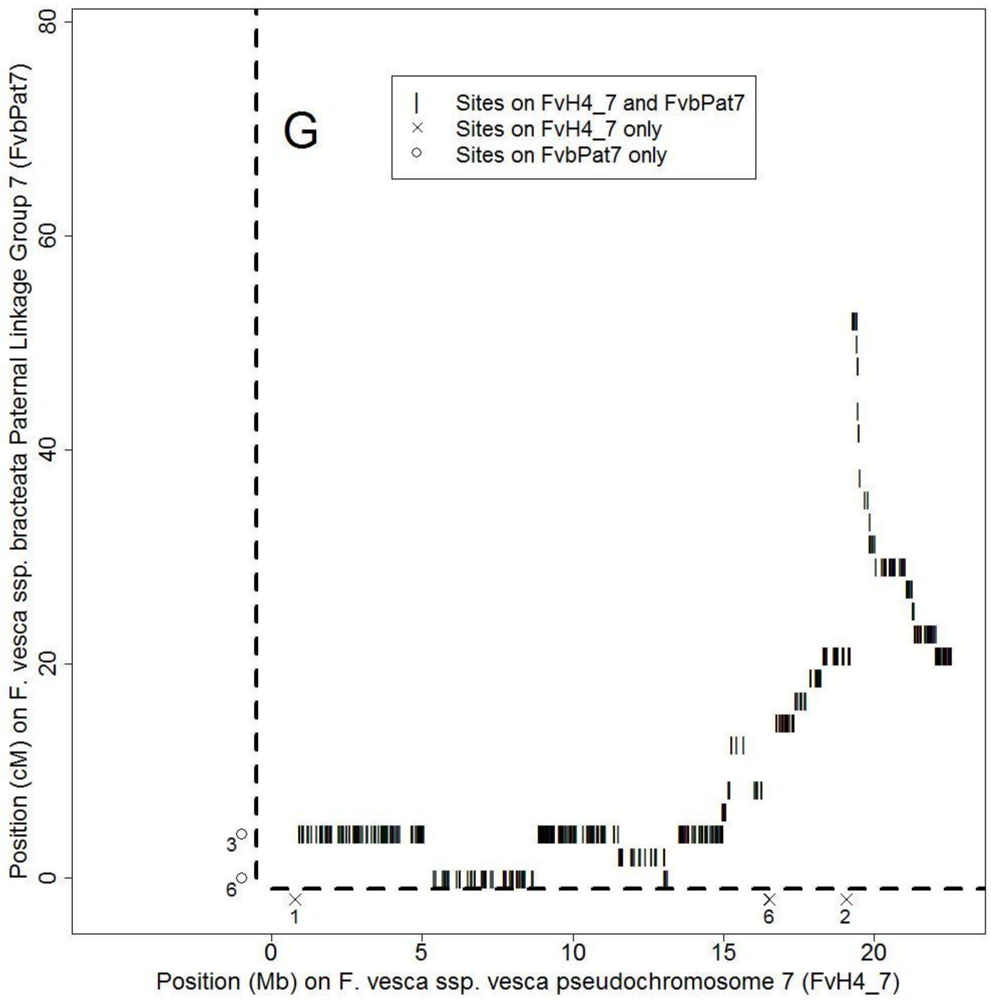
G
Sites on FvH4_7 and FvbPat7
Sites on FvH4_7 only
Sites on FvbPat7 only
Position (cM) on F. vesca ssp. bracteata Paternal Linkage Group 7 (FvbPat7)
Position (Mb) on F. vesca ssp. vesca pseudochromosome 7 (FvH4_7)
0
20
40
60
80
0
5
10
15
20
3
6
1
6
2

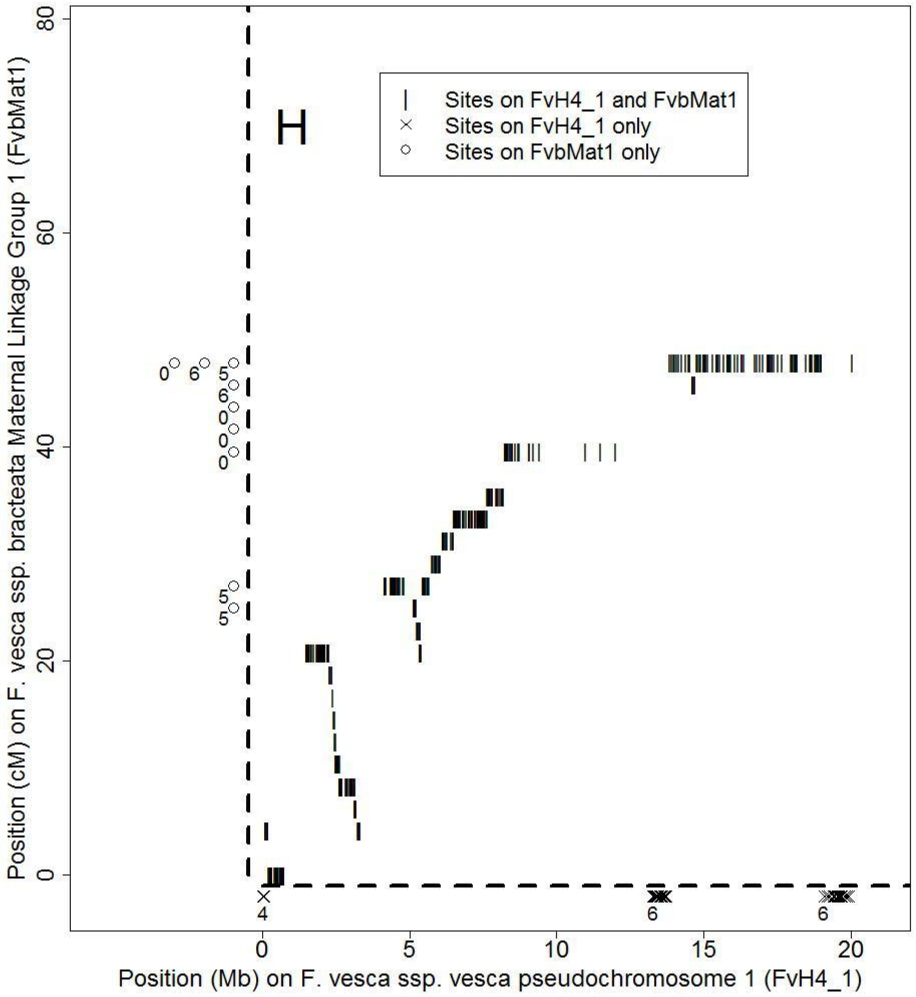
H
Sites on FvH4_1 and FvbMat1
Sites on FvH4_1 only
Sites on FvbMat1 only
Position (cM) on F. vesca ssp. bracteata Maternal Linkage Group 1 (FvbMat1)
Position (Mb) on F. vesca ssp. vesca pseudochromosome 1 (FvH4_1)
0
20
40
60
80
0
5
10
15
20

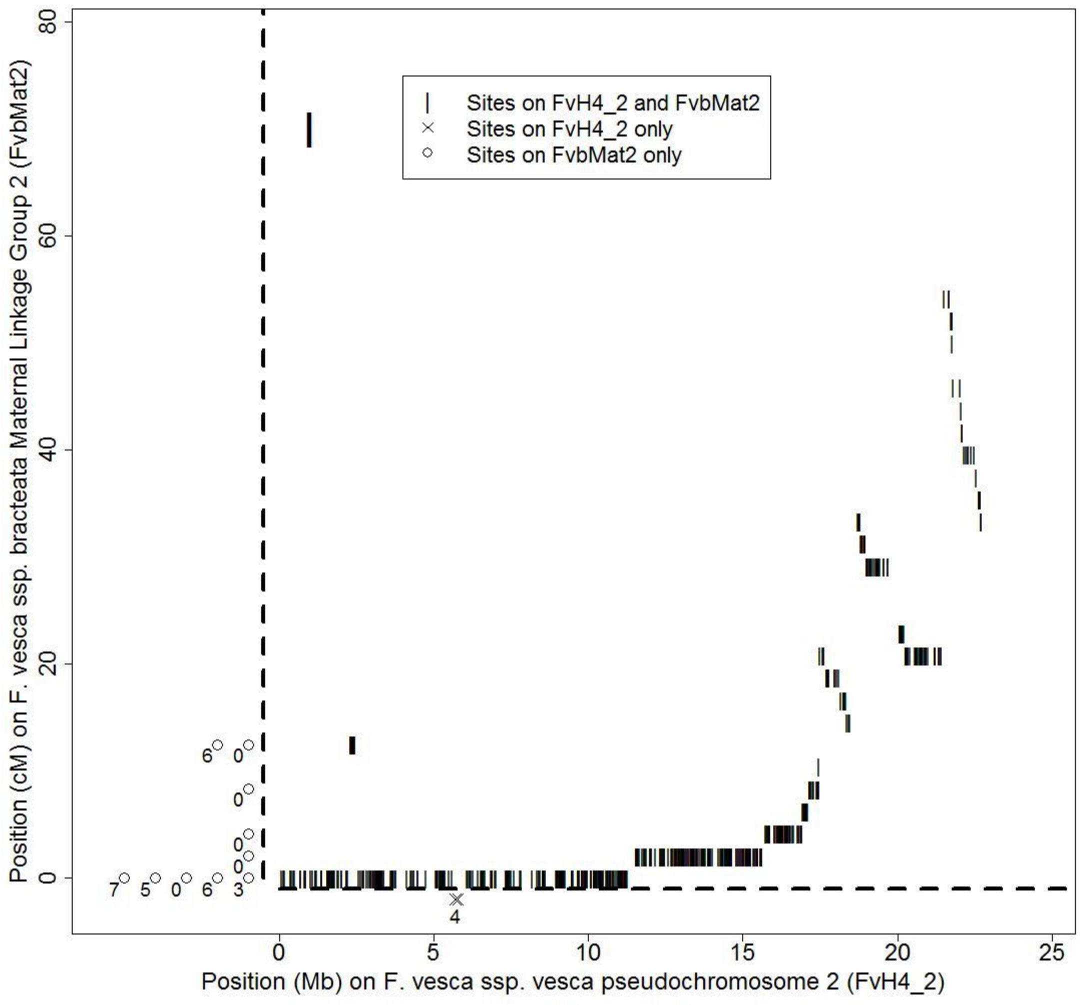
Sites on FvH4_2 and FvbMat2
Sites on FvH4_2 only
Sites on FvbMat2 only
Position (cM) on F. vesca ssp. bracteata Maternal Linkage Group 2 (FvbMat2)
Position (Mb) on F. vesca ssp. vesca pseudochromosome 2 (FvH4_2)
0
20
40
60
80
0
5
10
15
20
25
7
5
0
6
3
6
0
0
0
0
4

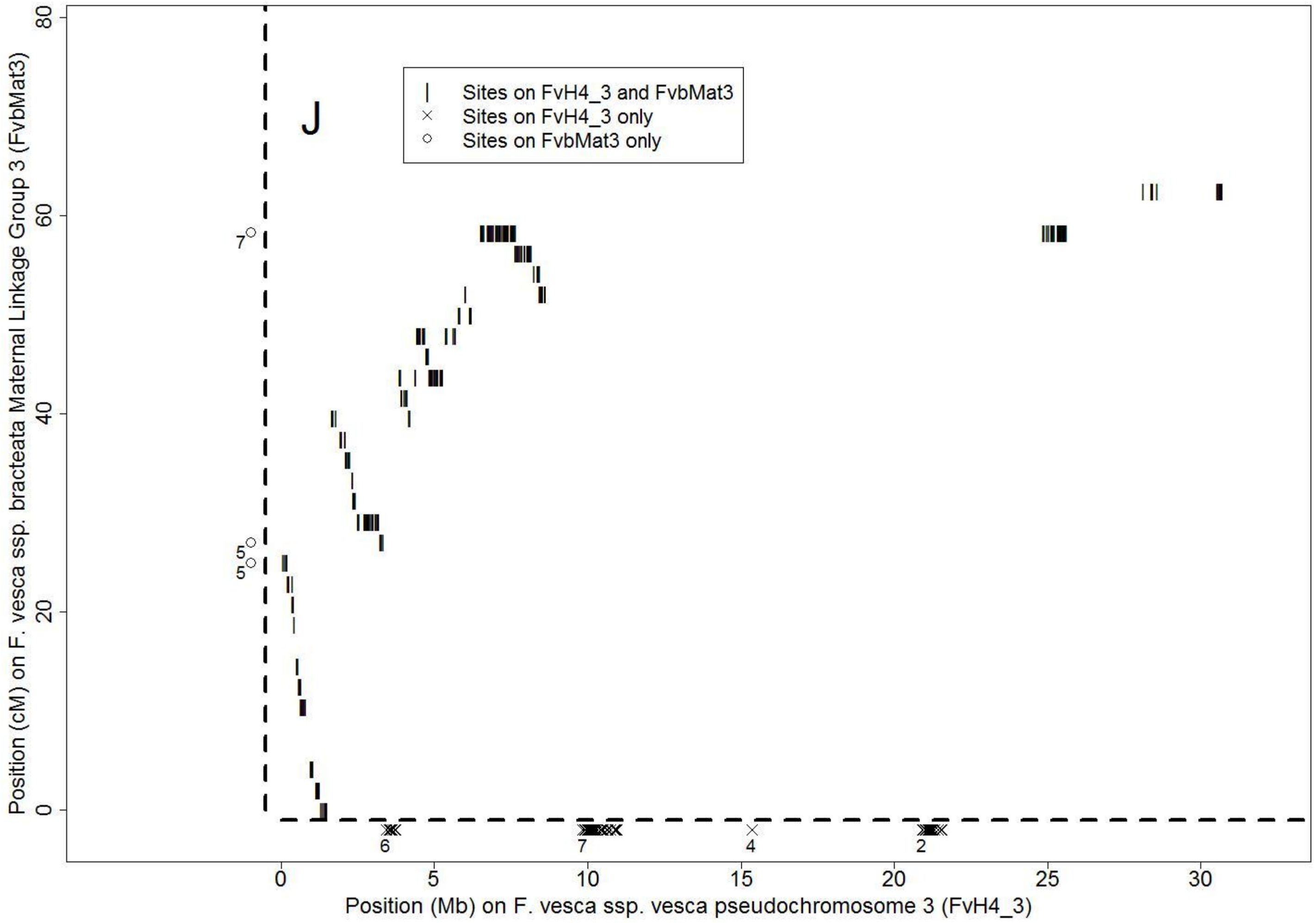
J
Sites on FvH4_3 and FvbMat3
Sites on FvH4_3 only
Sites on FvbMat3 only
Position (cM) on F. vesca ssp. bracteata Maternal Linkage Group 3 (FvbMat3)
Position (Mb) on F. vesca ssp. vesca pseudochromosome 3 (FvH4_3)
0
20
40
60
80
0
5
10
15
20
25
30
7
5
5
6
7
4
2

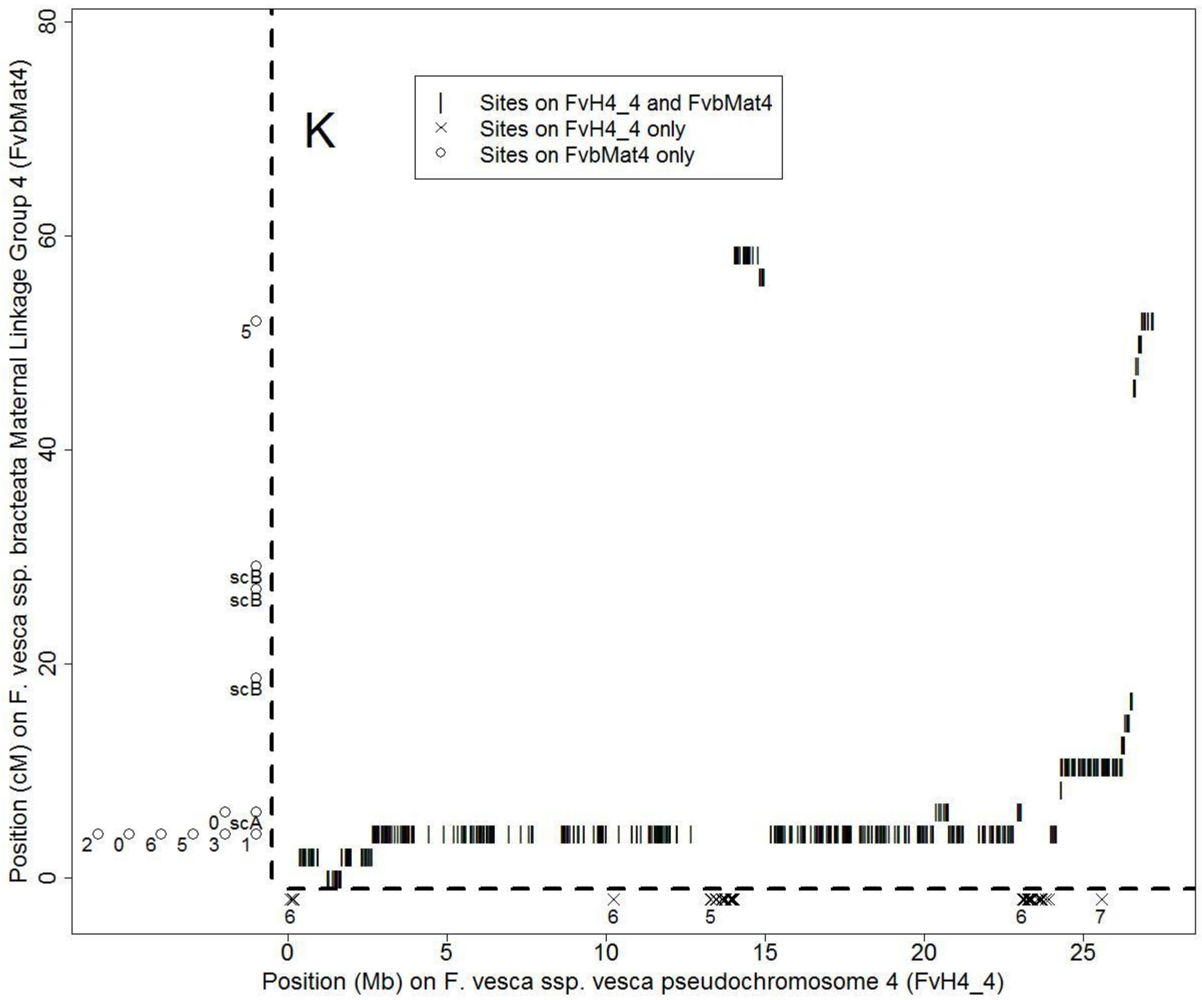

K
Sites on FvH4_4 and FvbMat4
Sites on FvH4_4 only
Sites on FvbMat4 only
Position (cM) on F. vesca ssp. bracteata Maternal Linkage Group 4 (FvbMat4)
Position (Mb) on F. vesca ssp. vesca pseudochromosome 4 (FvH4_4)
scB
scB
scB
scA

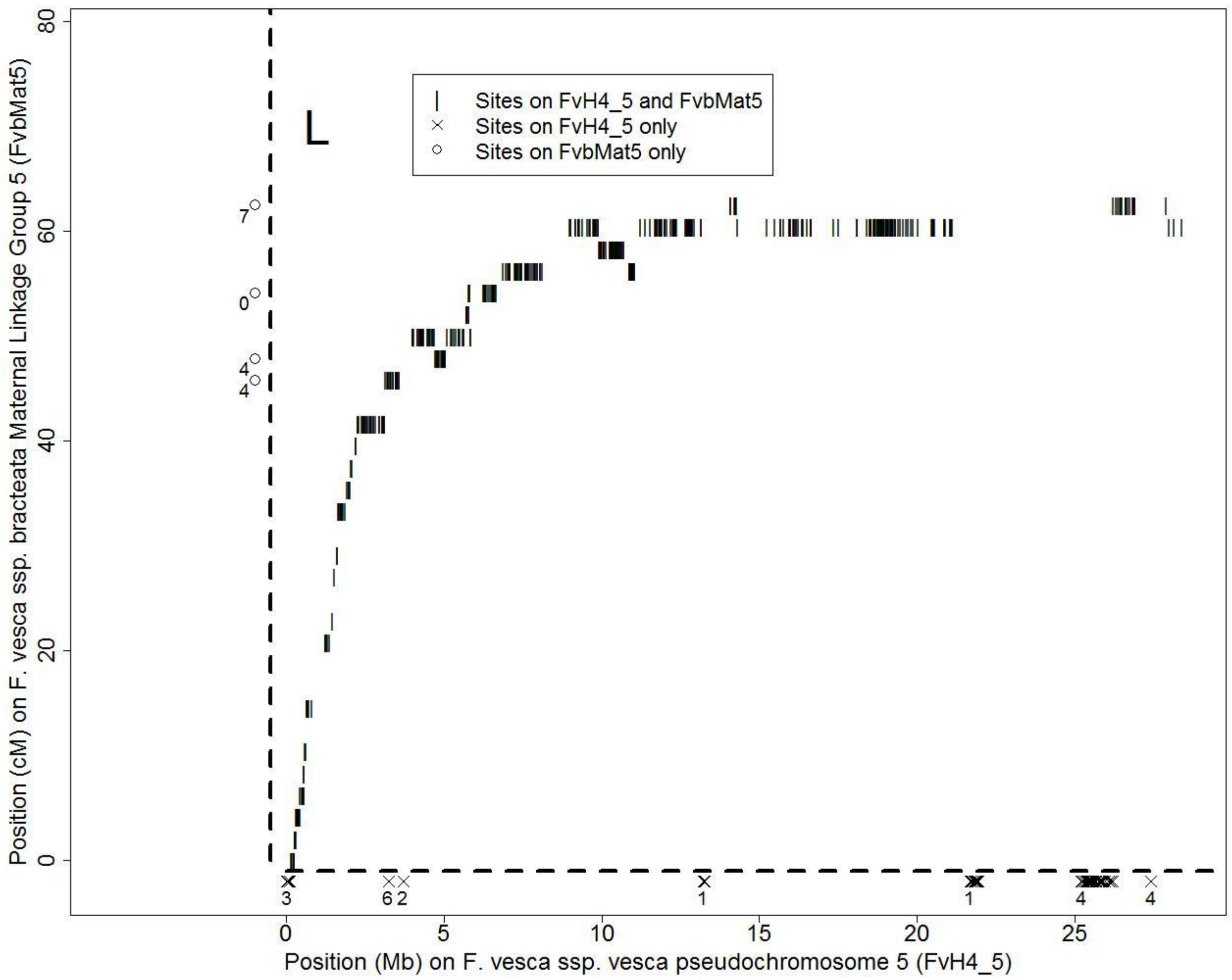
L
Sites on FvH4_5 and FvbMat5
Sites on FvH4_5 only
Sites on FvbMat5 only
Position (cM) on F. vesca ssp. bracteata Maternal Linkage Group 5 (FvbMat5)
Position (Mb) on F. vesca ssp. vesca pseudochromosome 5 (FvH4_5)
0
20
40
60
80
0
5
10
15
20
25
7
0
4
4
3
6 2
1
1
4
4

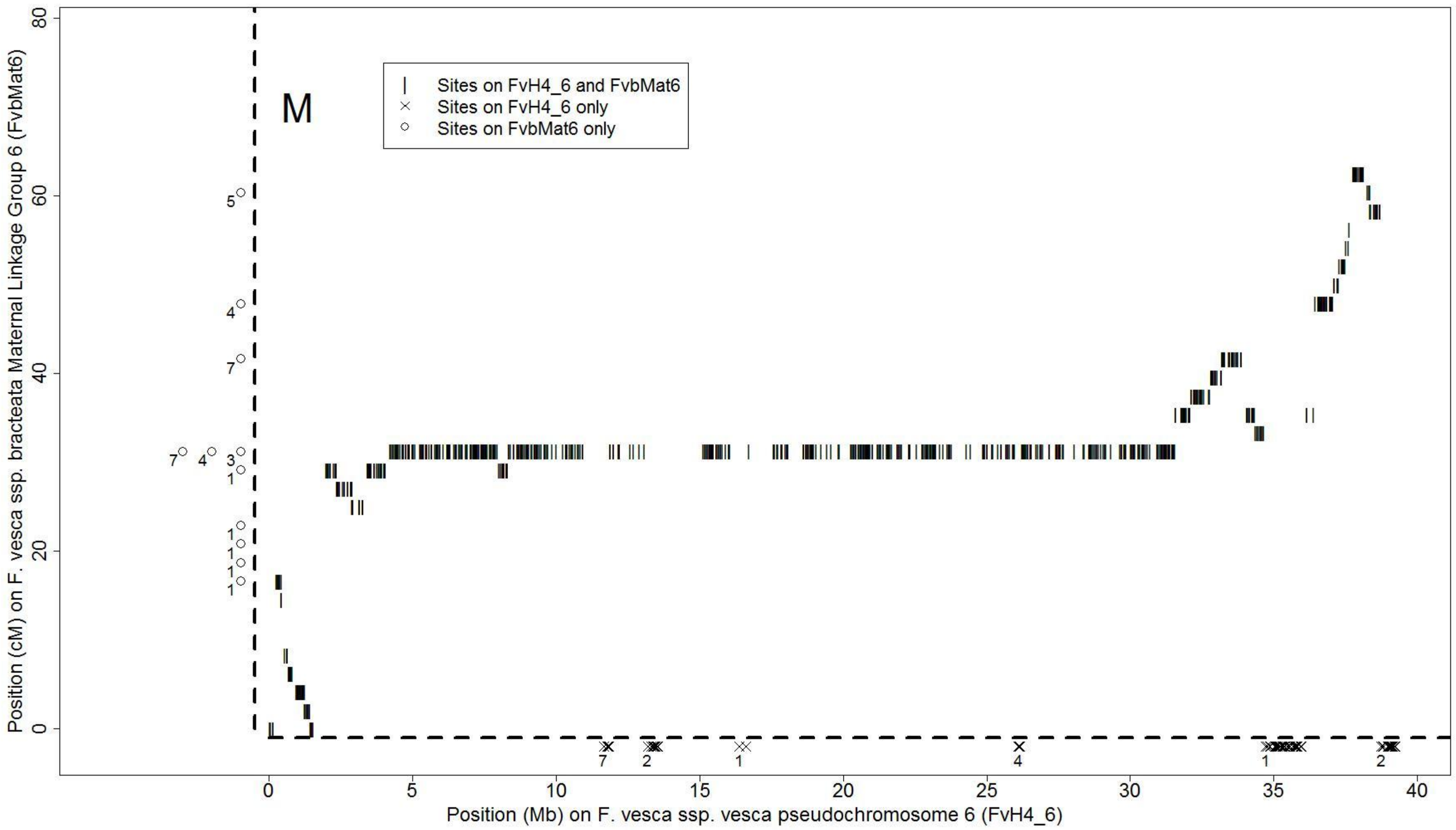
M
Sites on FvH4_6 and FvbMat6
Sites on FvH4_6 only
Sites on FvbMat6 only
Position (cM) on F. vesca ssp. bracteata Maternal Linkage Group 6 (FvbMat6)
Position (Mb) on F. vesca ssp. vesca pseudochromosome 6 (FvH4_6)
0
20
40
60
80
0
5
10
15
20
25
30
35
40

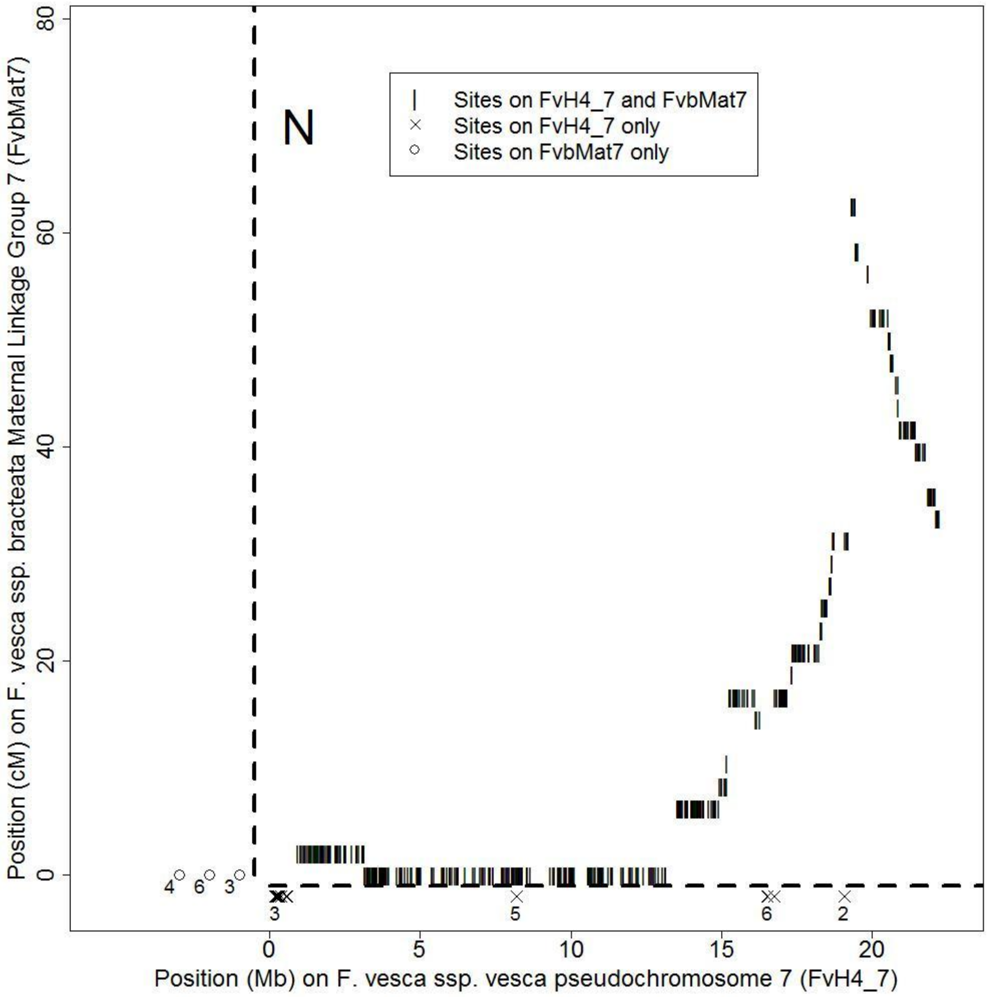

Figure S2 (A-N) Linkage groups in *F. vesca* ssp. *bracteata* plotted against the *F. vesca* ssp. *vesca* reference genome (FvH4). Each short vertical line represents an informative marker in our linkage map. Each linkage group is plotted against the FvH4 pseudochromosome with which it shares the most makers (the main pseudochromosome; Table 1). Markers that aligned somewhere in FvH4 other than the main pseudochromosome are indicated as circles on along left side of the figure, numbered according to FvH4 pseudochromosome. Markers that aligned to the main pseudochromosome but mapped to a different Fvb linkage group are indicated as x marks along the bottom of the figure, numbered according to Fvb linkage group. Markers that map to one part of the linkage group but align to a different part of the main pseudochromosome are within-chromosome translocations.

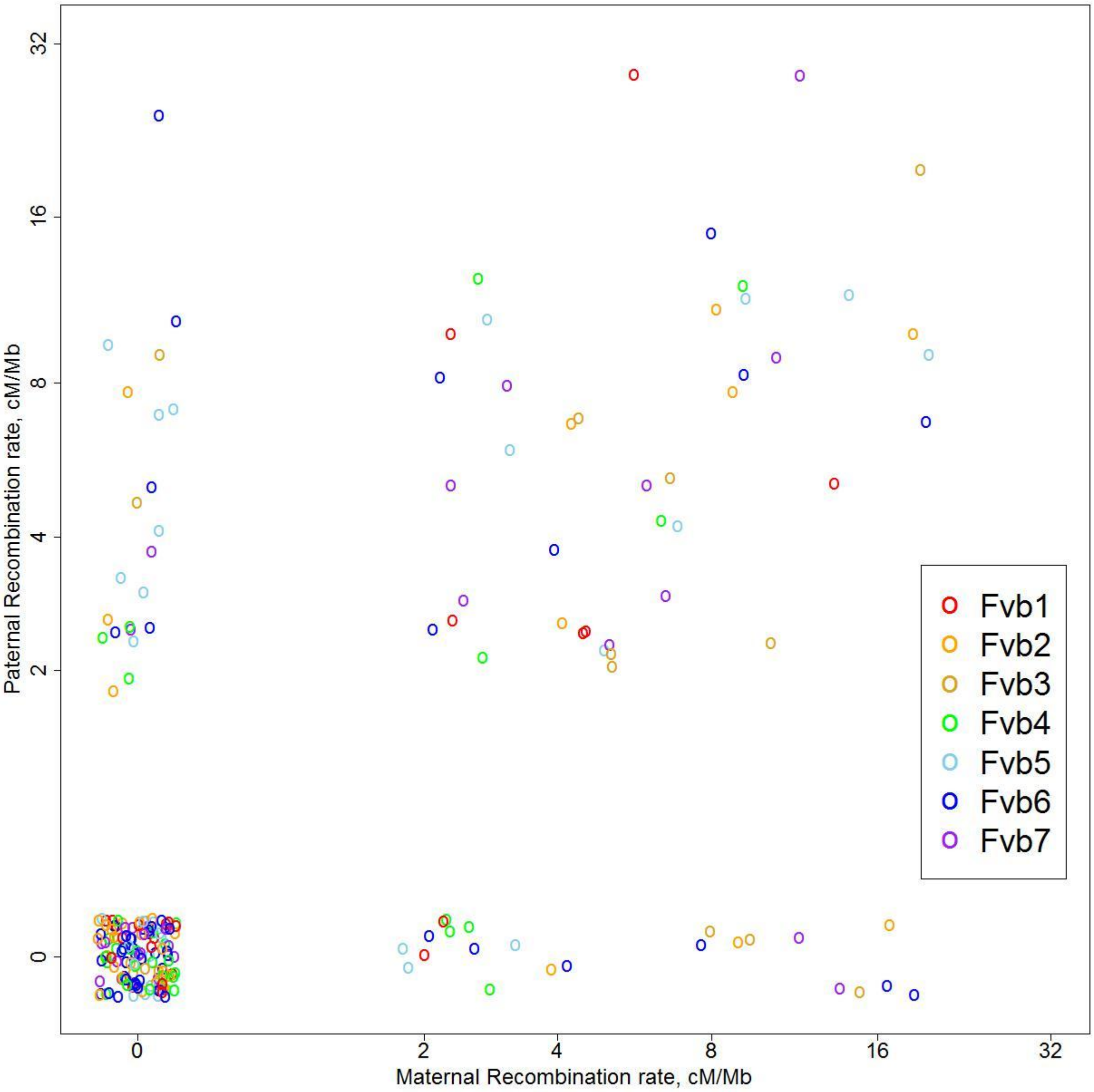

Figure S3 Recombination rates are very similar in both parental maps. Each point represents a ~1Mb genomic region. Points are jittered (random noise added) for ease of visualization, rounded up so that all rates are nonzero, and plotted on a log scale. Recombination rates across the genome in both parents were highly correlated (r_s = 0.48; P < 10^{-13}). Most (60%) genomic regions showed no recombination in either parent, and those showing recombination often showed it in both parents.

File S1

FASTA file of the 6575 targeted regions.

Each region was targeted by three overlapping 100bp probes. Regions are named by chromosome and site position in FvH4.

File S1 is available for download at http://www.g3journal.org/lookup/suppl/doi:10.1534/g3.113.006288/-/DC1.

File S2

Variant call format (vcf) file of all targeted capture genotypes used in linkage mapping.

File S2 is available for download at http://www.g3journal.org/lookup/suppl/doi:10.1534/g3.113.006288/-/DC1.

File S3

Map position (Fvb, in cM) and reference genome position (FvH4, in bp) for all markers.

File S3 is available for download at http://www.g3journal.org/lookup/suppl/doi:10.1534/g3.113.006288/-/DC1.

Table S1 Targeted capture Illumina sequencing data for two parents and 48 offspring.

Plant	Targets[a]	Depth[b]	SNP_Depth[c]	Indel_Depth[d]	Gel_Pool[e]	Hyb_Pool[f]	Flowcell	Index	Reads	Sex
MRD60 (father)	6570	224.31	224.59	216.4	4	B	fc1025	CGATGT	11,902,759	hermaphrodite
MRD30 (mother)	6568	114.52	114.97	101.56	1	D	fc1025	TGACCA	3,053,617	female
MRD30x60.79	6570	228.44	228.78	218.64	none	A	fc1022	CGATGT	23,134,225	hermaphrodite
MRD30x60.80	6571	229.4	229.75	219.18	none	A	fc1022	TGACCA	24,805,423	hermaphrodite
MRD30x60.85	6571	233.47	233.67	227.81	none	A	fc1022	ACAGTG	28,143,687	hermaphrodite
MRD30x60.87	6571	235.05	235.13	232.72	none	A	fc1022	GCCAAT	30,114,562	hermaphrodite
MRD30x60.89	6572	231.43	231.69	223.97	none	A	fc1022	CAGATC	23,520,462	unknown
MRD30x60.90	6571	231.78	232.03	224.55	none	A	fc1022	ATCACG	25,682,095	female
MRD30x60.96	6572	234.94	235.17	228.48	none	A	fc1022	CTTGTA	31,354,180	female
MRD30x60.102	6571	224.6	224.9	215.86	none	A	fc1022	TTAGGC	19,257,936	unknown
MRD30x60.1	6567	60.82	61.1	52.8	5	C	fc1025	ACTTGA	1,830,233	hermaphrodite
MRD30x60.2	6567	93.89	94.35	80.5	10	E	fc1025	GATCAG	3,037,486	unknown
MRD30x60.3	6570	154.37	154.94	137.76	11	D	fc1025	TAGCTT	5,129,835	hermaphrodite
MRD30x60.4	6564	35.31	35.49	30.21	6	B	fc1025	GGCTAC	986,925	female
MRD30x60.6	6570	135.11	135.63	120.21	11	D	fc1025	AGTCAA	4,247,282	female
MRD30x60.7	6571	149.3	149.92	131.68	11	D	fc1025	AGTTCC	4,963,971	female
MRD30x60.14	6568	114.1	114.59	100.16	11	D	fc1025	ATGTCA	3,423,053	unknown
MRD30x60.15	6559	41.04	41.19	36.81	9	C	fc1025	CCGTCC	1,219,685	hermaphrodite
MRD30x60.16	6571	131.83	132.44	114.24	12	E	fc1025	GTAGAG	3,673,595	female
MRD30x60.17	6571	116.42	117	99.93	8	E	fc1025	GTCCGC	3,262,604	female
MRD30x60.28	6570	120.47	120.99	105.65	10	E	fc1025	GTGAAA	3,780,144	hermaphrodite
MRD30x60.29	6568	76.03	76.45	63.89	8	E	fc1025	GTGGCC	2,118,379	hermaphrodite
MRD30x60.34	6571	123.23	123.8	106.76	12	E	fc1025	GTTTCG	3,427,160	hermaphrodite
MRD30x60.37	6570	100.65	101.15	86.35	12	E	fc1025	CGTACG	2,804,322	hermaphrodite
MRD30x60.38	6568	76.23	76.57	66.46	12	E	fc1025	GAGTGG	2,108,575	female
MRD30x60.40	6571	133.51	134.1	116.66	6	B	fc1025	GGTAGC	3,858,155	female
MRD30x60.44	6569	147.61	148.19	131.02	6	B	fc1025	ACTGAT	4,310,075	hermaphrodite
MRD30x60.45	6562	49.09	49.3	42.99	9	C	fc1025	ATGAGC	1,512,152	unknown
MRD30x60.47	6561	36.4	36.57	31.6	4	B	fc1025	ATTCCT	1,002,318	female
MRD30x60.49	6568	72.02	72.28	64.67	4	B	fc1025	CAAAAG	1,999,772	female
MRD30x60.51	6569	68.09	68.36	60.16	4	B	fc1025	CAACTA	1,891,836	hermaphrodite
MRD30x60.52	6568	99.68	100.1	87.57	5	C	fc1025	CACCGG	3,093,930	hermaphrodite
MRD30x60.55	6561	51.81	52.1	43.16	8	E	fc1025	CACGAT	1,372,967	female

MRD30x60.57	6564	48.25	48.49	41.36	3	D	fc1025	CACTCA	1,269,038	unknown
MRD30x60.58	6569	89.26	89.57	80.25	7	C	fc1025	CAGGCG	2,871,991	female
MRD30x60.67	6569	90.48	90.83	80.21	2	B	fc1025	CATGGC	2,469,957	female
MRD30x60.68	6571	185.9	186.43	170.51	10	E	fc1025	CATTTT	6,282,413	hermaphrodite
MRD30x60.70	6567	92.63	92.99	82.13	2	B	fc1025	CCAACA	2,638,079	unknown
MRD30x60.71	6563	35.92	36.11	30.51	3	D	fc1025	CGGAAT	952,421	hermaphrodite
MRD30x60.72	6566	68.03	68.38	57.95	7	C	fc1025	CTAGCT	2,034,633	hermaphrodite
MRD30x60.73	6564	82.72	83.09	72.3	5	C	fc1025	CTATAC	2,413,967	hermaphrodite
MRD30x60.74	6569	104.19	104.58	92.91	7	C	fc1025	CTCAGA	3,264,413	unknown
MRD30x60.78	6568	84.43	84.8	73.94	9	C	fc1025	GCGCTA	2,750,451	female
MRD30x60.82	6570	144.84	145.37	129.35	6	B	fc1025	TAATCG	4,372,706	hermaphrodite
MRD30x60.84	6572	184.49	184.98	170.29	7	C	fc1025	TACAGC	7,423,846	female
MRD30x60.88	6569	87.92	88.27	77.93	5	C	fc1025	TATAAT	2,452,805	female
MRD30x60.92	6567	78.32	78.65	68.91	1	D	fc1025	TCATTC	2,022,507	hermaphrodite
MRD30x60.94	6568	72.72	73.07	62.55	1	D	fc1025	TCCCGA	1,924,070	female
MRD30x60.95	6565	111.83	112.25	99.76	2	B	fc1025	TCGAAG	3,102,468	hermaphrodite
MRD30x60.100	6565	60.37	60.65	52.06	10	E	fc1025	TCGGCA	1,877,322	hermaphrodite

[a]Number of targeted polymorphisms with nonzero depth.

[b]Mean depth (coverage) among all targeted polymorphisms (SNPs and indels).

[c]Mean depth (coverage) among all targeted SNPs.

[d]Mean depth (coverage) among all targeted indels.

[e]Prior to gel extraction, libraries were pooled in groups of 4, as indicated.

[f]A total of 8 or 12 libraries were pooled for a single hybridization reaction, as indicated.

Table S2 Primer sequences and scaffold coordinates for nine informative polymorphic sites. These were identified from the low-coverage genome sequences of the parents on scaffold scB (scf0513158 positions 2,258,778-4,674,928). The phenotyped targeted capture offspring (N=40) and additional offspring from the cross (N=55) with known sexual phenotypes were genotyped at these informative sites by Sanger sequencing (Figure 5).

Locus	Scaffold coordinates[a]	Forward/ Reverse primer sequences (5' - 3')	Annealing temperature °C - Time
scB_1556471	1556327-1556351	For: GTGAATTACATGAAGGTGACTGGGG	54°C - 30 sec
	1556795-1556819	Rev: AAAAGGGGTCATCACATCTGGAAAC	
scB_1644279	1643952-1643977	For: ACTACCAACTAGAGACACACATTCCC	56°C - 30 sec
	1644581-1644605	Rev: GAGAAATGATCCAAGACAGAGAGGC	
scB_1675672	1675540-1675564	For: TATGAAGTGGCGTAGAGAATAGGGC	56°C - 30 sec
	1675835-1675859	Rev: TTAGCAACAACAAAACCCTGGACTC	
scB_1689672	1689392-1689416	For: TACGAGTTGCCTTTTGGAGGTTAGG	56°C - 30 sec
	1689726-1689750	Rev: TCAATTAGTCTTGGCCTTGCTTCTG	
scB_1711631	1711443-1711467	For: CTACAGACCAACACCGTCCAAATAC	56°C - 30 sec
	1711904-1711928	Rev: CTTCTTCTGACCTCTCTGACTTTGC	
scB_1731089	1730768-1730793	For: GCCCTGTTGTAGAAATGAATCTGGAG	56°C - 30 sec
	1731360-1731383	Rev: TTATTCCCCCTACTCACACTCAGC	
scB_1776904	1776703-1776727	For: TTTCATTCCTTCCGACTAAAGCTGC	56°C - 30 sec
	1777051-1777076	Rev: CCACTCACTTTTAACCAGATGCTCAG	
scB_1804106	1803856-1803886	For: ATGGTAGCATAATGCCTCATATAACACTAG	56°C - 30 sec
	1804266-1804293	Rev: GTGAGGTTCTTGCCATCTTTATTCCAC	
scB_2027728	2027451-2027475	For: CAGTTTGTGTATGTGTTTGTTGCGG	56°C - 30 sec
	2028097-2028121	Rev: GCATTTAGATTTCCAAGCGTTAGCC	

[a]Coordinates are with respect to scB (add 2,258,777 to determine the position on scaffold scf0513158 in the *F. vesca* ssp. *vesca* reference genome version 1. 0).

Table S3 Incongruities between FvH4 and Fvb. Listed are scaffolds from FvH4 that map to Fvb linkage groups in ways incompatible with their assembly position in FvH4. Some of these likely represent assembly errors, while others likely represent real translocations or inversions.

Scaffold	Pseudochromosome	Start	End	Linkage_Group	Entire scaffold?[a]	Type
scf0512942	FvH4_0	317810	486710	4	yes	assignment of unanchored scaffold
scf0512960	FvH4_0	771617	922450	5	yes	assignment of unanchored scaffold
scf0512975	FvH4_0	932451	1031722	4	yes	assignment of unanchored scaffold
scf0513018	FvH4_0	1199431	1488070	2	yes	assignment of unanchored scaffold
scf0513028	FvH4_0	1498071	1647224	4	yes	assignment of unanchored scaffold
scf0513041	FvH4_0	2252495	2721037	1	yes	assignment of unanchored scaffold
scf0513045	FvH4_0	2731038	2792572	5	yes	assignment of unanchored scaffold
scf0513068	FvH4_0	2802573	3345236	2	yes	assignment of unanchored scaffold
scf0513081	FvH4_0	4365948	5638289	1	yes	assignment of unanchored scaffold
scf0513119	FvH4_0	5648290	6841401	2	yes	assignment of unanchored scaffold
scf0513137	FvH4_0	7326933	8406320	2	yes	assignment of unanchored scaffold
scf0513148	FvH4_0	9021620	9922368	2	yes	assignment of unanchored scaffold
scf0513155	FvH4_0	9932369	11179145	4	yes	assignment of unanchored scaffold
scf0513189	FvH4_0	11189146	11767950	1,2	no	assignment of unanchored scaffold, interchromosome translocation
scf0513151	FvH4_1	1	1445376	4,1	no	interchromosome translocation, inversion
scf0513105	FvH4_1	1455377	3331343	1	yes	inversion
scf0513114	FvH4_1	3341344	4120631	5	yes	interchromosome translocation
scf0512959	FvH4_1	4829947	5419653	1,5	no	inversion, interchromosome translocation
scf0513095	FvH4_1	13186592	14572914	6	no	interchromosome translocation
scf0513168_1	FvH4_1	17339065	19938125	3,6	no	interchromosome translocation (x2)
scf0512938	FvH4_2	2272727	2456317	2	yes	intrachromosome translocation
scf0513057	FvH4_2	5704770	6000074	4	yes	interchromosome translocation
scf0512968	FvH4_2	17482696	18535496	2	yes	inversion
scf0513134	FvH4_2	18667366	21445916	2	yes	inversion
scf0513194	FvH4_2	21455917	24538926	2	yes	inversion
scf0513171	FvH4_3	1	1497153	3	yes	inversion
scf0513104	FvH4_3	1507154	3362024	3	yes	inversion
scf0513072	FvH4_3	3372025	3727613	6	yes	interchromosome translocation
scf0513017	FvH4_3	3737614	4232872	3	yes	inversion
scf0513082	FvH4_3	4410664	5334373	3	yes	inversion
scf0513173	FvH4_3	6456995	8718722	3	yes	inversion
scf0513089	FvH4_3	9045060	9383133	7	yes	interchromosome translocation

scf0513156	FvH4_3	9809969	11035985	7	yes	interchromosome translocation
scf0513115	FvH4_3	11045986	11903823	5	yes	interchromosome translocation
scf0513053	FvH4_3	15204652	15608968	4	yes	interchromosome translocation
scf0513125	FvH4_3	19509464	21597487	2	yes	interchromosome translocation
scf0513015	FvH4_3	27880400	28601208	3	yes	intrachromosome translocation
scf0513029	FvH4_3	30982798	31368128	3	yes	inversion
scf0513065	FvH4_4	1	206650	6	yes	interchromosome translocation
scf0513090	FvH4_4	7833870	8595096	3	yes	interchromosome translocation
scf0513124_4	FvH4_4	9525261	10260387	6	no	interchromosome translocation
scf0513150	FvH4_4	12599598	14043386	5	no	interchromosome translocation
scf0512935	FvH4_4	14053387	15031722	4	yes	intrachromosome translocation
scf0513004	FvH4_4	22938187	23936647	6	no	interchromosome translocation
scf0513025	FvH4_4	23946648	24163290	4	yes	inversion
scf0513158_4	FvH4_4	24298703	26557413	7	no	interchromosome translocation
scf0513012	FvH4_5	1	128260	3	yes	interchromosome translocation
scf0513098	FvH4_5	138261	3585757	6	no	interchromosome translocation
scf0513052	FvH4_5	3595758	3984026	2	yes	interchromosome translocation
scf0511962	FvH4_5	5833229	5933781	5	yes	intrachromosome translocation
scf0513135	FvH4_5	8572001	11121459	5	yes	inversion
scf0513066	FvH4_5	13220304	13294433	1	yes	interchromosome translocation
scf0513166	FvH4_5	14797581	15196668	5	yes	intrachromosome translocation
scf0513094	FvH4_5	17913724	20360920	5	yes	inversion
scf0513146_5	FvH4_5	20370921	21946032	1	no	interchromosome translocation
scf0513187	FvH4_5	23614770	26212222	4	no	interchromosome translocation
scf0512965	FvH4_5	26222223	26854803	5	yes	intrachromosome translocation
scf0513087	FvH4_5	27369861	27940252	4	no	interchromosome translocation
scf0512963[b]	FvH4_5	27950253	28286745	5	yes	intrachromosome translocation
scf0513011[b]	FvH4_5	28296746	28438568	5	yes	intrachromosome translocation
scf0513168_6	FvH4_6	213555	1544462	5,6	no	interchromosome translocation, inversion
scf0512983	FvH4_6	1554463	1973909	6	yes	intrachromosome translocation
scf0513061	FvH4_6	1983910	3376700	6	yes	inversion
scf0513177	FvH4_6	4210475	8315614	6	yes	inversion
scf0513185	FvH4_6	11066533	11863480	7	yes	interchromosome translocation
scf0513196	FvH4_6	13162357	15497570	2	no	interchromosome translocation
scf0513176	FvH4_6	16117078	18575326	1	no	interchromosome translocation
scf0512952	FvH4_6	19487000	19734822	5	no	interchromosome translocation

scf0513040	FvH4_6	25990763	26193578	4	yes	interchromosome translocation
scf0512991	FvH4_6	34044179	36036831	1, 6	no	interchromosome translocation, intrachromosome translocation
scf0512961	FvH4_6	36046832	36389855	6	yes	intrachromosome translocation
scf0513112	FvH4_6	38173164	39347594	2,6	no	interchromosome translocation, inversion
scf0513170	FvH4_7	1	5155874	1,3,7	no	interchromosome translocation (x2), intrachromosome translocation
scf0513080	FvH4_7	7699732	8246547	5	no	interchromosome translocation
scf0512990[c]	FvH4_7	11408502	12404147	7	no	inversion
scf0512946[c]	FvH4_7	12414148	13183493	7	no	inversion
scf0513113[d]	FvH4_7	15242379	16658559	6,7	no	interchromosome translocation, inversion
scf0513044[d]	FvH4_7	16668560	19276478	2,6	no	interchromosome translocation (x2)
scf0513190	FvH4_7	19286479	22556666	7	yes	inversion

[a]Indicates whether data are compatible with the entire scaffold, and only the entire scaffold, contributing to the incongruity. Such scaffolds may represent assembly errors.

[b]same translocation from FvH4_5 to a different part of Fvb5

[c] same inversion on FvH4_7

[d]same translocation from FvH4_7 to Fvb6
