# Supplementary material for: Targeted Sequence Capture Provides Insight into Genome Structure and Genetics of Male Sterility in a Gynodioecious Diploid Strawberry, Fragaria vesca ssp. bracteata (Rosaceae)
Source: G3 (Bethesda). 2013 Aug 1;3(8):1341–51. doi: 10.1534/g3.113.006288 (PMC3737174; doi:10.1534/g3.113.006288)
Supplement: Supporting Information [file supp_g3.113.006288_FigureS1.pdf]

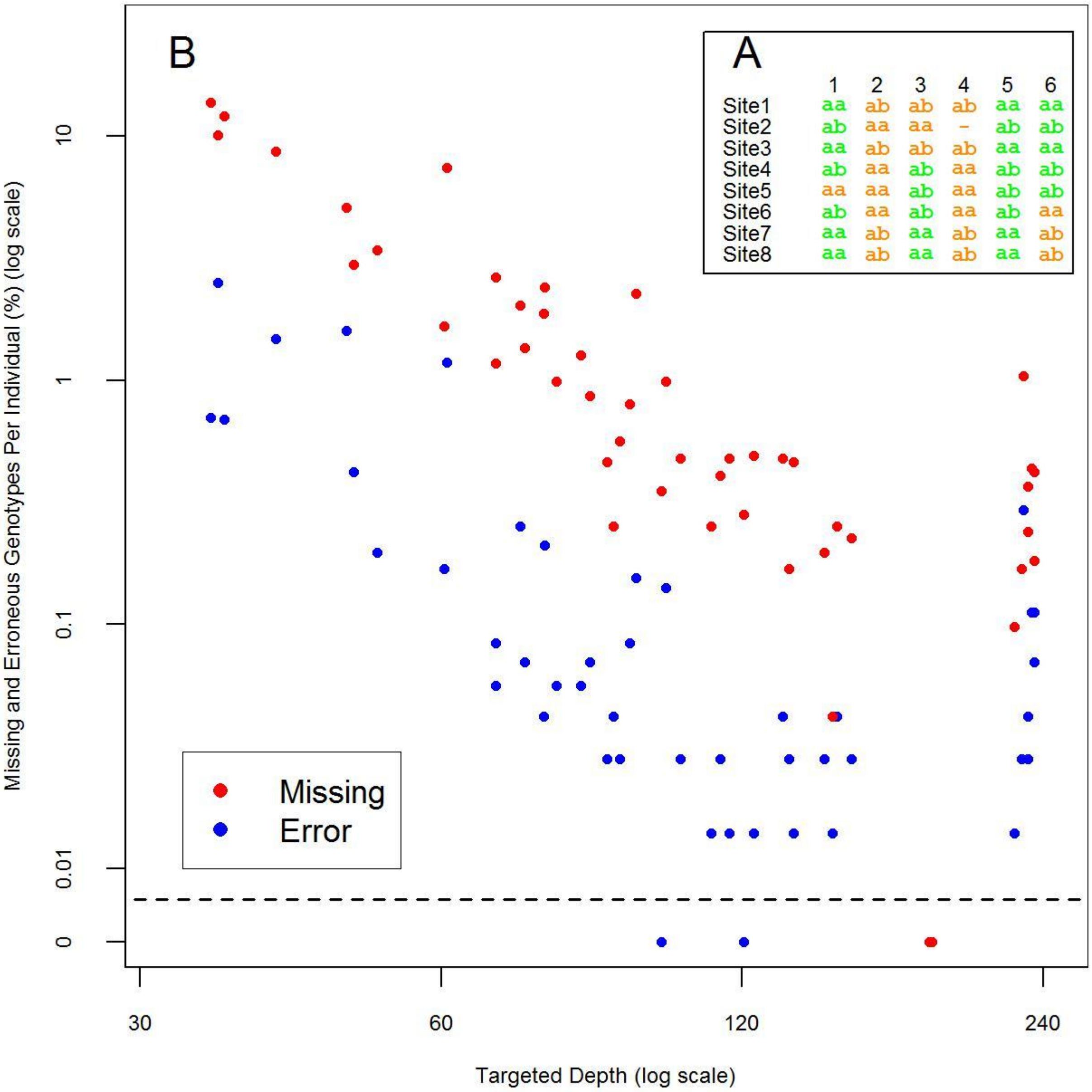

Figure S1 Genotyping errors and missing data. (A) Genotyping errors were identified as anomalies in the linkage map, as shown in this example genotype alignment. Heterozygotes are "ab", homozygotes are "aa", and missing data is "-". Rows are informative sites and columns are individual offspring. Individual 1 has an anomalous genotype at Site 5, in that it does not match what it expected based on the genotypes at Site 4 and Site 6. We would call such a genotype an error rather than a double recombination event. In contrast, the recombination event in Individual 6 that occurs between Site 5 and Site 6 appears to be real because it is supported by multiple sites. Individual 4 has missing data at Site 2 (did not pass quality filter based on Phred-scaled likelihood > 40 for alternate genotypes). (B)Scatterplot of missing data and genotyping errors per individual as a function of coverage (mean depth at targeted sites). We observed low per-individual rates of both missing data (mean = 2.1%; median = 0.6%) and genotyping errors (mean = 0.2%; median = 0.05%). Targeted depth was negatively correlated with both genotyping error (r_s = -0.60; $P < 10^{-05}$) and missing data (r_s = -0.85; $P < 10^{-14}$). For 8 offspring sequenced a separate lane, coverage was much higher (>200x) than for the remaining 40 offspring (Table S1).
