# Supplementary material for: Targeted Sequence Capture Provides Insight into Genome Structure and Genetics of Male Sterility in a Gynodioecious Diploid Strawberry, Fragaria vesca ssp. bracteata (Rosaceae)
Source: G3 (Bethesda). 2013 Aug 1;3(8):1341–51. doi: 10.1534/g3.113.006288 (PMC3737174; doi:10.1534/g3.113.006288)
Supplement: Supporting Information [file supp_g3.113.006288_FigureS2.pdf]

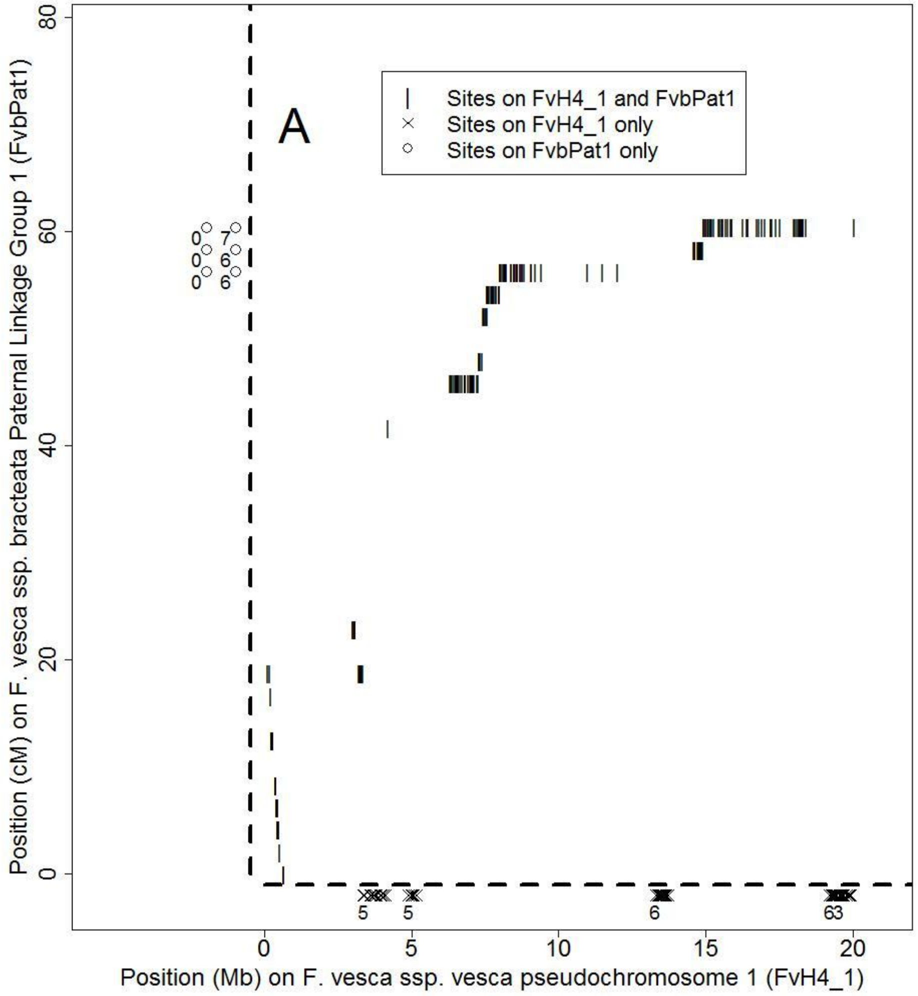

A
Sites on FvH4_1 and FvbPat1
Sites on FvH4_1 only
Sites on FvbPat1 only
Position (cM) on F. vesca ssp. bracteata Paternal Linkage Group 1 (FvbPat1)
Position (Mb) on F. vesca ssp. vesca pseudochromosome 1 (FvH4_1)
0
20
40
60
80
0
5
10
15
20
5
5
6
63
0
0
0
7
6
6

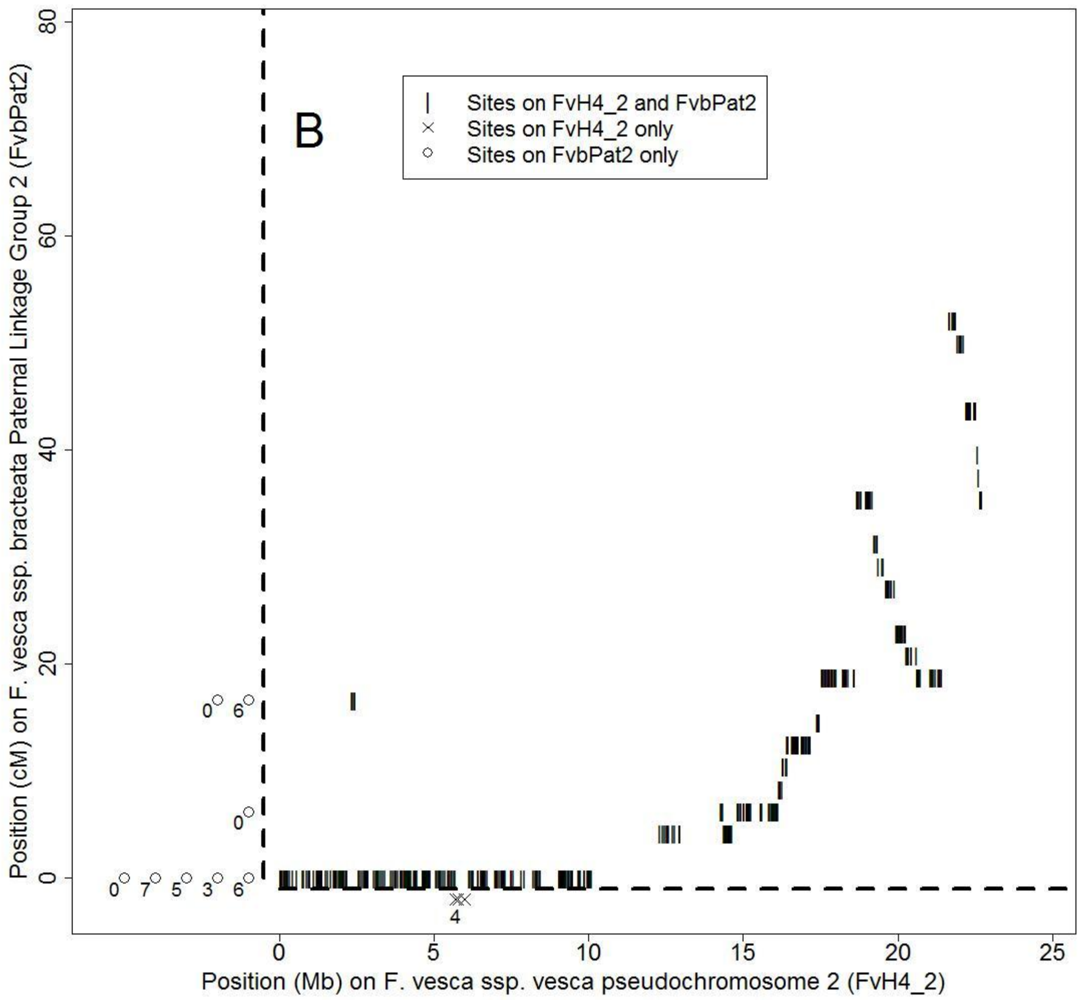
B
Sites on FvH4_2 and FvbPat2
Sites on FvH4_2 only
Sites on FvbPat2 only
Position (cM) on F. vesca ssp. bracteata Paternal Linkage Group 2 (FvbPat2)
Position (Mb) on F. vesca ssp. vesca pseudochromosome 2 (FvH4_2)
0
20
40
60
80
0
5
10
15
20
25

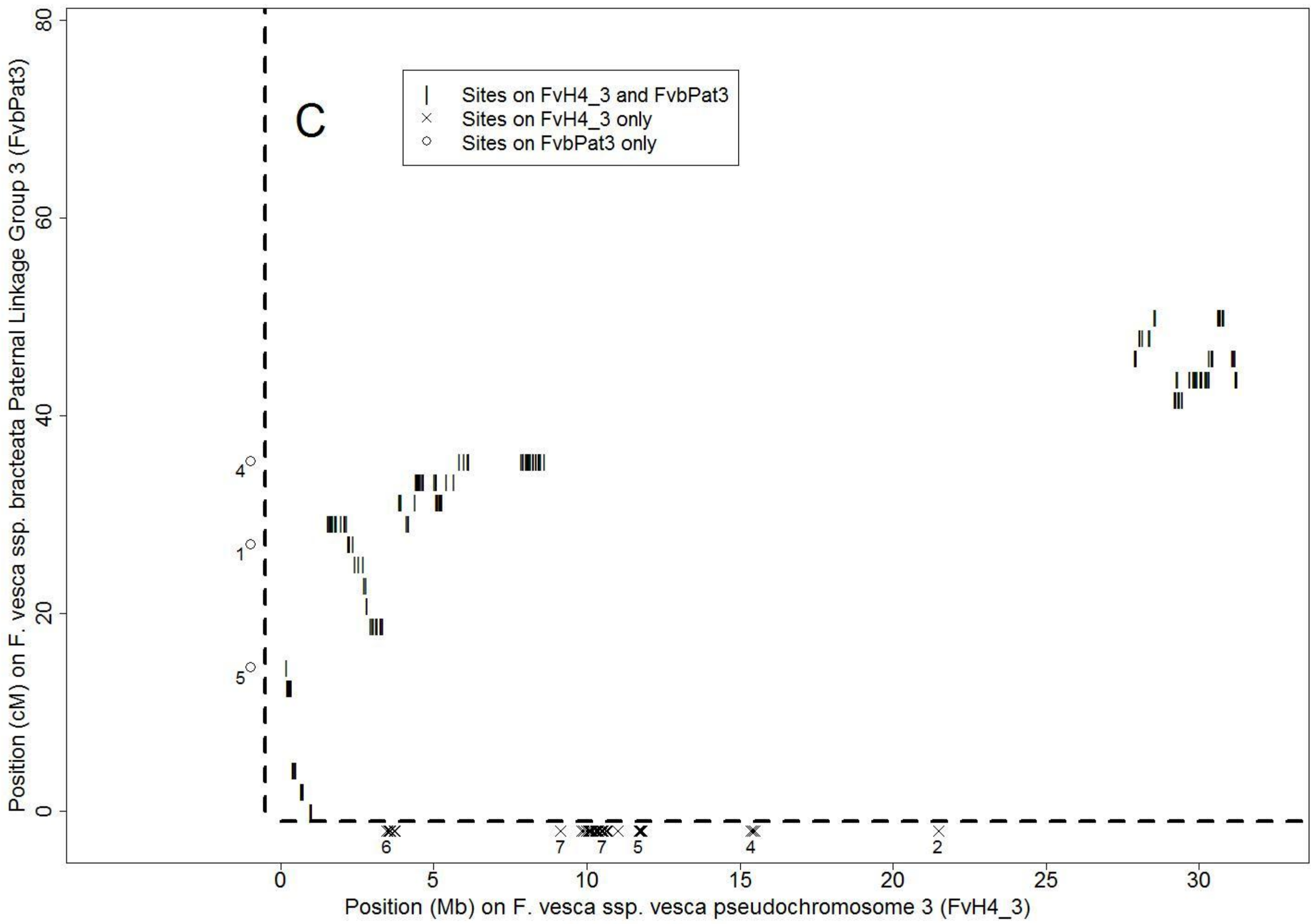

C
Sites on FvH4_3 and FvbPat3
Sites on FvH4_3 only
Sites on FvbPat3 only
Position (cM) on F. vesca ssp. bracteata Paternal Linkage Group 3 (FvbPat3)
Position (Mb) on F. vesca ssp. vesca pseudochromosome 3 (FvH4_3)
0
20
40
60
80
0
5
10
15
20
25
30
4
1
5
6
7
7
5
4
2

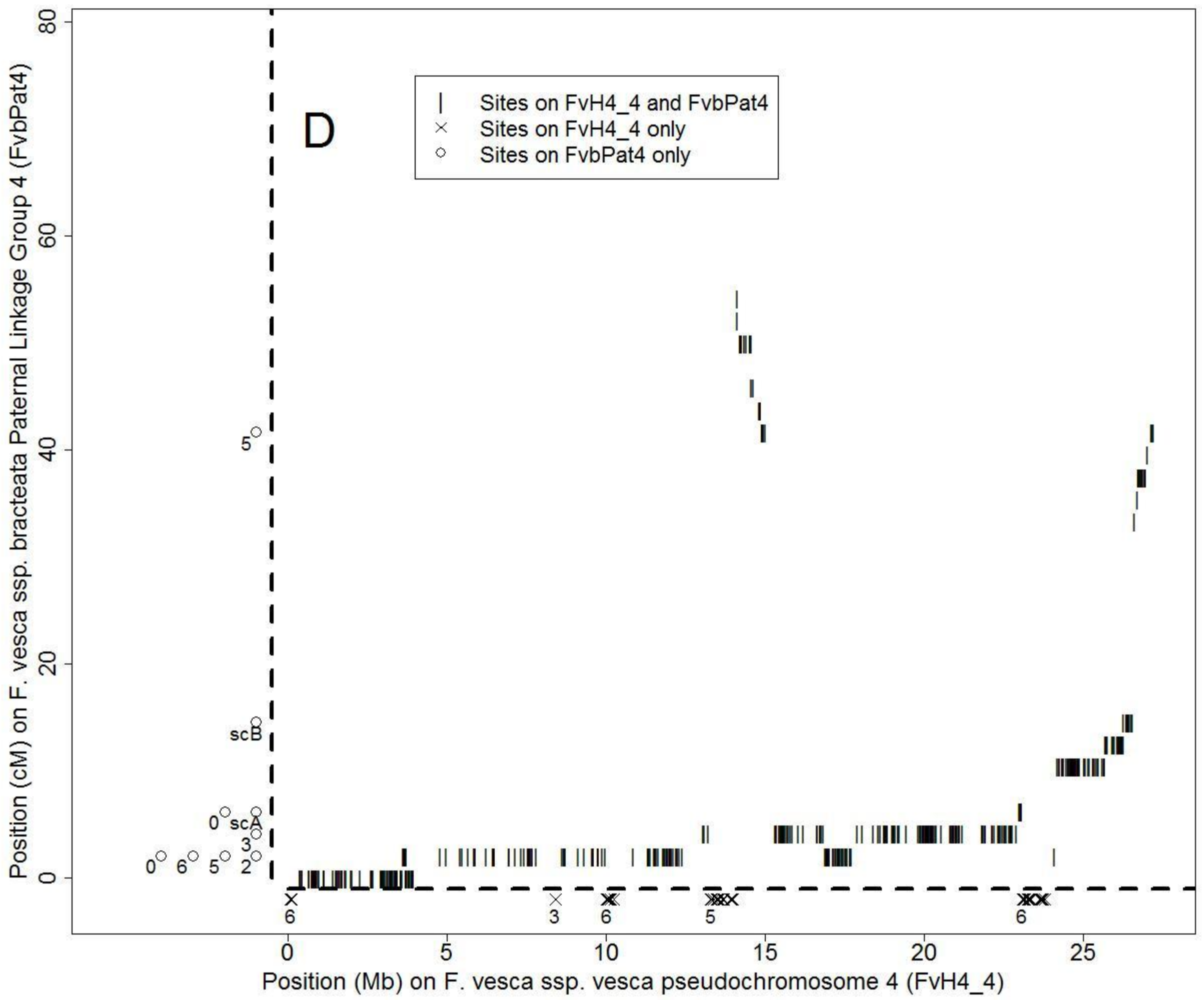
D
Sites on FvH4_4 and FvbPat4
Sites on FvH4_4 only
Sites on FvbPat4 only
Position (cM) on F. vesca ssp. bracteata Paternal Linkage Group 4 (FvbPat4)
Position (Mb) on F. vesca ssp. vesca pseudochromosome 4 (FvH4_4)
0
20
40
60
80
0
5
10
15
20
25
scB
scA

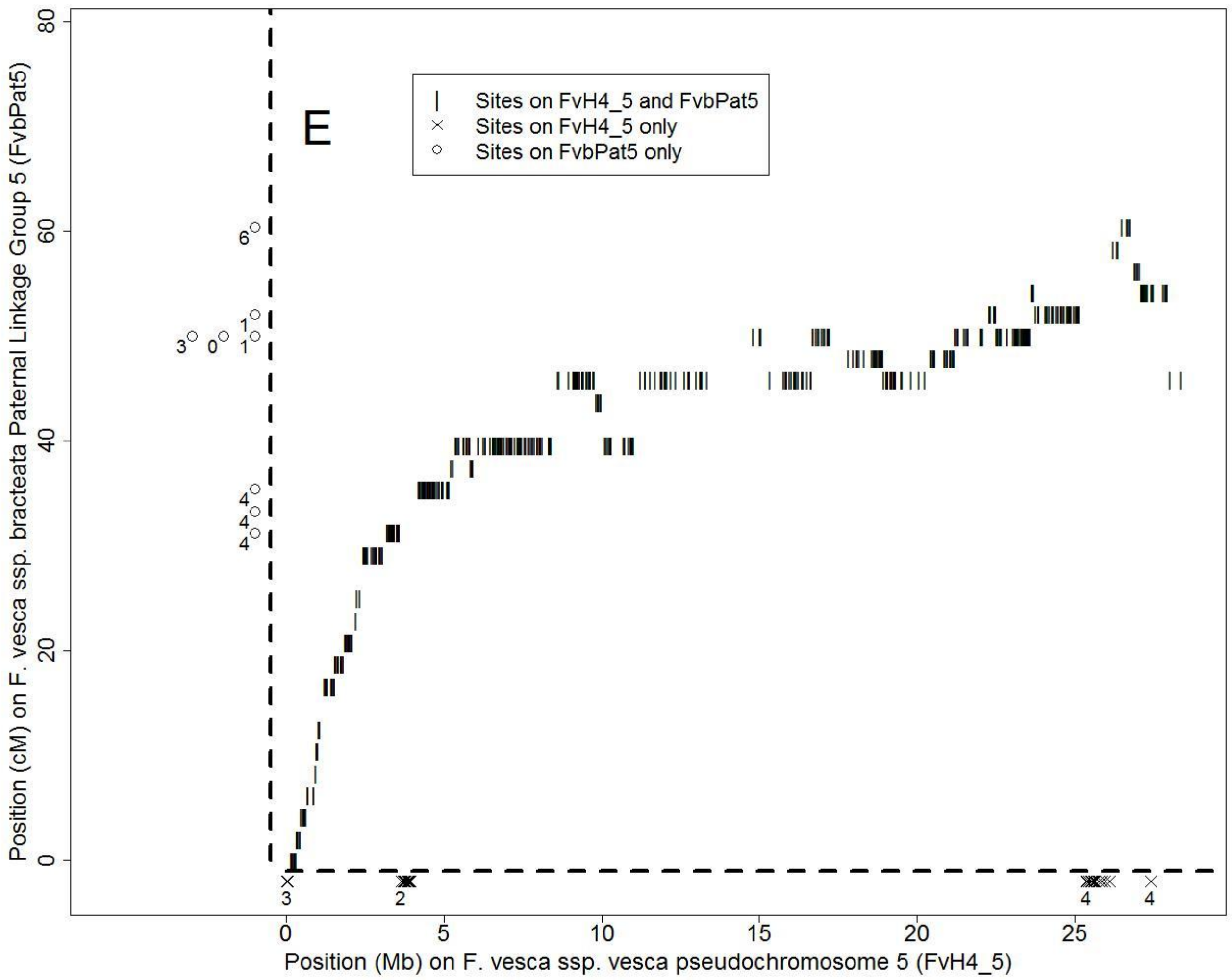
E
Sites on FvH4_5 and FvbPat5
Sites on FvH4_5 only
Sites on FvbPat5 only
Position (cM) on F. vesca ssp. bracteata Paternal Linkage Group 5 (FvbPat5)
Position (Mb) on F. vesca ssp. vesca pseudochromosome 5 (FvH4_5)

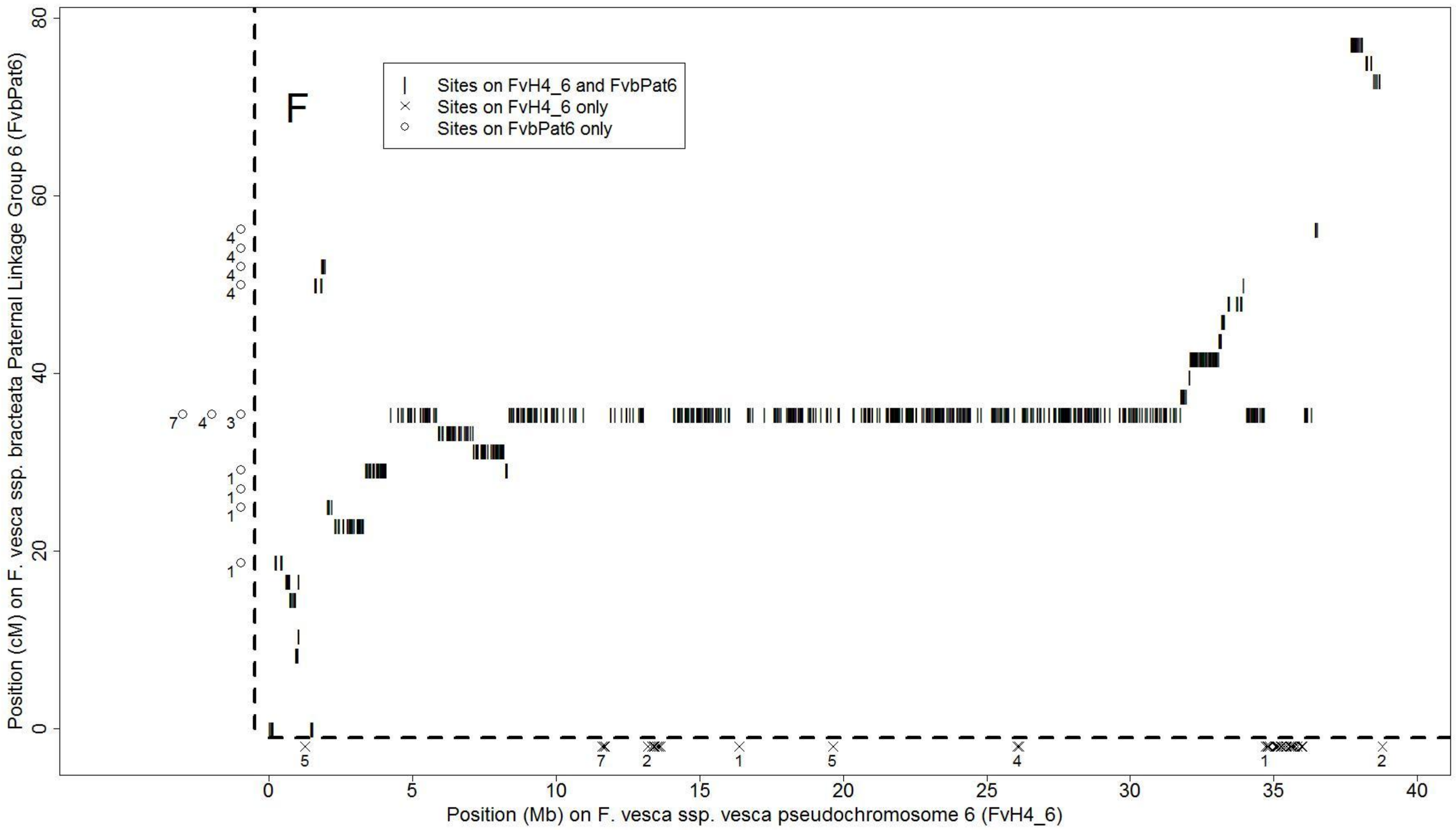
F
Sites on FvH4_6 and FvbPat6
Sites on FvH4_6 only
Sites on FvbPat6 only
Position (cM) on F. vesca ssp. bracteata Paternal Linkage Group 6 (FvbPat6)
Position (Mb) on F. vesca ssp. vesca pseudochromosome 6 (FvH4_6)
0
20
40
60
80
0
5
10
15
20
25
30
35
40

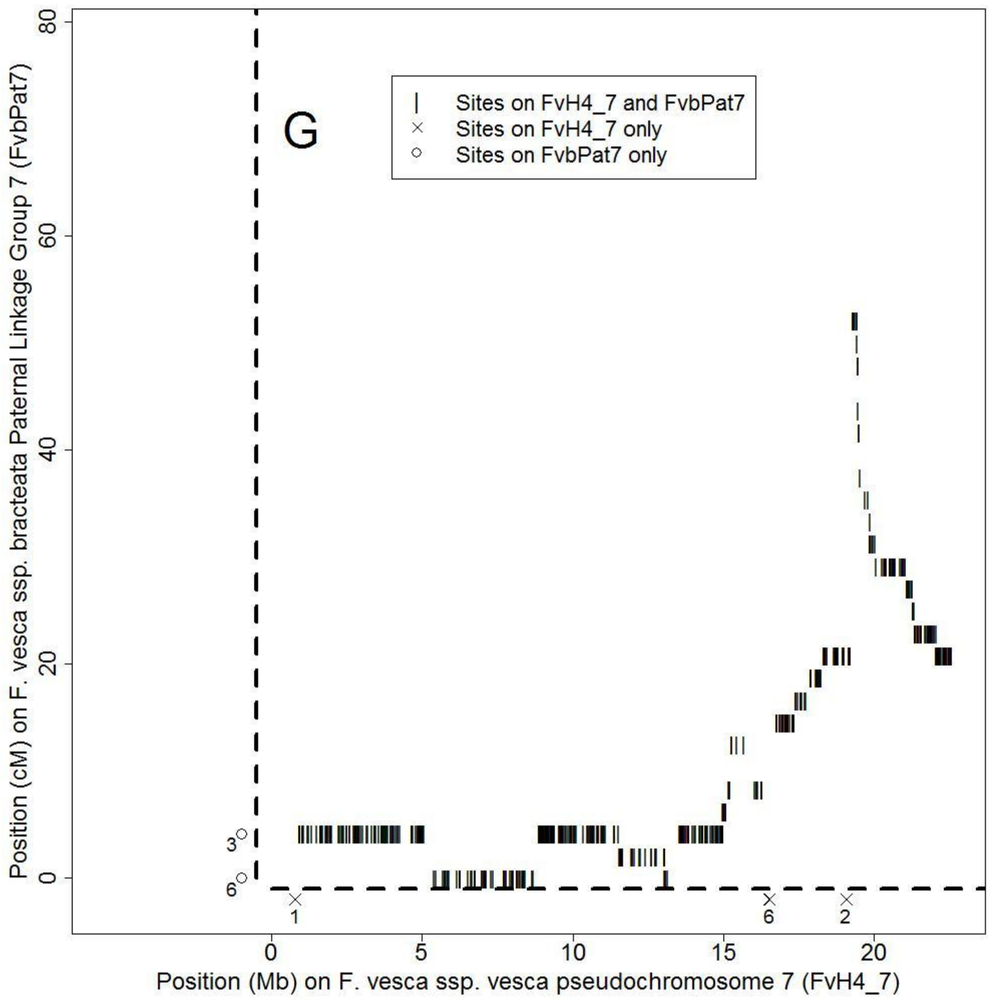
G
Sites on FvH4_7 and FvbPat7
Sites on FvH4_7 only
Sites on FvbPat7 only
Position (cM) on F. vesca ssp. bracteata Paternal Linkage Group 7 (FvbPat7)
Position (Mb) on F. vesca ssp. vesca pseudochromosome 7 (FvH4_7)
0
20
40
60
80
0
5
10
15
20
3
6
1
6
2

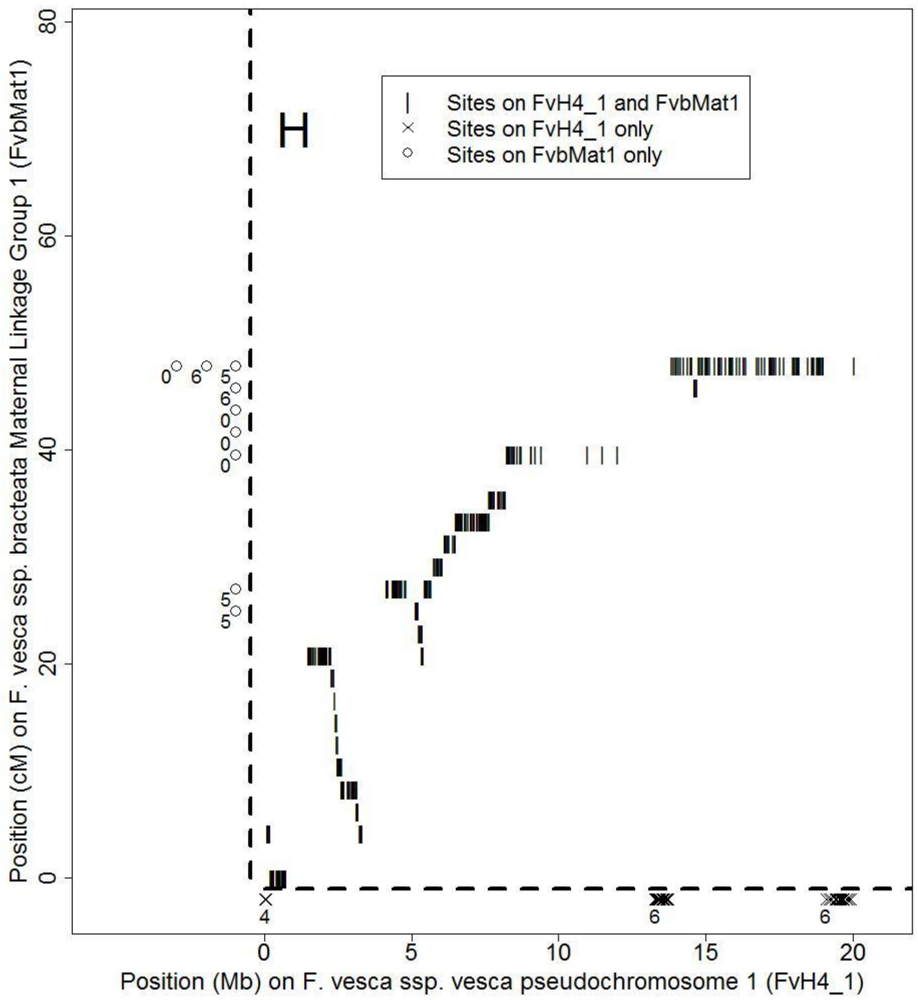
H
Sites on FvH4_1 and FvbMat1
Sites on FvH4_1 only
Sites on FvbMat1 only
Position (cM) on F. vesca ssp. bracteata Maternal Linkage Group 1 (FvbMat1)
Position (Mb) on F. vesca ssp. vesca pseudochromosome 1 (FvH4_1)
0
20
40
60
80
0
5
10
15
20

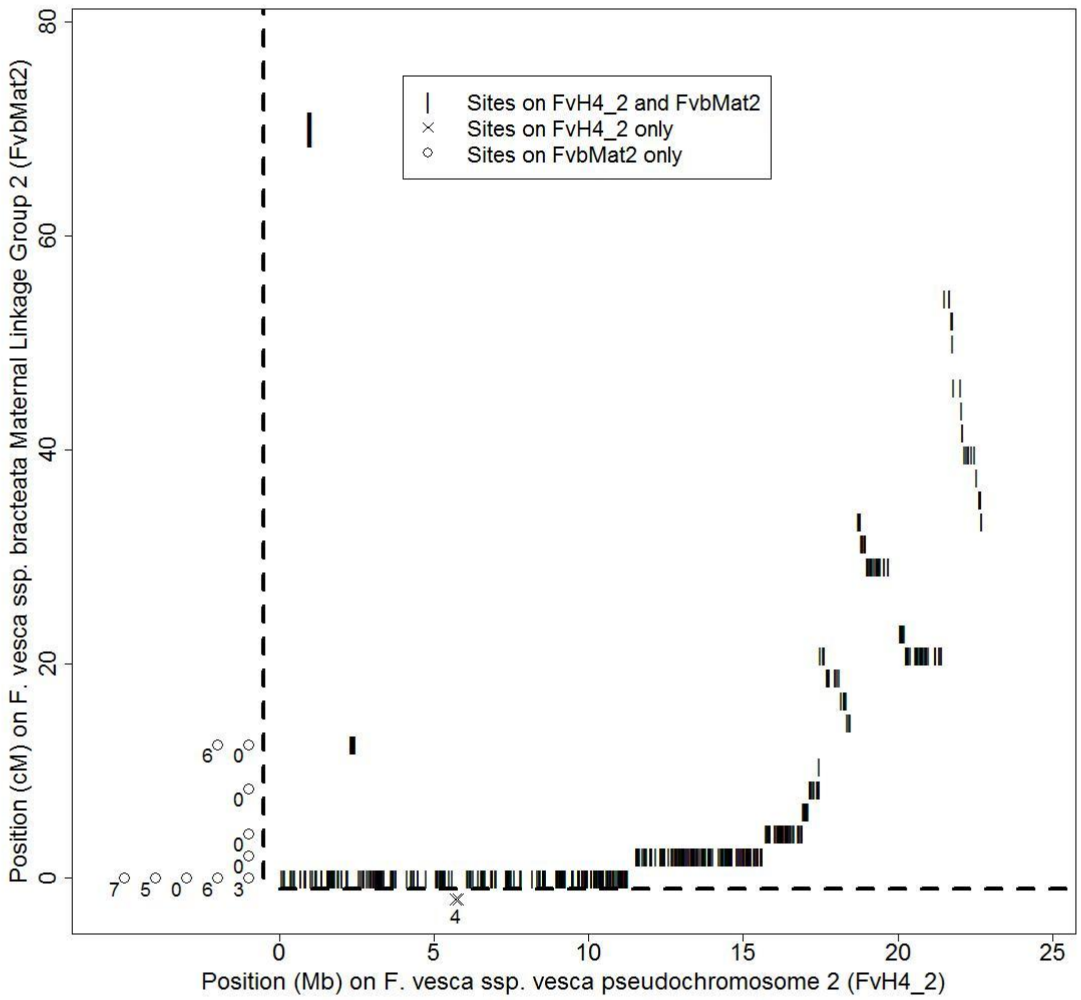
Sites on FvH4_2 and FvbMat2
Sites on FvH4_2 only
Sites on FvbMat2 only
Position (cM) on F. vesca ssp. bracteata Maternal Linkage Group 2 (FvbMat2)
Position (Mb) on F. vesca ssp. vesca pseudochromosome 2 (FvH4_2)
0
20
40
60
80
0
5
10
15
20
25
7
5
0
6
3
6
0
0
0
0
4

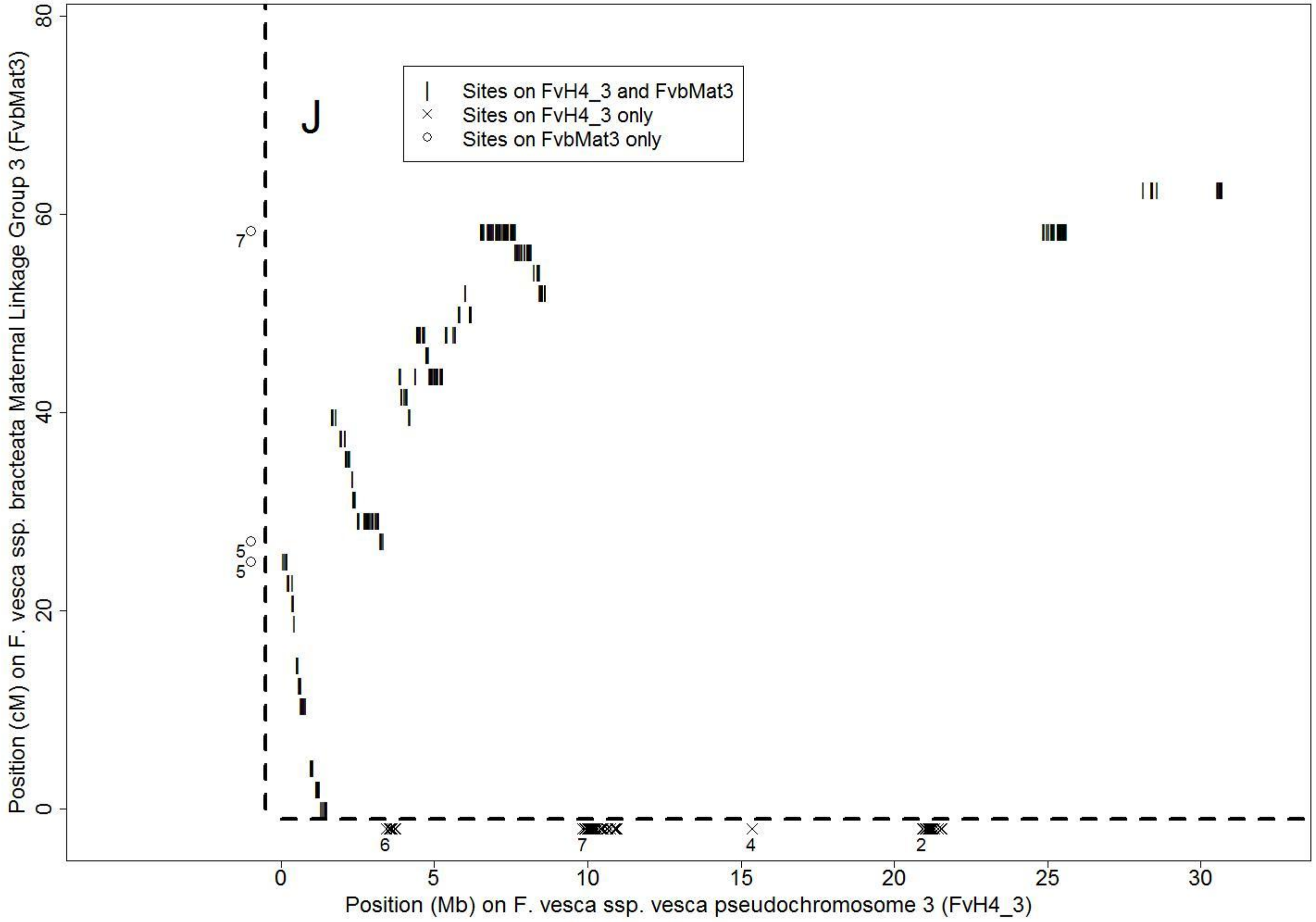
J
Sites on FvH4_3 and FvbMat3
Sites on FvH4_3 only
Sites on FvbMat3 only
Position (cM) on F. vesca ssp. bracteata Maternal Linkage Group 3 (FvbMat3)
Position (Mb) on F. vesca ssp. vesca pseudochromosome 3 (FvH4_3)
0
5
10
15
20
25
30
0
20
40
60
80
7
5
5
6
7
4
2

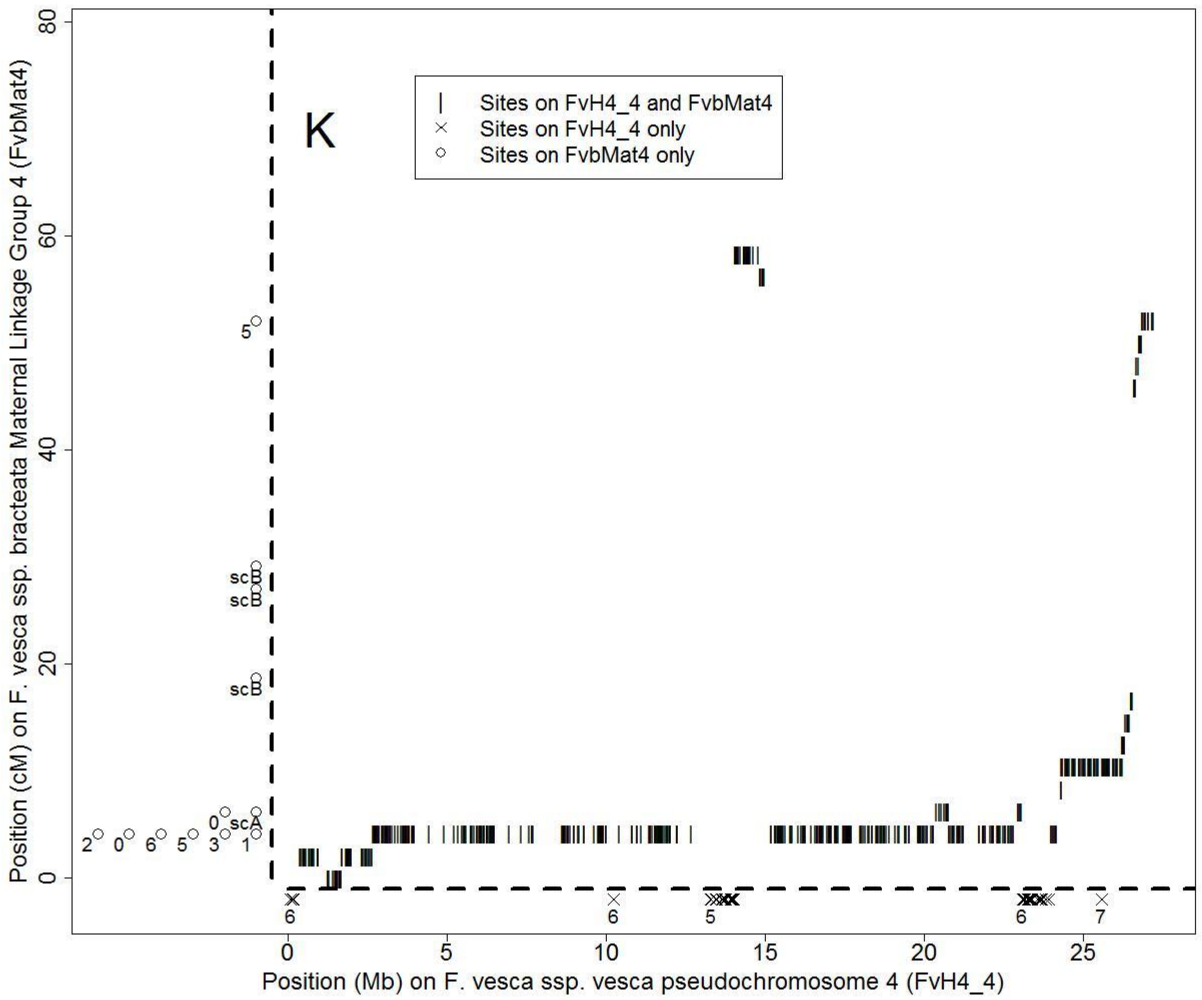
K
Sites on FvH4_4 and FvbMat4
Sites on FvH4_4 only
Sites on FvbMat4 only
Position (cM) on F. vesca ssp. bracteata Maternal Linkage Group 4 (FvbMat4)
Position (Mb) on F. vesca ssp. vesca pseudochromosome 4 (FvH4_4)
0
20
40
60
80
0
5
10
15
20
25
scA
scB
scB
scB

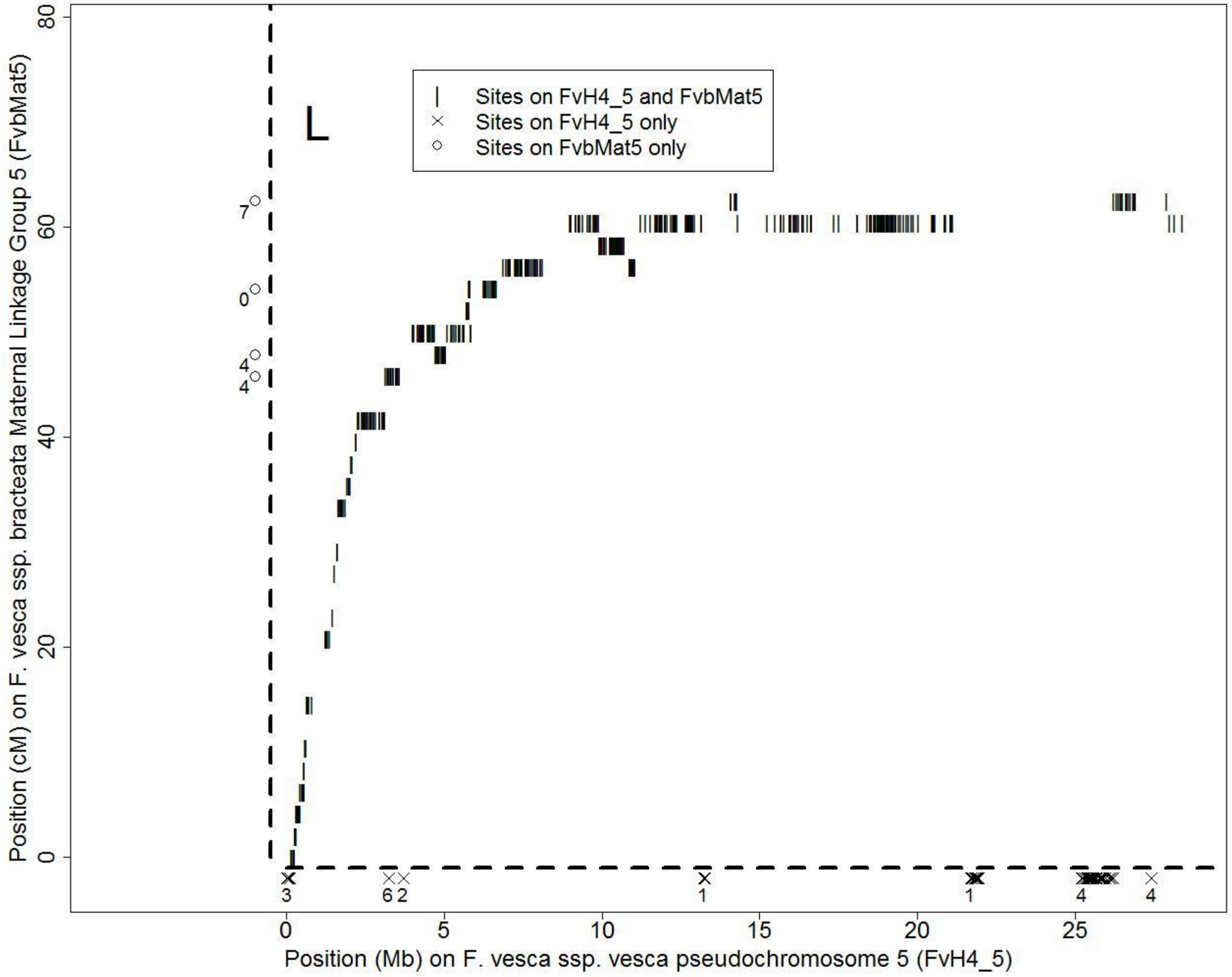

L
Sites on FvH4_5 and FvbMat5
Sites on FvH4_5 only
Sites on FvbMat5 only
Position (cM) on F. vesca ssp. bracteata Maternal Linkage Group 5 (FvbMat5)
Position (Mb) on F. vesca ssp. vesca pseudochromosome 5 (FvH4_5)
0
20
40
60
80
0
5
10
15
20
25
7
0
4
4
3
6 2
1
1
4
4

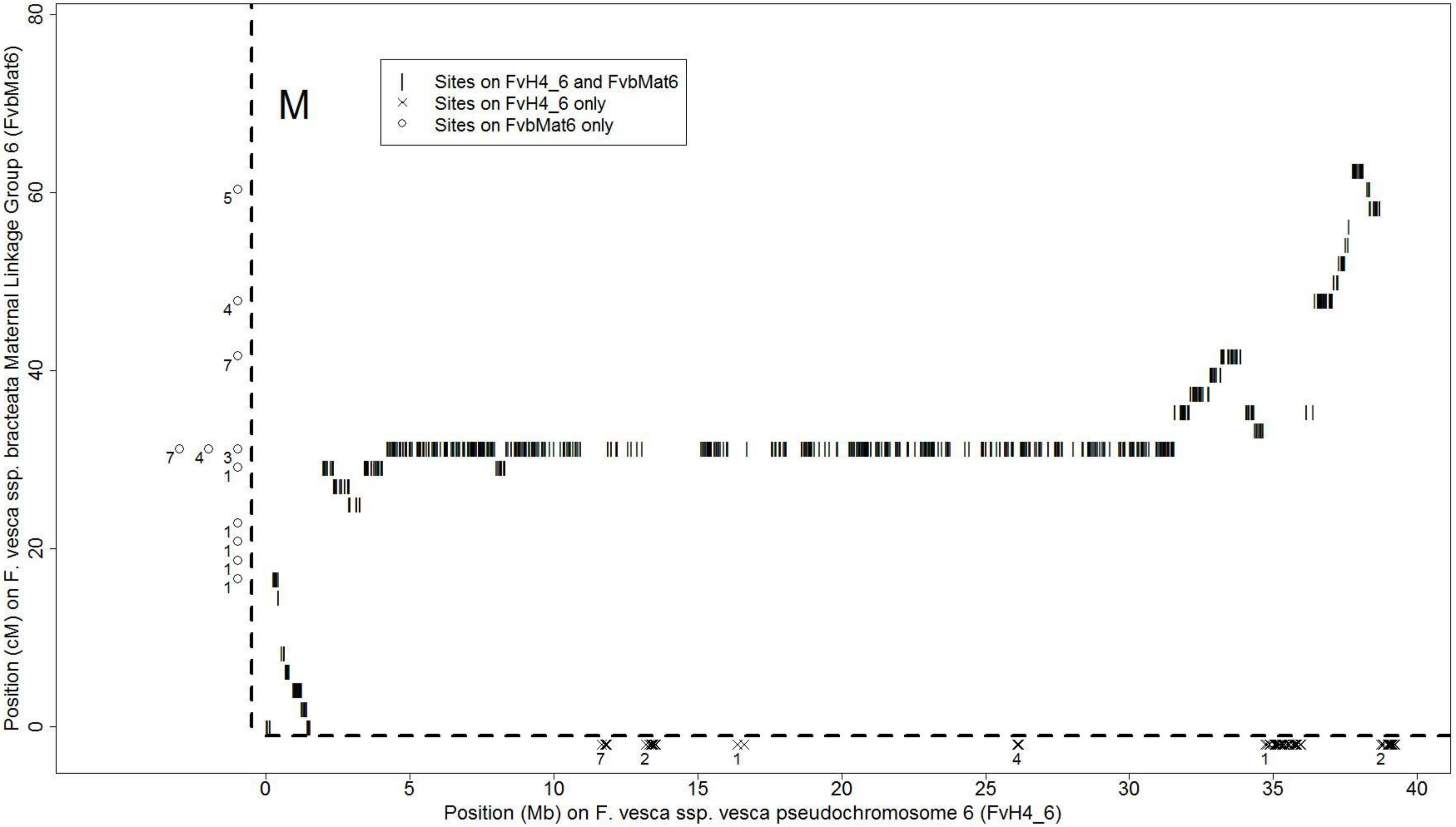
Sites on FvH4_6 and FvbMat6
Sites on FvH4_6 only
Sites on FvbMat6 only
M
Position (cM) on F. vesca ssp. bracteata Maternal Linkage Group 6 (FvbMat6)
Position (Mb) on F. vesca ssp. vesca pseudochromosome 6 (FvH4_6)
0
5
10
15
20
25
30
35
40
0
20
40
60
80

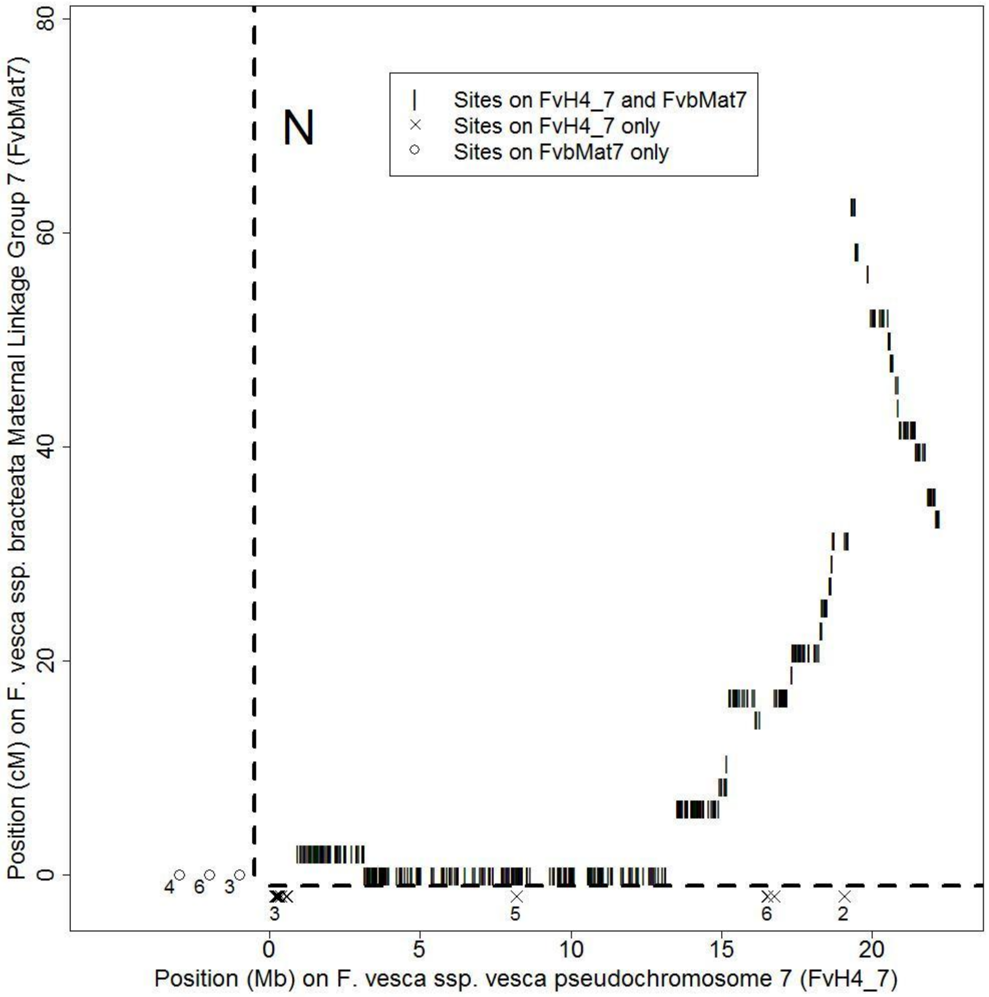


**Figure S2 (A-N)** Linkage groups in *F. vesca* ssp. *bracteata* plotted against the *F. vesca* ssp. *vesca* reference genome (FvH4). Each short vertical line represents an informative marker in our linkage map. Each linkage group is plotted against the FvH4 pseudochromosome with which it shares the most makers (the main pseudochromosome; Table 1). Markers that aligned somewhere in FvH4 other than the main pseudochromosome are indicated as circles on along left side of the figure, numbered according to FvH4 pseudochromosome. Markers that aligned to the main pseudochromosome but mapped to a different Fvb linkage group are indicated as x marks along the bottom of the figure, numbered according to Fvb linkage group. Markers that map to one part of the linkage group but align to a different part of the main pseudochromosome are within-chromosome translocations.
