# Supplementary material for: Targeted Sequence Capture Provides Insight into Genome Structure and Genetics of Male Sterility in a Gynodioecious Diploid Strawberry, Fragaria vesca ssp. bracteata (Rosaceae)
Source: G3 (Bethesda). 2013 Aug 1;3(8):1341–51. doi: 10.1534/g3.113.006288 (PMC3737174; doi:10.1534/g3.113.006288)
Supplement: Supporting Information [file supp_g3.113.006288_FigureS3.pdf]

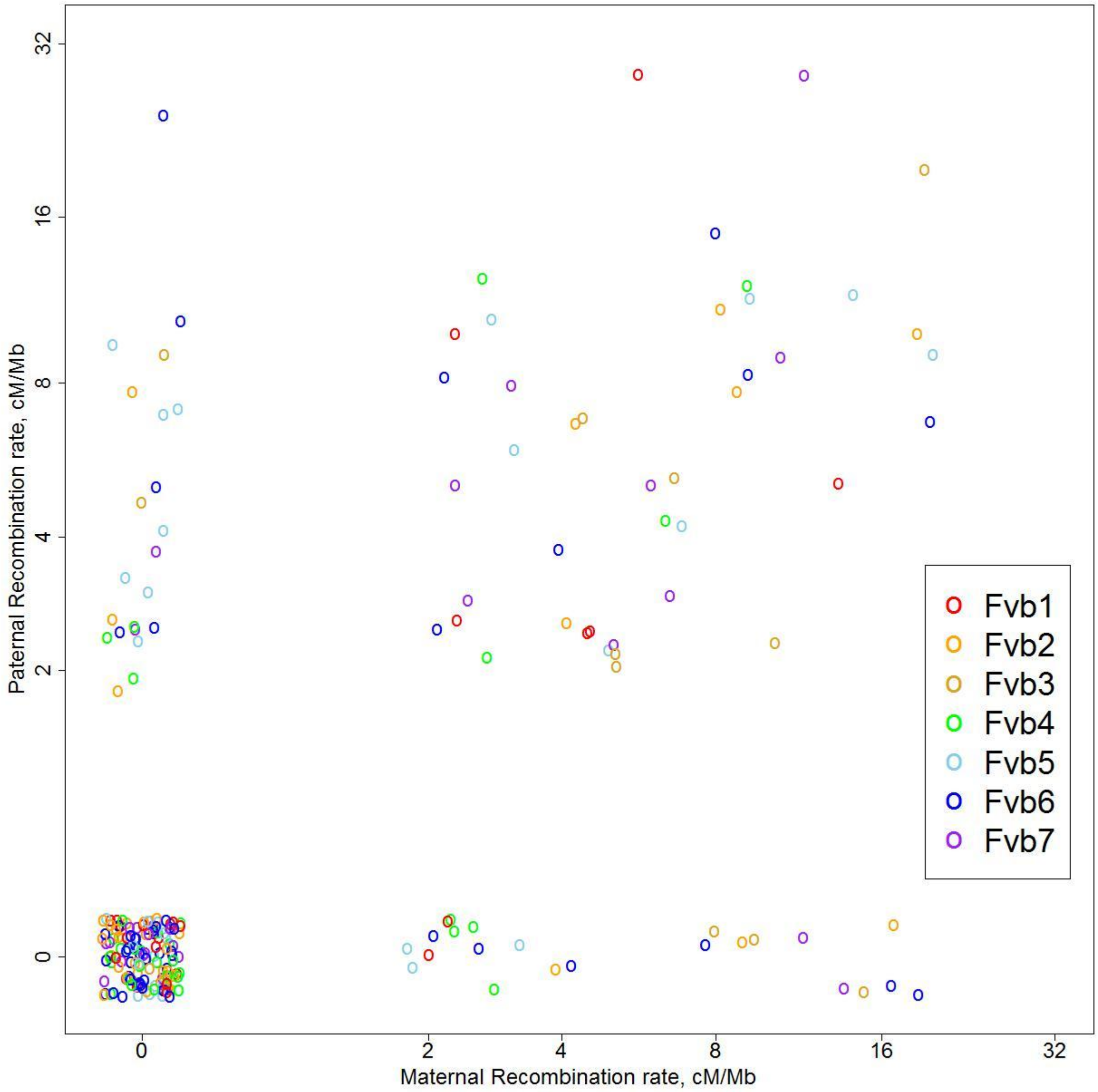

Figure S3 Recombination rates are very similar in both parental maps. Each point represents a ~1Mb genomic region. Points are jittered (random noise added) for ease of visualization, rounded up so that all rates are nonzero, and plotted on a log scale. Recombination rates across the genome in both parents were highly correlated ($r_s = 0.48$; $P < 10^{-13}$). Most (60%) genomic regions showed no recombination in either parent, and those showing recombination often showed it in both parents.
