# Supplementary material for: Targeted Sequence Capture Provides Insight into Genome Structure and Genetics of Male Sterility in a Gynodioecious Diploid Strawberry, Fragaria vesca ssp. bracteata (Rosaceae)
Source: G3 (Bethesda). 2013 Aug 1;3(8):1341–51. doi: 10.1534/g3.113.006288 (PMC3737174; doi:10.1534/g3.113.006288)
Supplement: Supporting Information [file supp_g3.113.006288_TableS1.pdf]

Table S1 Targeted capture Illumina sequencing data for two parents and 48 offspring.

Plant	Targets[a]	Depth[b]	SNP_Depth[c]	Indel_Depth[d]	Gel_Pool[e]	Hyb_Pool[f]	Flowcell	Index	Reads	Sex
MRD60 (father)	6570	224.31	224.59	216.4	4	B	fc1025	CGATGT	11,902,759	hermaphrodite
MRD30 (mother)	6568	114.52	114.97	101.56	1	D	fc1025	TGACCA	3,053,617	female
MRD30x60.79	6570	228.44	228.78	218.64	none	A	fc1022	CGATGT	23,134,225	hermaphrodite
MRD30x60.80	6571	229.4	229.75	219.18	none	A	fc1022	TGACCA	24,805,423	hermaphrodite
MRD30x60.85	6571	233.47	233.67	227.81	none	A	fc1022	ACAGTG	28,143,687	hermaphrodite
MRD30x60.87	6571	235.05	235.13	232.72	none	A	fc1022	GCCAAT	30,114,562	hermaphrodite
MRD30x60.89	6572	231.43	231.69	223.97	none	A	fc1022	CAGATC	23,520,462	unknown
MRD30x60.90	6571	231.78	232.03	224.55	none	A	fc1022	ATCACG	25,682,095	female
MRD30x60.96	6572	234.94	235.17	228.48	none	A	fc1022	CTTGTA	31,354,180	female
MRD30x60.102	6571	224.6	224.9	215.86	none	A	fc1022	TTAGGC	19,257,936	unknown
MRD30x60.1	6567	60.82	61.1	52.8	5	C	fc1025	ACTTGA	1,830,233	hermaphrodite
MRD30x60.2	6567	93.89	94.35	80.5	10	E	fc1025	GATCAG	3,037,486	unknown
MRD30x60.3	6570	154.37	154.94	137.76	11	D	fc1025	TAGCTT	5,129,835	hermaphrodite
MRD30x60.4	6564	35.31	35.49	30.21	6	B	fc1025	GGCTAC	986,925	female
MRD30x60.6	6570	135.11	135.63	120.21	11	D	fc1025	AGTCAA	4,247,282	female
MRD30x60.7	6571	149.3	149.92	131.68	11	D	fc1025	AGTTCC	4,963,971	female
MRD30x60.14	6568	114.1	114.59	100.16	11	D	fc1025	ATGTCA	3,423,053	unknown
MRD30x60.15	6559	41.04	41.19	36.81	9	C	fc1025	CCGTCC	1,219,685	hermaphrodite
MRD30x60.16	6571	131.83	132.44	114.24	12	E	fc1025	GTAGAG	3,673,595	female
MRD30x60.17	6571	116.42	117	99.93	8	E	fc1025	GTCCGC	3,262,604	female
MRD30x60.28	6570	120.47	120.99	105.65	10	E	fc1025	GTGAAA	3,780,144	hermaphrodite
MRD30x60.29	6568	76.03	76.45	63.89	8	E	fc1025	GTGGCC	2,118,379	hermaphrodite
MRD30x60.34	6571	123.23	123.8	106.76	12	E	fc1025	GTTTCG	3,427,160	hermaphrodite
MRD30x60.37	6570	100.65	101.15	86.35	12	E	fc1025	CGTACG	2,804,322	hermaphrodite
MRD30x60.38	6568	76.23	76.57	66.46	12	E	fc1025	GAGTGG	2,108,575	female
MRD30x60.40	6571	133.51	134.1	116.66	6	B	fc1025	GGTAGC	3,858,155	female
MRD30x60.44	6569	147.61	148.19	131.02	6	B	fc1025	ACTGAT	4,310,075	hermaphrodite
MRD30x60.45	6562	49.09	49.3	42.99	9	C	fc1025	ATGAGC	1,512,152	unknown
MRD30x60.47	6561	36.4	36.57	31.6	4	B	fc1025	ATTCCT	1,002,318	female
MRD30x60.49	6568	72.02	72.28	64.67	4	B	fc1025	CAAAAG	1,999,772	female
MRD30x60.51	6569	68.09	68.36	60.16	4	B	fc1025	CAACTA	1,891,836	hermaphrodite
MRD30x60.52	6568	99.68	100.1	87.57	5	C	fc1025	CACCGG	3,093,930	hermaphrodite
MRD30x60.55	6561	51.81	52.1	43.16	8	E	fc1025	CACGAT	1,372,967	female

MRD30x60.57	6564	48.25	48.49	41.36	3	D	fc1025	CACTCA	1,269,038	unknown
MRD30x60.58	6569	89.26	89.57	80.25	7	C	fc1025	CAGGCG	2,871,991	female
MRD30x60.67	6569	90.48	90.83	80.21	2	B	fc1025	CATGGC	2,469,957	female
MRD30x60.68	6571	185.9	186.43	170.51	10	E	fc1025	CATTTT	6,282,413	hermaphrodite
MRD30x60.70	6567	92.63	92.99	82.13	2	B	fc1025	CCAACA	2,638,079	unknown
MRD30x60.71	6563	35.92	36.11	30.51	3	D	fc1025	CGGAAT	952,421	hermaphrodite
MRD30x60.72	6566	68.03	68.38	57.95	7	C	fc1025	CTAGCT	2,034,633	hermaphrodite
MRD30x60.73	6564	82.72	83.09	72.3	5	C	fc1025	CTATAC	2,413,967	hermaphrodite
MRD30x60.74	6569	104.19	104.58	92.91	7	C	fc1025	CTCAGA	3,264,413	unknown
MRD30x60.78	6568	84.43	84.8	73.94	9	C	fc1025	GCGCTA	2,750,451	female
MRD30x60.82	6570	144.84	145.37	129.35	6	B	fc1025	TAATCG	4,372,706	hermaphrodite
MRD30x60.84	6572	184.49	184.98	170.29	7	C	fc1025	TACAGC	7,423,846	female
MRD30x60.88	6569	87.92	88.27	77.93	5	C	fc1025	TATAAT	2,452,805	female
MRD30x60.92	6567	78.32	78.65	68.91	1	D	fc1025	TCATTC	2,022,507	hermaphrodite
MRD30x60.94	6568	72.72	73.07	62.55	1	D	fc1025	TCCCGA	1,924,070	female
MRD30x60.95	6565	111.83	112.25	99.76	2	B	fc1025	TCGAAG	3,102,468	hermaphrodite
MRD30x60.100	6565	60.37	60.65	52.06	10	E	fc1025	TCGGCA	1,877,322	hermaphrodite

[a]Number of targeted polymorphisms with nonzero depth.

[b]Mean depth (coverage) among all targeted polymorphisms (SNPs and indels).

[c]Mean depth (coverage) among all targeted SNPs.

[d]Mean depth (coverage) among all targeted indels.

[e]Prior to gel extraction, libraries were pooled in groups of 4, as indicated.

[f]A total of 8 or 12 libraries were pooled for a single hybridization reaction, as indicated.
