# Supplementary material for: Targeted Sequence Capture Provides Insight into Genome Structure and Genetics of Male Sterility in a Gynodioecious Diploid Strawberry, Fragaria vesca ssp. bracteata (Rosaceae)
Source: G3 (Bethesda). 2013 Aug 1;3(8):1341–51. doi: 10.1534/g3.113.006288 (PMC3737174; doi:10.1534/g3.113.006288)
Supplement: Supporting Information [file supp_g3.113.006288_TableS2.pdf]

Table S2 Primer sequences and scaffold coordinates for nine informative polymorphic sites. These were identified from the low-coverage genome sequences of the parents on scaffold scB (scf0513158 positions 2,258,778-4,674,928). The phenotyped targeted capture offspring (N=40) and additional offspring from the cross (N=55) with known sexual phenotypes were genotyped at these informative sites by Sanger sequencing (Figure 5).

Locus	Scaffold coordinates[a]	Forward/ Reverse primer sequences (5' - 3')	Annealing temperature °C - Time
scB_1556471	1556327-1556351	For: GTGAATTACATGAAGGTGACTGGGG	54°C - 30 sec
	1556795-1556819	Rev: AAAAGGGGTCATCACATCTGGAAAC	
scB_1644279	1643952-1643977	For: ACTACCAACTAGAGACACACATTCCC	56°C - 30 sec
	1644581-1644605	Rev: GAGAAATGATCCAAGACAGAGAGGC	
scB_1675672	1675540-1675564	For: TATGAAGTGGCGTAGAGAATAGGGC	56°C - 30 sec
	1675835-1675859	Rev: TTAGCAACAACAAAACCCTGGACTC	
scB_1689672	1689392-1689416	For: TACGAGTTGCCTTTTGGAGGTTAGG	56°C - 30 sec
	1689726-1689750	Rev: TCAATTAGTCTTGGCCTTGCTTCTG	
scB_1711631	1711443-1711467	For: CTACAGACCAACACCGTCCAAATAC	56°C - 30 sec
	1711904-1711928	Rev: CTTCTTCTGACCTCTCTGACTTTGC	
scB_1731089	1730768-1730793	For: GCCCTGTTGTAGAAATGAATCTGGAG	56°C - 30 sec
	1731360-1731383	Rev: TTATTCCCCCTACTCACACTCAGC	
scB_1776904	1776703-1776727	For: TTTCATTCCTTCCGACTAAAGCTGC	56°C - 30 sec
	1777051-1777076	Rev: CCACTCACTTTTAACCAGATGCTCAG	
scB_1804106	1803856-1803886	For: ATGGTAGCATAATGCCTCATATAACACTAG	56°C - 30 sec
	1804266-1804293	Rev: GTGAGGTTCTTGCCATCTTTATTCCAC	
scB_2027728	2027451-2027475	For: CAGTTTGTGTATGTGTTTGTTGCGG	56°C - 30 sec
	2028097-2028121	Rev: GCATTTAGATTTCCAAGCGTTAGCC	

[a]Coordinates are with respect to scB (add 2,258,777 to determine the position on scaffold scf0513158 in the *F. vesca* ssp. *vesca* reference genome version 1. 0).
