# Supplementary material for: Targeted Sequence Capture Provides Insight into Genome Structure and Genetics of Male Sterility in a Gynodioecious Diploid Strawberry, Fragaria vesca ssp. bracteata (Rosaceae)
Source: G3 (Bethesda). 2013 Aug 1;3(8):1341–51. doi: 10.1534/g3.113.006288 (PMC3737174; doi:10.1534/g3.113.006288)
Supplement: Supporting Information [file supp_g3.113.006288_TableS3.pdf]

Table S3 Incongruities between FvH4 and Fvb. Listed are scaffolds from FvH4 that map to Fvb linkage groups in ways incompatible with their assembly position in FvH4. Some of these likely represent assembly errors, while others likely represent real translocations or inversions.

Scaffold	Pseudochromosome	Start	End	Linkage_Group	Entire scaffold?[a]	Type
scf0512942	FvH4_0	317810	486710	4	yes	assignment of unanchored scaffold
scf0512960	FvH4_0	771617	922450	5	yes	assignment of unanchored scaffold
scf0512975	FvH4_0	932451	1031722	4	yes	assignment of unanchored scaffold
scf0513018	FvH4_0	1199431	1488070	2	yes	assignment of unanchored scaffold
scf0513028	FvH4_0	1498071	1647224	4	yes	assignment of unanchored scaffold
scf0513041	FvH4_0	2252495	2721037	1	yes	assignment of unanchored scaffold
scf0513045	FvH4_0	2731038	2792572	5	yes	assignment of unanchored scaffold
scf0513068	FvH4_0	2802573	3345236	2	yes	assignment of unanchored scaffold
scf0513081	FvH4_0	4365948	5638289	1	yes	assignment of unanchored scaffold
scf0513119	FvH4_0	5648290	6841401	2	yes	assignment of unanchored scaffold
scf0513137	FvH4_0	7326933	8406320	2	yes	assignment of unanchored scaffold
scf0513148	FvH4_0	9021620	9922368	2	yes	assignment of unanchored scaffold
scf0513155	FvH4_0	9932369	11179145	4	yes	assignment of unanchored scaffold
scf0513189	FvH4_0	11189146	11767950	1,2	no	assignment of unanchored scaffold, interchromosome translocation
scf0513151	FvH4_1	1	1445376	4,1	no	interchromosome translocation, inversion
scf0513105	FvH4_1	1455377	3331343	1	yes	inversion
scf0513114	FvH4_1	3341344	4120631	5	yes	interchromosome translocation
scf0512959	FvH4_1	4829947	5419653	1,5	no	inversion, interchromosome translocation
scf0513095	FvH4_1	13186592	14572914	6	no	interchromosome translocation
scf0513168_1	FvH4_1	17339065	19938125	3,6	no	interchromosome translocation (x2)
scf0512938	FvH4_2	2272727	2456317	2	yes	intrachromosome translocation
scf0513057	FvH4_2	5704770	6000074	4	yes	interchromosome translocation
scf0512968	FvH4_2	17482696	18535496	2	yes	inversion
scf0513134	FvH4_2	18667366	21445916	2	yes	inversion
scf0513194	FvH4_2	21455917	24538926	2	yes	inversion
scf0513171	FvH4_3	1	1497153	3	yes	inversion
scf0513104	FvH4_3	1507154	3362024	3	yes	inversion
scf0513072	FvH4_3	3372025	3727613	6	yes	interchromosome translocation
scf0513017	FvH4_3	3737614	4232872	3	yes	inversion
scf0513082	FvH4_3	4410664	5334373	3	yes	inversion
scf0513173	FvH4_3	6456995	8718722	3	yes	inversion
scf0513089	FvH4_3	9045060	9383133	7	yes	interchromosome translocation

scf0513156	FvH4_3	9809969	11035985	7	yes	interchromosome translocation
scf0513115	FvH4_3	11045986	11903823	5	yes	interchromosome translocation
scf0513053	FvH4_3	15204652	15608968	4	yes	interchromosome translocation
scf0513125	FvH4_3	19509464	21597487	2	yes	interchromosome translocation
scf0513015	FvH4_3	27880400	28601208	3	yes	intrachromosome translocation
scf0513029	FvH4_3	30982798	31368128	3	yes	inversion
scf0513065	FvH4_4	1	206650	6	yes	interchromosome translocation
scf0513090	FvH4_4	7833870	8595096	3	yes	interchromosome translocation
scf0513124_4	FvH4_4	9525261	10260387	6	no	interchromosome translocation
scf0513150	FvH4_4	12599598	14043386	5	no	interchromosome translocation
scf0512935	FvH4_4	14053387	15031722	4	yes	intrachromosome translocation
scf0513004	FvH4_4	22938187	23936647	6	no	interchromosome translocation
scf0513025	FvH4_4	23946648	24163290	4	yes	inversion
scf0513158_4	FvH4_4	24298703	26557413	7	no	interchromosome translocation
scf0513012	FvH4_5	1	128260	3	yes	interchromosome translocation
scf0513098	FvH4_5	138261	3585757	6	no	interchromosome translocation
scf0513052	FvH4_5	3595758	3984026	2	yes	interchromosome translocation
scf0511962	FvH4_5	5833229	5933781	5	yes	intrachromosome translocation
scf0513135	FvH4_5	8572001	11121459	5	yes	inversion
scf0513066	FvH4_5	13220304	13294433	1	yes	interchromosome translocation
scf0513166	FvH4_5	14797581	15196668	5	yes	intrachromosome translocation
scf0513094	FvH4_5	17913724	20360920	5	yes	inversion
scf0513146_5	FvH4_5	20370921	21946032	1	no	interchromosome translocation
scf0513187	FvH4_5	23614770	26212222	4	no	interchromosome translocation
scf0512965	FvH4_5	26222223	26854803	5	yes	intrachromosome translocation
scf0513087	FvH4_5	27369861	27940252	4	no	interchromosome translocation
scf0512963[b]	FvH4_5	27950253	28286745	5	yes	intrachromosome translocation
scf0513011[b]	FvH4_5	28296746	28438568	5	yes	intrachromosome translocation
scf0513168_6	FvH4_6	213555	1544462	5,6	no	interchromosome translocation, inversion
scf0512983	FvH4_6	1554463	1973909	6	yes	intrachromosome translocation
scf0513061	FvH4_6	1983910	3376700	6	yes	inversion
scf0513177	FvH4_6	4210475	8315614	6	yes	inversion
scf0513185	FvH4_6	11066533	11863480	7	yes	interchromosome translocation
scf0513196	FvH4_6	13162357	15497570	2	no	interchromosome translocation
scf0513176	FvH4_6	16117078	18575326	1	no	interchromosome translocation
scf0512952	FvH4_6	19487000	19734822	5	no	interchromosome translocation

scf0513040	FvH4_6	25990763	26193578	4	yes	interchromosome translocation
scf0512991	FvH4_6	34044179	36036831	1, 6	no	interchromosome translocation, intrachromosome translocation
scf0512961	FvH4_6	36046832	36389855	6	yes	intrachromosome translocation
scf0513112	FvH4_6	38173164	39347594	2,6	no	interchromosome translocation, inversion
scf0513170	FvH4_7	1	5155874	1,3,7	no	interchromosome translocation (x2), intrachromosome translocation
scf0513080	FvH4_7	7699732	8246547	5	no	interchromosome translocation
scf0512990[c]	FvH4_7	11408502	12404147	7	no	inversion
scf0512946[c]	FvH4_7	12414148	13183493	7	no	inversion
scf0513113[d]	FvH4_7	15242379	16658559	6,7	no	interchromosome translocation, inversion
scf0513044[d]	FvH4_7	16668560	19276478	2,6	no	interchromosome translocation (x2)
scf0513190	FvH4_7	19286479	22556666	7	yes	inversion

[a]Indicates whether data are compatible with the entire scaffold, and only the entire scaffold, contributing to the incongruity. Such scaffolds may represent assembly errors.

[b]same translocation from FvH4_5 to a different part of Fvb5

[c] same inversion on FvH4_7

[d]same translocation from FvH4_7 to Fvb6
